# ENHYDROSS: A New Mechanistic Model Supports the Trans‐Oceanic Dispersal Capability of Terrestrial Vertebrates

**DOI:** 10.1002/ece3.73280

**Published:** 2026-03-30

**Authors:** Alexandros Pantelides, Paul Upchurch, Philip D. Mannion, Elias Gravanis, Donald M. Henderson, Phaedon Kyriakidis

**Affiliations:** ^1^ Department of Civil Engineering and Geomatics Cyprus University of Technology Limassol Cyprus; ^2^ Department of Earth Sciences University College London London UK; ^3^ Royal Tyrrell Museum of Palaeontology Drumheller Alberta Canada

**Keywords:** cost of transport, dispersal distance, mechanistic model, optimal swimming speed, terrestrial vertebrates, trans‐oceanic dispersal

## Abstract

Many terrestrial vertebrates, both extinct and extant, have widespread or even global distributions. Although vicariance (e.g., through continental fragmentation, sea‐level changes) explains some of these patterns, others seemingly require long‐distance trans‐oceanic dispersal. A key but underexplored factor in this debate is the biological feasibility of such dispersal based on an organism's physiology and biomechanics. We introduce ENHYDROSS, a new mechanistic energetic model that estimates optimal swimming speed and minimum cost of transport for any vertebrate. These allow us to estimate maximum swimming distances and durations. We tested ENHYDROSS on two mammals (elephant, polar bear) and five reptiles, including the Aldabra giant tortoise, saltwater crocodile, ostrich, and two extinct nonavian dinosaurs (*Lambeosaurus* and *Rapetosaurus*). For the extinct dinosaurs, we used a broad range of basal metabolic rates to account for different thermophysiological hypotheses. The model's estimates for extant animals align with observed data, while cases of underestimates can be attributed to the effects of ocean currents, as evidenced by estimated passive drifting distances and times under predominantly mild and intermediate currents. ENHYDROSS generally predicts greater swimming capacity than previously proposed models due to assumptions like null‐thermogenesis, resulting in lower minimum cost of transport. Applying our model to test the feasibility of extinct dinosaur dispersal between Africa and Europe during the Cretaceous via the Alboran route (the oceanic corridor separating Iberia from Morocco), we found that both hadrosaurs and titanosaurs could plausibly complete the journey, particularly under favorable conditions such as low sea levels, stepping‐stone islands, and higher fat reserves. Hadrosaurs showed slightly better swimming efficiency. Dispersal was especially feasible during the early–middle Albian (112.5–107.5 Ma) and latest Cretaceous (72.5–66 Ma), but was unlikely during periods of high sea levels (97.5–77.5 Ma). These results support the possibility of trans‐oceanic dinosaur dispersal across distances of up to ~560 km.

## Graphical abstract

We introduce ENHYDROSS, a new mechanistic model that uses optimal swimming speed and minimum cost of transport to estimate maximum dispersal distances and durations for vertebrates, enabling assessment of long‐distance oceanic dispersal potential. Applied to a range of extant and extinct animals, the model's estimates generally align with observed data; underestimates can often be explained by ocean currents aiding passive drift. These findings support the feasibility of previously hypothesized trans‐oceanic dispersal by hadrosaurs and titanosaurs between Africa and Europe via the Alboran route during the Cretaceous.

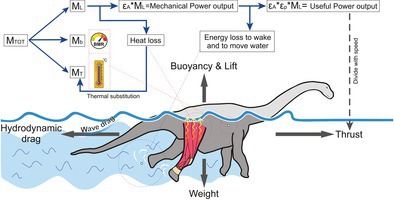

## 1 Introduction

Historical biogeography attempts to explain the distributions of organisms via phenomena such as vicariance and dispersal (Brooks and McLennan 2002; Cowie and Holland 2006; Lomolino et al. 2010; Nelson and Platnick 1981; Sanmartín 2012). Trans‐oceanic dispersal (also known as overseas dispersal or sea crossing) is a special case of long‐distance dispersal (de Queiroz 2005; Nathan and Nathan 2014), where terrestrial organisms cross substantial overseas distances, often implying a role for rare or unpredictable events (e.g., storms blowing migrating animals off course). Such dispersals have been observed or inferred in a variety of organisms including plants (e.g., Nathan 2006), invertebrates (e.g., Carlton et al. 2017), and vertebrates (usually of small size) (e.g., Antoine et al. 2012; Calsbeek and Smith 2003; Carranza and Arnold 2003; Censky et al. 1998; Longrich et al. 2021; Marivaux et al. 2023; Poux et al. 2005; Pyron 2014; Raxworthy et al. 2002; Sanmartín and Ronquist 2004; Upchurch 2008; Welt and Raxworthy 2022; Yoder et al. 2003). Traditionally studied through a biogeographic lens, trans‐oceanic dispersal is currently undergoing intense scrutiny (e.g., Ali et al. 2021; Ali and Hedges 2023; Ali and Huber 2010; Ali and Vences 2019; Gillespie et al. 2012; Heads 2014; Masters et al. 2021; Mazza 2014; Mazza et al. 2019, 2013; Nathan et al. 2003; Nathan and Nathan 2014; de Queiroz 2005, 2014; Thiel and Gutow 2005a, 2005b; Thiel and Haye 2006; van der Geer et al. 2015). The vast majority of studies have focused on the rafting subcategory of trans‐oceanic dispersal and thus on small animals that can be transported by vegetation mats or logs (e.g., Ali et al. 2021; Ali and Vences 2019; Carlton et al. 2017; Censky et al. 1998; Houle 1998; King 1962; MacFadden 1980; Masters et al. 2021; Mazza et al. 2019; Meijaard 2005; Thiel and Gutow 2005a, 2005b; Thiel and Haye 2006). However, trans‐oceanic dispersal via active swimming and/or more passive current‐driven floating has also been invoked to explain some biogeographic distributions, especially with regard to larger‐bodied organisms (Aziz 2000; Frantz et al. 2018; Hadjisterkotis 2012; Hadjisterkotis et al. 2000; Johnson 1978; Longrich et al. 2021; Sallam et al. 2018; van den Bergh et al. 2001; van der Geer et al. 2011, 2015). Trans‐oceanic dispersal hypotheses may be controversial because researchers dispute the ability of taxa to cross a given region of ocean, or because putative land routes (which themselves may be debated) offer a more feasible corridor for dispersal (e.g., de Queiroz 2005; Groh et al. 2023; Heads 2005; Masters et al. 2021; Sanmartín and Ronquist 2004; Upchurch 2008, In press). Thus, the phenomenon of trans‐oceanic dispersal raises questions about the maximum feasible swimming distances and durations of organisms, especially terrestrial ones that are not strongly adapted for an aquatic environment. The issues surrounding trans‐oceanic dispersal have been approached in several different ways, including: inferences based on phylogenetic relationships and/or the fossil record; experimental data on the swimming abilities of organisms; direct observations of extant dispersing organisms; and modeling techniques that draw on some or all of these other approaches (see below).

The inherent quasi‐random nature of trans‐oceanic dispersal events means that, as Gillespie et al. (2012) noted, it is only possible to predict aspects of this phenomenon over extended (evolutionary) time periods (see also Darlington 1938). Consequently, the current research trend is to focus on inferring such dispersals (i.e., distributional evidence) in the past using phylogenetic biogeographic approaches (e.g., Groh et al. 2023; Longrich et al. 2021) or measuring colonization frequencies on evolutionary timescales (e.g., Ali and Hedges 2023). By contrast, experimental data and direct observations in the wild are somewhat rarer. One of the first studies that attempted to assess the survivability of terrestrial animals within aquatic environments, was based on experiments on the floatation ability of *Anolis sagrei* lizards (Schoener and Schoener 1984). These workers collected data on the distances covered by organisms while swimming, floating, and rafting, and presented experimental data from small animals that quantified the limits on their swimming durations. This study was, as far as we are aware, the first to connect biogeography, experimental data, and observations in the wild on swimming endurance of terrestrial animals. Unfortunately, pragmatic constraints meant that Schoener and Schoener's (1984) experimental data only applied to relatively small organisms (typically less than 5–10 kg), and data for wild animals are scarce (see Section 2.2). This scarcity of relevant studies for larger extant vertebrates may reflect a relatively low probability of long‐distance trans‐oceanic dispersal, especially when the window of observation is very short in terms of geological or evolutionary timescales.

What if one was to model how far, or how long, an animal could travel and therefore make predictions about the feasibility of dispersal? This type of mechanistic modeling would allow individual cases of trans‐oceanic dispersal to be evaluated from a new angle, and could feedback information on feasibility into the more traditional macroevolutionary techniques that utilize phylogenetic relationships, divergence time estimations, fossil data, etc. Modeling the aquatic dispersal abilities of terrestrial organisms, at least in the context of historical biogeography, has hitherto received relatively little attention. Bottom‐up mechanistic approaches of this type usually fall under the remit of movement ecology and biomechanics and are often related to the study of migrations or locomotion in general. Such works typically investigate maximum migration distances, movement speeds, and energetics (e.g., Alerstam 1991; Alerstam et al. 2003; R. M. Alexander 1999; R. McN. Alexander 1998; Braithwaite et al. 2015; Gough et al. 2019; Hancock and Hedrick 2018; Hedenström 2003; Hein et al. 2012; Prange 1976; Tanaka et al. 2005; Tucker 1971; Weber 2009; Weihs 1973). Swimming and floatation have largely been investigated in fully aquatic taxa (e.g., Blake 1980, 1979; Feldkamp 1987; Fish 2020, 2002; Fish and Lauder 2006; Gough et al. 2021; Gutarra et al. 2023, 2022, 2019; Hind and Gurney 1997; Lauder and Tytell 2005; Videler and Nolet 1990; Webb and Blake 1985; Williams 2022, 1999, 1989; Zhan et al. 2017) or semi‐aquatic ones (e.g., Aigeldinger and Fish 1995; Coughlin and Fish 2009; Fish 2000, 1996a, 1993, 1984a, 1984b, 1982; Fish et al. 1997; Fish and Baudinette 1999; Gough et al. 2015; Hood 2020; Videler and Nolet 1990). By contrast, swimming locomotion in terrestrial animals (especially large‐bodied taxa) has received far less attention, with the focus often confined to animals of commercial value such as dogs (e.g., Fish, DiNenno, and Trail 2021) or horses (e.g., Santosuosso et al. 2022, 2021; Thomas et al. 1980). Although there have been studies that have reduced this relative knowledge gap to some extent (e.g., Blanco 2023; Dagg and Windsor 1972; Fish 1993, 1996a; Henderson 2004, 2014, 2018; Henderson and Naish 2010; Mallon et al. 2018; Meijaard 2001; Schoener and Schoener 1984; Sereno et al. 2022; Wilson 1974), it continues to hinder the formulation of a bottom‐up mechanistic approach to the trans‐oceanic dispersal of large vertebrates. Consequently, the only energetic mechanistic model applied to trans‐oceanic dispersal in vertebrates, other than humans (e.g., Hölzchen et al. 2022, 2021), is that of Meijaard (2001). This model was recently applied by (Hertler et al. 2025, 2022) in a spatially explicit form, testing the feasibility of sea‐crossing routes for the extinct proboscidean *Stegodon* in the Indonesian archipelago. This approach (Hertler et al. 2025, 2022) also accounted for bioclimatic variables such as sea currents—one of the key gaps in Meijaard's original model (see Data S1). This has opened the door for a more thorough testing of hypotheses, such as: (1) the feasibility of crossing a body of water given an organism's energy reserves and the effect of sea currents; (2) the probability of crossing an area of ocean of a given width; and (3) the probability of establishing a viable founder group at a destination. However, such results are only as accurate as their underlying model (i.e., Meijaard 2001), which, as discussed in Data S1, is preliminary, not taxonomically universal, and potentially highly inaccurate.

A general energetic model for transoceanic dispersal is still lacking. Here we present and evaluate a more general model of transoceanic dispersal in which we overcome many of the problems inherent in Meijaard's (2001) preliminary model. This results in a mechanistic model that captures the hydrodynamics and energetics of any group of vertebrates, under variable biotic factors (i.e., hydrodynamic drag, locomotory style, size, energy reserves, metabolism, etc.). We then apply this model to five extant taxa for validation, comparing the results with Meijaard's (2001) preliminary model and three variants of it (see Data S5). Finally, we apply the model to two extinct non‐avian dinosaurs to test the feasibility of trans‐oceanic dispersal via the Albora route, an oceanic route between Europe (Iberia) and Africa during the Cretaceous Period.

## 2 Materials and Methods

### 2.1 Derivation of the ENHYDROSS Mechanistic Model

#### 2.1.1 Justification of Choice of Optimality Criterion

An important question, that is often pursued in studies modeling migration and dispersal, is whether animal movement is governed by some optimality criterion. There is more than one definition of optimal locomotory speed depending on the costs and benefits to be optimized. These issues have been explored extensively for activities such as migration with or without foraging, and minimizing risk of predation (e.g., Alerstam 1991; R. McN. Alexander 1998; Pyke 1981; Videler and Nolet 1990; Weihs 1973). For example, in some cases it might be beneficial for an animal to travel as quickly as possible to cover a given distance (“time minimizer” optimality) (Alerstam 1991; Pyke 1981). In other cases, it might be that an animal needs to move at a speed that minimizes its cost of transport (COT) so it can travel the furthest (“energy cost per unit distance minimizer” optimality) (Alerstam 1991; Pyke 1981). Alternatively, it might travel at a speed whereby the COT maximizes the duration of travel, rather than absolute distance, while performing a particular activity such as foraging or maintaining flight (“time maximizer” or equivalently “energy cost per unit time minimizer” optimality) (Alerstam 1991; Pyke 1981). Therefore, one might ask which of these optimality criteria best fits the movement involved in trans‐oceanic dispersal? For terrestrial animals that swim to cross a water body, foraging and refueling stopovers are often not possible, limiting the range of applicable criteria. When crossing a relatively short stretch of water, where the opposite shore is visible, minimizing swimming time may be advantageous to reduce predation risk or other ocean‐related stresses. For the much longer distances typically involved in trans‐oceanic dispersal, the most suitable optimality criterion is probably the one used in Weihs' (1973) for optimal cruising speed for migrating fish, because it maximizes distance traveled per unit of energy spent or equivalently minimizes the COT (energy expenditure per unit distance). The latter criterion has been used by many previous studies (Gough et al. 2019; Hertler et al. 2025, 2022; Meijaard 2001; Motani 2002; Villamil et al. 2016) and it is central to the model we develop in this paper.

In the context of trans‐oceanic dispersal, it is crucial to know whether cruising speed follows an optimality criterion. This is because, although the preferred cruising speed for an animal might not be known, the optimal speed can be calculated under certain conditions from first principles (e.g., R. McN. Alexander 1998; Motani 2002; Sato et al. 2010; Weihs 1973) and this in turn can be used to estimate the cost of transport and hence the distance the animal can cover or the time it requires to travel a given distance. Current evidence suggests that many animals do indeed move at or close to optimal speed under different conditions, for example, underwater swimming in penguins (Sato et al. 2010), as well as surface paddle‐swimming in mallard ducks (Prange and Schmidt‐Nielsen 1970) and swimming in non‐feeding basking sharks (Sims 2000). In addition, sea turtles and flying birds travel close to optimal speeds (Prange 1976; Tucker 1971) when migrating long distances. On the other hand, the preferred swim speeds (0.58 m/s) of muskrats is below optimal speed (0.75 m/s); probably for reasons related to fatigue, where anaerobic respiration must be invoked to maintain the optimal speed, and so it is not sustainable (Fish 1982). However, this muskrat case seems to be the exception to the rule, as illustrated by many more examples of organisms swimming close to their optimal speeds (e.g., see data presented in Alerstam 1991; R. McN. Alexander 1998; Pyke 1981; Videler and Nolet 1990). Thus, while the question remains open, there are good reasons to assume that many animals swim at *U*_opt_ under certain conditions.

In short, here we focus on estimating the maximum feasible trans‐oceanic dispersal distance and thus we use the optimization criterion of minimum cost of energy per unit distance traveled.

#### 2.1.2 Main Components of the Model

In the context of trans‐oceanic dispersal, there are two key values that we would like to know for a given organism: (1) maximum dispersing distance; and (2) its corresponding dispersal duration (N.B. we avoid using the term “maximum dispersal duration” to avoid confusion with the respective optimality “time maximizer” criterion mentioned in Section 2.1.1). This is because the furthest an animal can swim can be used to evaluate the feasibility of dispersal from one landmass to another. The corresponding swimming duration, in turn, can inform as to whether a given travel distance is feasible in the context of, for example, food or water deprivation. These parameters can be calculated using the following equations:

(1) $$\mathrm{Max}\;\text{swimming distance}=\frac{\text{Available energy}}{{\mathrm{COT}}_{\min}}$$

(2) $${\text{Swimming time}}_{\max\_\text{distance}}=\frac{\mathrm{Max}\;\text{swimming distance}}{U_{\mathrm{opt}}}$$

where COT_min_ is the minimum cost of transport defined as the energy expended to travel a unit distance (N.B. when referring to COT, most researchers mean the energy expended to move a unit mass over a unit of distance, which is referred to as the “mass‐specific COT,” whereas in our case the COT is mass‐independent), and *U*_opt_ is the optimal swimming speed that corresponds to that COT_min_. The energy utilized by the organism for all its needs during its swimming journey is assumed to come solely from fat. The energy equivalent in Joules can be obtained by multiplying the fat mass (kg) by the energy density of 39 MJ per kg, and then taking 23% of that value to account for the conversion efficiency (Meijaard 2001 and references therein).

There are three points to note regarding the above equations. First, using them we avoid the need to calculate an intermediate step used by previous studies (Hertler et al. 2022; Meijaard 2001) (see Data S1). Second, from Equation (1) we can see that the available energy is directly proportional to the maximum swimming distance and duration. In effect, this means that one needs no sensitivity tests to see that swimming distance and duration will increase linearly with fat mass. Third, for our present purposes, dispersal distance and time are synonymous with swimming distance and time. Note that if one wishes to adjust Equations (1), (2) for a drifting as opposed to a swimming animal, one would only need to substitute *U*_opt_ with the relative speed of transport (e.g., ocean current net velocity), and to calculate COT_min_ one would only require the basal metabolic rate divided by that speed (at least when thermoneutral conditions apply).

Equations (1) and (2) indicate that we need to estimate: (1) the optimal swimming speed (*U*_opt_); (2) the minimum cost of transport (COT_min_) which corresponds to that optimal swimming speed; and (3) available energy stored inside the swimmer's body which means that one needs to know the fat (adipose) mass. Ideally, COT_min_ should be calculated using *U*_opt_, or vice versa, because they depend on each other. Given that COT_min_ depends (among other factors) on *U*_opt_, and because *U*_opt_ is unknown, the logical and easiest approach is to first derive an equation for estimating *U*_opt_.

#### 2.1.3 Optimal Swimming Speed Calculation

**FIGURE 1:**
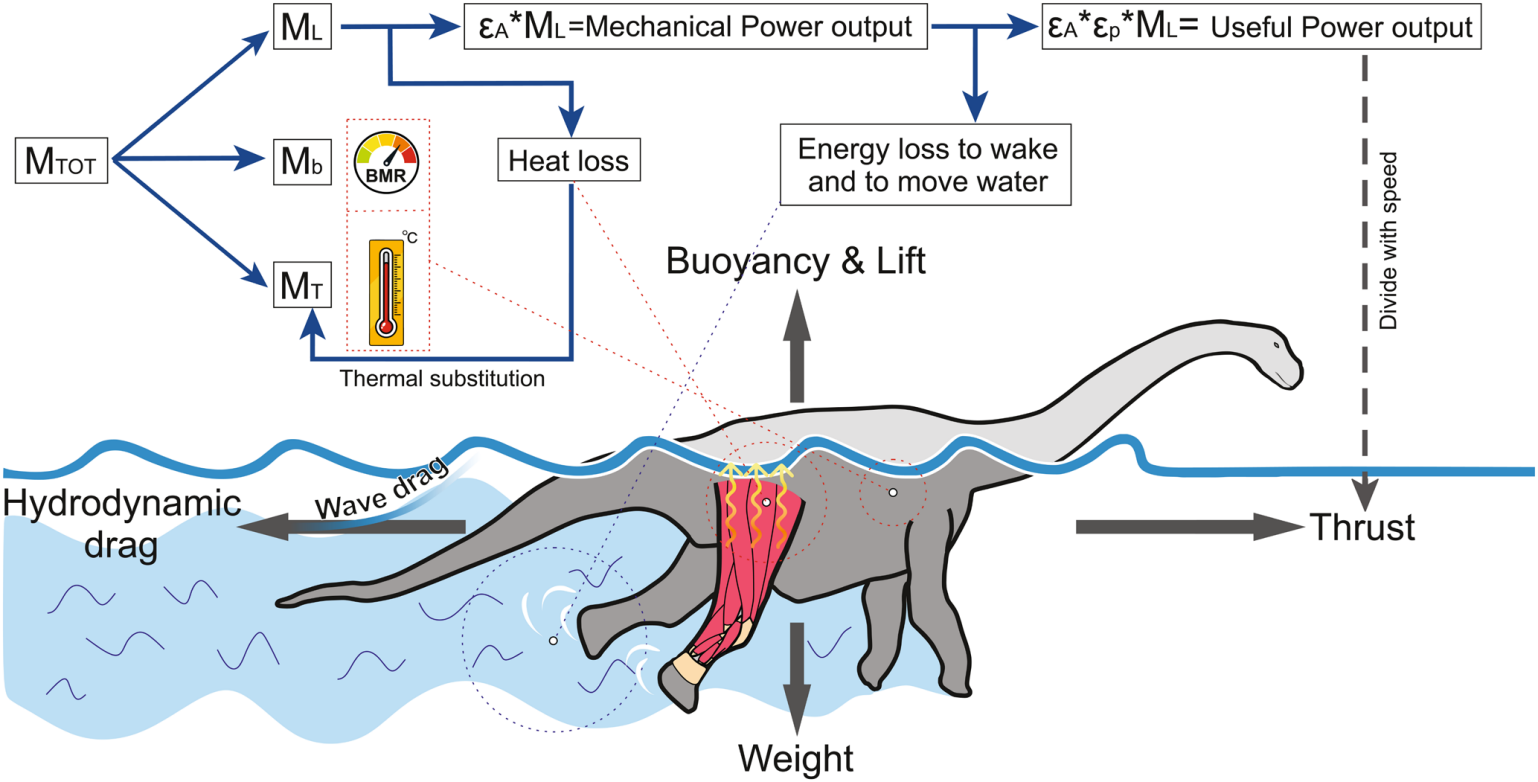
Top: Power balance and energy flow (blue arrows) showing how power diminishes until a swimmer produces useful power (also known as thrust or drag power) to overcome drag and achieve movement. For an endothermic animal, a percentage from the heat loss produced by the inefficiency of muscles during locomotion stays trapped within the body aiding the animal in keeping its temperature constant by saving energetic cost that would have otherwise been used for thermogenesis—a phenomenon known as “thermal substitution.” Bottom: Schematic diagram showing the main forces (dark gray arrows) acting on a vertebrate swimming at a constant speed at the surface of water, represented by a titanosaurian sauropod. Note that during surface swimming, wave drag becomes an additional component of hydrodynamic drag above a certain speed. *M*_TOT_: Total metabolic power; *M*_b_: Basal metabolic rate (BMR); *M*_L_: Metabolic cost of locomotion; *M*_T_: Metabolic cost of thermogenesis to maintain a constant body temperature; *ε*_A_ is the aerobic efficiency of the animal or the muscles; *ε*_p_ is the propulsive efficiency associated with the swimming style of the animal; Dividing useful power by speed (dashed gray arrow) results in the thrust force exerted by the animal while swimming, which is equal to the drag force when swimming speed is constant.

Because of several problems identified in Meijaard's (2001) approach (See Data S1), we avoid using the optimal speed empirical approximation employed in that study. Instead, we utilize a more fundamental model that still retains empirical parameters but is grounded in a more robust theoretical foundation. We follow Motani's (2002) steps, re‐working an optimal swimming speed equation starting from the energy balance equation for a living animal. Using the same symbology as Motani (2002):

(3) $$M=M_{b}+M_{T}+M_{L}$$

where *M* is the total metabolic rate or power, *M*_b_ is the basal metabolic rate (BMR), *M*_T_ is the cost of thermogenesis to keep the animal's temperature constant compared to the environment, and *M*_L_ is the cost of locomotion (i.e., the power output required solely for movement) with all parameters measured in Watts (Figure 1). We first find the total mass‐independent cost of transport (*sensu* Motani 2002; Williams 1999) for an organism by dividing Equation (3) by speed as:

(4) $${\mathrm{COT}}_{\mathrm{TOT}}=\frac{\text{Total Metabolic Rate}}{U}=\frac{M_{b}+M_{T}+M_{L}}{U}$$

where COT_TOT_ is the total cost of transport in J/m following Motani (2002) (see also Williams 1999) and does not have the same definition as the one typically encountered in the literature which has units as J/kg/m and is formally known as mass‐specific cost of transport. We will use the total COT definition in this study for convenience: hence, COT_min_ and COT_TOT_ (total COT or just COT) denote the minimum cost and total cost of transport, respectively, with the same J/m units, only differing in the velocity (optimal or not). COT (J/m) can easily be converted to the mass‐specific COT (J/kg/m) by dividing by each animal's body mass whenever necessary.

*M*_*L*_ is defined by Hind and Gurney (1997) as:

(5) $$M_{L}=\frac{0.5\rho \lambda SC_{d}}{\varepsilon_{p}\varepsilon_{A}}U^{3}$$

where *ε*_A_ is the aerobic efficiency of the animal or the muscle (see Section 2.4.2), *ε*_p_ is the propulsive efficiency of the swimming mode (see Section 2.4.3), *ρ* is the density of water, *λ* is the general correction factor of the equation or alternatively the active/passive drag ratio (which may or may not be used i.e., *λ* = 1) in the calculation because its justification and interpretation are not well defined yet (see Section 2.4.4), *S* is the surface area of the body (wetted, frontal or otherwise depending on one's choice; see Sections 2.1.5 and 2.4.1), and C_*d*_ is the drag coefficient (see Section 2.1.5).

Here, we are interested in the special case where *U*=*U*_opt_ which is where COT_TOT_ = COT_min_ by our definition of optimality. The function COT_TOT_ has a minimum where its derivative is zero. Thus, if we take the derivative of Equation (4) we obtain the equation for the *U*_opt_:

(6) Uopt=εpεAMb+MTρλSCd13

In the special case where no additional metabolic heat production is required to offset heat loss to the environment, we set *M*_T_ = 0 *sensu* Motani (2002). This condition of null‐thermogenesis applies under any of the following three biologically distinct but energetically equivalent scenarios: (1) the animal operates under thermoneutral conditions in water, such that no thermoregulatory heat production is necessary; (2) the animal is an ectotherm, for which *M*_T_ = 0 by definition; or (3) the animal is an endotherm in which heat lost to the environment is fully compensated by heat generated during muscular activity, a process known as “thermal substitution” (Humphries and Careau 2011; Lovvorn 2007). Under these assumptions, Equation (6) simplifies to:

(7) Uopt=εpεAMbρλSCd13

This equation has been derived and/or cited in a number of papers (e.g., R. M. Alexander 1999; Blanco 2023; Gough et al. 2019; Gutarra and Rahman 2022; Motani 2002; Sato et al. 2010; Villamil et al. 2016), but see Data S2 for its derivation and some remarks.

#### 2.1.4 Minimum Cost of Transport Calculation

Back‐substituting Equations (5), (6) into Equation (4) gives the minimum cost of transport (in Joules/m):

(8) $${\mathrm{COT}}_{\min}=\frac{3}{2}\frac{\left(M_{b}+M_{T}\right)}{U_{\mathrm{opt}}}$$

A stepwise derivation of Equation (8) can be found in Data S2. If, as previously stated, any of the conditions of null‐thermogenesis apply (thermoneutrality, ectothermy, or endothermy with complete thermal substitution), then *M*_T_ = 0. Hence, COT_min_ becomes:

(9) $${\mathrm{COT}}_{\min}=\frac{3}{2}\frac{M_{b}}{U_{\mathrm{opt}}}$$

By deriving Equations (8) and (9) we have provided something that, to our knowledge at least, was previously missing from the literature. The connection between COT_min_, *U*_opt_, and metabolism is now fully established on theoretical grounds and the problem of linkage between COT_min_ and *U*_opt_ (see Data S1) is now solved.

#### 2.1.5 Drag Coefficient Calculation

##### 2.1.5.1 Drag Coefficient Formulae

The drag coefficient (*C*_d_) is a dimensionless measure of the overall resistance (or “dragginess”) of an object moving through a fluid, given by the relation *C*_d_ = 2*D*/(*ρ*S*u* ^2^), where *D* is the drag force, *ρ* the fluid density, *u* the object's velocity and *S* is a reference surface area of the object. If one knows the values of the variables in this equation, one can calculate the drag coefficient precisely, although in practice this is more difficult (for explanation see Vogel 1996, 89–90). The velocity of the object is what we would like to know, and the drag force cannot be determined without experiment: hence, we cannot calculate the drag coefficient using this definition. For cases where such direct calculation (via experiment or Computational Fluid Dynamics) is not possible, engineers using experimental procedures have derived approximation formulae based on simple geometrical shapes such as spheres, spheroids, and cylinders (e.g., Hoerner 1965, 6–17, 6–18 Equation 28 and equation 31). However, these formulae were derived based on the choice of reference surface area (*S*) and thus they are different expressions and will give different results. The reference surface area (*S*) can be interpreted in various ways, especially for irregularly shaped objects like animals. For swimming, three common options are: (1) the wetted surface area (total area exposed to flow), (2) the frontal or cross‐sectional area (projected perpendicular to flow) and (3) the volume^2/3^ (Vogel 1996, 90–91). D. E. Alexander (1990) showed that *C*_d_ values can vary markedly depending on whether one chooses the wetted surface area or the frontal area of a body. This is related to skin friction and how its ratio to pressure drag varies by changing the Reynolds number (see Section 2.1.6 for its definition) or the shape of the body. In general, when using the frontal surface area, short and blunt bodies will result in lower drag values, whereas elongated bodies will have lower drag values when the wetted surface area is used (see D. E. Alexander 1990; Vogel 1996, 90–91). D. E. Alexander (1990) noted that at low Reynolds numbers, where viscous forces dominate and shape and orientation have little impact on flow, using frontal area as a reference is reasonable; however, at high Reynolds numbers, where pressure drag and streamlining become more important, wetted area provides a better scale factor. On the other hand, Vogel (1996, 90–91) stated that the frontal area is suitable for nonstreamlined objects of high drag at high and medium Reynolds numbers, whereas wetted area is best for streamlined objects where viscous and shear forces dominate. At intermediate Reynolds numbers, the situation depends on body shape and the presence or amount of turbulence in the fluid (D. E. Alexander 1990). Nonetheless, as D. E. Alexander (1990) noted, the choice of surface areas used to calculate *C*_d_ is arbitrary and can be a matter of convenience, or selected to match the specifics of a particular case. Following D. E. Alexander's (1990) recommendation, for comparative reasons, we opt to calculate the drag coefficient based on two different surface areas: the wetted and the frontal surface areas (see Sensitivity tests). To do so, we provide two such approximation equations of drag coefficient (*C*_d_) for a prolate spheroid (Hoerner 1965, 6–17, 6–18 Equation 28 and equation 31) based on the wetted surface area (*S*_wetted_):

(10) $$C_{d_{\text{wetted}}}=C_{f}\left[1+1.5{\left(\frac{Z}{L}\right)}^{1.5}+7{\left(\frac{Z}{L}\right)}^{3}\right]$$

and based on the frontal (cross sectional) area (*S*_frontal_) of a body:

(11) $$C_{d_{\text{frontal}}}=C_{f}\left[3\left(\frac{L}{Z}\right)+4.5{\left(\frac{Z}{L}\right)}^{0.5}+21{\left(\frac{Z}{L}\right)}^{2}\right]$$

where *C*_f_ is the skin friction coefficient (see below), *Z* is the maximum body width of the animal body (i.e., the maximum diameter excluding limbs) and *L* is the characteristic body length, usually taken as the total length for fully submerged swimmers. However, because here we will be modeling surface swimming (which really means partially submerged swimming), the submerged Length (*L*_s_) is taken instead. It is important to emphasize that when choosing between Equations (10) and (11), one must remain consistent with the reference surface area type used in Equations ((5), (6), (7)).

Note that the ratio *Z*/*L* is equal to the inverse of the finesse ratio, defined as the ratio of body length to maximum width, that is, *L*/*Z*. This merely shows that the *C*_d_ depends on the finesse ratio but this is an oversimplification as other things affect the *C*_d_ too (e.g., appendage size and shape, position of the maximum diameter along the length of the animal etc.) (for more details regarding the factors affecting *C*_d_ see: Gutarra and Rahman 2022; Hoerner 1965). Thus, formulae (10) and (11) retain only a small fraction of the total geometrical information of the real body, in the form of the inverse of the finesse ratio. This would have been sufficient if our animals were shaped like a prolate spheroid, but they are not. In this respect, this formula only serves as a crude approximation of the shape of an animal, and thus complex, nonhydrodynamic forms with large, poorly streamlined appendages such as those of terrestrial vertebrates, are bound to be represented inaccurately. To partially mitigate this limitation, we modified *Z* to an equivalent diameter *Z*_eq_ (Table 1) to standardize the limb‐induced increase in drag across all modeled animals. To compute *Z*_eq_, one equates the submerged frontal area of the body, including the limbs, to the area of a circle. The diameter of this circle is the equivalent diameter *Z*_eq_, which can then be used to calculate *C*_d_ via Equations (10) and (11) for a partially submerged swimmer. By doing so, we ensured that the influence of poorly streamlined limbs, which would otherwise be neglected, was incorporated consistently into our drag estimates.

**TABLE 1:** Data input for the ENHYDROSS model.

	*Lambeosaurus lambei*	*Rapetosaurus krausei*	*Struthio camelus*	*Elephas maximus*	*Ursus maritimus*	*Aldabrachelys gigantea*	*Crocodylus porosus*
Length (*L*)	8.21	9.95	2.04	4.38	2.00	0.49	4.82
Length submerged (*L*_s_)	8.21	7.00	1.10	4.38	2.00	0.49	4.82
Equivalent diameter (*Z*_eq_):	**1.40**	**1.67**	**0.59**	**1.46**	**1.21**	**0.27**	**0.63**
Max diameter (*Z*):	1.59	1.65	0.44	1.43	0.58	0.49	0.64
Waterline length (*L*_w_)	3.23	4.12	0.85	2.50	2.00	0.42	2.49
Wetted surface area (m^2^):	19.87	18.68	1.74	18.09	3.00	0.38	4.92
Frontal surface area (submerged) (m^2^):	**1.53**	**2.20**	**0.27**	**1.68**	**1.14**	**0.06**	**0.31**
*C*_d_(hat)_wetted value:	0.00335	0.00385	0.01160	0.00516	0.01259	0.01440	0.00355
*C*_d_(hat)_frontal value:	**0.05903**	**0.04833**	**0.06529**	**0.04635**	**0.06268**	**0.07827**	**0.08163**
Muscle aerobic efficiency (*ε*_A_)	{**0.05**, 0.09}					**0.09**	**0.2**
Propulsive efficiency (*ε*_p_)	{0.16, 0.25, **0.3**, 0.33}						{0.43, **0.5**, 0.54}
Active to passive drag ratio (*λ*)	2						4
Mass (kg)	2643	3116	76.4	2981	226	25	496.1
Body fat% (*F*)	**5.18** 10.36	**5.18** 10.36	**5.18**	**9.75**	**22**	**1.6**	**9.2**
Fat mass (kg)	**136.9** 273.8	**161.4** 322.8	**4.0**	**290.6**	**49.7**	**0.4**	**45.6**

*Note:* The preferred parameters per taxon are shown in bold.

##### 2.1.5.2 Skin Friction Coefficient Formulae

The quantity *C*_f_ is the skin friction coefficient, a dimensionless number representing the viscous (frictional) contribution to the total drag coefficient *C*_d_. As Motani (2002) notes, *C*_f_ is not constant but scales with the Reynolds number (Re). Motani (2002) utilized a Reynolds number dependent function taken from Vogel (1996) to estimate *C*_f_, which is also what we use here. The Vogel (1996) equation for a flat plate in turbulent flow is:

(12) $$C_{f}=0.072Re^{-1/5}$$

There are numerous formulae that give the skin friction coefficient. The most suitable for 3‐dimensional objects such as ships and large (hull‐shaped) organisms in a turbulent flow is the so‐called ITTC57 formula (Gutarra and Rahman 2022):

(13) Cf−ITTC57=0.075logRe−22

This is the formula used by Gutarra et al. (2019) for the calculation of skin friction on ichthyosaurs for comparison with their CFD results. In the current study, we primarily use Equation (12) because it can be incorporated in the model much more easily (see below). However, we tested the *C*_f‐ITTC57_ formula (Equation 13) and found that it produced values not markedly different from those obtained by the *C*_f_ formula (Equation 12) (see the ENHYDROSS core dataset (Excel file) available on Zenodo: https://doi.org/10.5281/zenodo.18015597). Therefore, it was not necessary to use *C*_f‐ITTC57_ further for the purposes of this study.

#### 2.1.6 Circumventing the Circularity in the Drag Coefficient Calculation

As Gutarra and Rahman (2022) observed, an important caveat of the *U*_opt_ equation (Equation 6 and 7) is that it uses circular reasoning by using an estimate for *C*_d_ which itself depends on the relative velocity of flow. In particular, from the skin friction coefficient formulae (Equation 12 and 13), it is evident that *C*_d_ is dependent on the Reynolds number in some way or other. The Reynolds number is a well‐known dimensionless ratio of inertial to viscous forces that act upon an object in a fluid medium and is given by:

(14) Re=uLρμ

where *u* is the velocity of the fluid relative to the object, *L* is the characteristic dimension of the object (usually taken to be the total length in the case of an animal moving forward), *ρ* is the fluid density, and μ is the fluid's dynamic viscosity. Hence, *C*_d_ is dependent on velocity, making our optimal speed equation (Equations 6 and 7) circular and thus seemingly difficult or impossible to use at first sight. To fix this problem, we incorporated the skin friction coefficient formula (i.e., Equation 12) in the *U*_opt_ equation (Equation 6 or 7) and solve for *U*. This is relatively straightforward for Equation (12) but is much harder for Equation (13) for algebraic reasons. Therefore, we apply this step to just the first skin friction formula (Equation 12).

First, we write the drag coefficient Equation (10) by expanding the *C*_*f*_ term:

(15) $$C_{d_{\text{wetted}}}=\alpha \;\left(0.072\right){\left(\frac{\mathrm{L\rho}}{\mu }\right)}^{-1/5}\;\left[1+1.5{\left(\frac{Z}{L}\right)}^{1.5}+7{\left(\frac{Z}{L}\right)}^{3}\right]\;U^{-1/5}$$

And for Equation (11):

(16) $$C_{d_{\text{frontal}}}=\alpha \;\left(0.072\right){\left(\frac{\mathrm{L\rho}}{\mu }\right)}^{-1/5}\;\left[3\left(\frac{L}{Z}\right)+4.5{\left(\frac{Z}{L}\right)}^{0.5}+21{\left(\frac{Z}{L}\right)}^{2}\right]\;U^{-1/5}$$

where *α* is the multiplication factor to account for wave drag (or any other correction) for the *C*_d_ if necessary. We can write this expression for each *C*_d_ case (wetted or frontal area) as:

(17) $$C_{d}=\hat{C_{d}}U^{-1/5}$$

Substituting Equation (17) into Equation (4) we obtain COT_TOT_ as follows:

(18) $${\mathrm{COT}}_{\mathrm{TOT}}=\frac{M_{b}+M_{T}}{U}+\frac{1}{2}\frac{\rho \lambda S\hat{C_{d}}}{\varepsilon_{p}\varepsilon_{A}}U^{-1/5}U^{2}=\frac{M_{b}+M_{T}}{U}+\frac{1}{2}\frac{\rho \lambda S\hat{C_{d}}}{\varepsilon_{p}\varepsilon_{A}}U^{9/5}$$

By taking the derivative of this expression and setting COT_TOT_ = 0, we solve for *U* as we did previously and we once again obtain *U*_opt_, but this time modified so that it does not contain an extra *U* term on the right side of the equation, thus avoiding circularity:

(19) Uopt=109εpεAMb+MTρλSCd^514

or by choosing to omit *Μ*_Τ_ for reasons explained previously:

(20) Uopt=109εpεAMbρλSCd^514

Back‐substituting Equation (20) in every *U* term of Equation (18) we find the COT_min_:

(21) $${\mathrm{COT}}_{\min}=\frac{14}{9}\frac{\left(M_{b}+M_{T}\right)}{U_{\mathrm{opt}}}$$

or, by again zeroing the *M*_T_ term we obtain:

(22) $${\mathrm{COT}}_{\min}=\frac{14}{9}\frac{M_{b}}{U_{\mathrm{opt}}}$$

To see the exact steps taken to derive Equations (19) and (21) see Data S2.

#### 2.1.7 Calculating Swimming Distances and Times

So far, we have obtained two sets of equations: one for the optimal swimming speed that does not involve circularity with regards to the drag coefficient (Equations 19 and 20), and one for the minimum cost of transport corresponding to that speed (Equations 21 and 22). At this point, one can already see that our new model is no longer constrained to mammals (see Data S1), while at the same time we have been able to keep *U*_opt_ and COT_min_ as interdependent variables (as they should be). With these two equations, any group of animals can be modeled, provided that the parameters of the optimal speed equation are known or can be estimated. Up to this point, our model is already at an advantage compared to its predecessor.

The next steps are the same as those followed by Meijaard (2001) and Hertler et al. (2025, 2022), namely: (1) estimate the fat mass and its equivalent energy for each animal (see Section 2.4.7), so that (2) we can estimate the maximum swimming distances and their corresponding durations via Equations (1) and (2).

Remarkably, given Equations (1), (2), (8), (9), (21), and (22), we can see that unlike distance, the corresponding swimming time is completely unaffected by any changes in the parameters of *U*_opt_. The reason for this is that COT_min_ is inversely proportional to *U*_opt_, and swimming time is inversely proportional to COT_min_ and *U*_opt_, thereby canceling out the velocities. What this means is that the swimming time is independent of the swimming speed and in order to calculate it, one only needs the available energy stored in the body (or in our case just the fat mass) and *M*_b_ (or *M*_b_ + *M*_T_). This corresponds to 2/3 of the passive drifting time for Equations (8) and (9), and to 9/14 for Equations (21) and (22).

We have thus crafted a mechanistic model that, in theory, is capable of calculating the optimal swimming speed, COT_min_, and limit of dispersal via swimming in terms of both distance and its corresponding duration under conditions of null‐thermogenesis, such as those associated with near‐thermoneutral temperatures in water. This model effectively integrates both bioenergetics and hydrodynamics and is applicable at optimal swimming speeds. For brevity, we refer to it as *ENHYDROSS*, an acronym standing for *ENergetics and HYDRodynamics at Optimal Swimming Speed*.

### 2.2 Modeled Organisms

In order to validate our ENHYDROSS model, we tested it against data from documented cases of swimming in open water in extant animals. Based on several selection criteria (see Data S4), we modeled the following extant taxa: an Asian elephant ( *Elephas maximus* ) and a polar bear ( *Ursus maritimus* ), representing large mammalian endotherms; an ostrich ( *Struthio camelus* ) as an example of a large avian endotherm; and a saltwater crocodile (*Crocodilus porosus*) and an Aldabra giant tortoise ( *Aldabrachelys gigantea* ) as moderate to large reptilian ectotherms. None of our model animals have information for all three parameters of swimming speed, distance, and duration, and in one case (the ostrich), none of these are known empirically. Inclusion of the latter animal, however, was deemed worthwhile as an exploratory test with regard to a cursorial avian swimming via paddling, rather than necessarily a validating one.

Beyond the validation of our model, our study has the additional goal of applying it to test biogeographic hypotheses concerning Cretaceous dinosaurs (see Section 2.7.1). Hence, we have also selected nonavian dinosaur models using another set of criteria (see Data S4). These were a large hadrosaur and a small‐to‐medium‐sized titanosaur based on *Lambeosaurus lambei* and *Rapetosaurus krausei* respectively.

Because we lacked several physiological and biomechanical data, to fill the gaps in our knowledge of dinosaurs, we have made inferences based on living taxa. These include: metabolic rates and how these scale with body mass; how much fat mass is realistically expected for a dinosaur; and the values for the various parameters of the optimal swimming speed (*ε*_p_, *ε*_Α_ and *λ*).

### 2.3 Anatomical, Geometrical, and Buoyancy‐Related Data for the Models

One of us (DMH) provided the dimensions (total length, axial body maximum width and height), total body surface area, and mass data for the nonavian dinosaurs, the ostrich, the elephant, and the saltwater crocodile. In order to do so, a 3D model was constructed via the method of Henderson (1999) (for more details see Data S6) for each of these animals. The 3D models used to obtain these anatomical dimensions are available in the supplementary data repository on Zenodo (https://doi.org/10.5281/zenodo.18015597). For the polar bear and the tortoise, such data were acquired in a different way (see Data S6).

For the purposes of our study, we also require information related to buoyancy and hydrostatic stability. First, we need the waterline length (the length of the body that is in contact with the surface of water) of an object or animal because it is crucial for estimating (1) the hull speed (see Data S1: Equation S1) which itself is necessary for (2) estimating the Froude number and subsequently (3) the wave drag (see Section 2.4.5) as well as calculating the *U*_opt_ *sensu* Meijaard (2001) (Data S1). Secondly, we require the body wetted surface (*S*_wetted_) and the submerged frontal (*S*_frontal_) areas of the bodies. This is because, as we have explained (see Section 2.1.5), these areas dictate the choice between Equation (10) or (11) and are subsequently needed within Equations ((5), (6), (7)). Third, because we are dealing with surface swimming, we must be careful in how we define the geometrical input parameters used to derive hydrodynamic quantities, especially given the partial submergence of the bodies. As explained in Section 2.1.5, instead of using the total body length as the characteristic length (*L*) in Equations (10), (11), (15), and (16), as is usually done for fully submerged animals, we must use their respective submerged length (*L*_s_) to calculate a meaningful *C*_d_ for a partially submerged animal. Fourth, having performed a buoyancy test, we can also test for hydrostatic stability. In the context of our study, the latter type of test is a useful proxy for how well adapted an animal is for swimming locomotion and, by extension, trans‐oceanic dispersal. Hence, it was necessary for buoyancy and stability analyses to be made *sensu* Henderson (2018, 2014, 2004, 2003). For more information on buoyancy tests, the reader is referred to the Data S6.

### 2.4 Choice of Parameter Values

#### 2.4.1 Surface Area

Although we used both frontal and wetted surface areas to calculate the drag coefficient, we used the frontal surface area as the “preferred” surface type, acknowledging that our target animals do not uniformly fit the conditions that favor the use of either of the two surface areas (see Section 2.1.5). Some of our target animals are elongated but still poorly streamlined (dinosaurs); others are blunt and short (tortoise, bear, ostrich, and elephant), and the crocodile can be considered as elongated and streamlined compared to the other species. At the same time, the elongated animals operate at high Reynolds number values, whereas the blunt and short ones operate at both intermediate (tortoise, ostrich) and high (bear, elephant) values (For the calculated Reynolds numbers, see the ENHYDROSS core dataset available on Zenodo). Which of the two area types represents the most accurate choice remains subjective, and we therefore use both in order to determine the sensitivity of our results to these alternative assumptions. Values used for each surface area for every animal are shown in Table 1.

#### 2.4.2 Aerobic Efficiency

Aerobic efficiency (*ε*_A_) is the ratio of aerobic metabolic power allocated to locomotion that is converted into mechanical work (Figure 1). In mammalian muscle, *ε*_Α_ ranges from 0.24 to 0.33, and in vertebrates more broadly from 0.2 to 0.4 (Lovelace et al. 2020; Smith et al. 2005). However, with regard to the aerobic efficiency of the whole animal, *ε*_A_ cannot exceed 0.05 for swimming, paddling mammals whose swimming speeds are typically low (Fish 1996b). Thus, we adopted a conservative value of *ε*_A_ = 0.05 for all paddling animals (Table 1), except the tortoise. Typically, turtles have higher muscle efficiencies than other tetrapods because of their specialized muscle physiology among other things (Ewart et al. 2022 and references therein). To account for this difference and the lack of better data, we set the tortoise's *ε*_A_ = 0.09 (Table 1), which matches the green turtle's peak value at low swimming speeds (Fish 1984 citing Prange 1976). Finally, given that crocodilians swim primarily by using lateral tail undulations, the value for the crocodile was retained at 0.2 (Table 1), following the example of Villamil et al. (2016) and Massare (1988) for other marine reptiles. For a more detailed discussion see Data S3.

#### 2.4.3 Propulsive Efficiency

Propulsive efficiency (*ε*_p_) is the ratio of useful thrust power to the total mechanical power generated during swimming (Fish 2020, 1996b) (Figure 1). For the paddling animals (dinosaurs, ostrich, elephant, tortoise and polar bear), we used *ε*_p_ = 0.3, with sensitivity tests spanning the plausible range 0.16–0.33 (Table 1) of values used in the literature for a variety of animals (Blanco 2023; Fish 2000, 1996b, 1992, 1984a; Webb and Blake 1985). Undulatory swimmers such as crocodiles show higher *ε*_p_ than paddlers, with values 0.43 < *ε*_p_ < 0.54 for a typical undulating fish, such as the American eel (Fish, Rybczynski, et al. 2021). Thus, for the crocodile, we applied a rounded mean of 0.5 from this range (Table 1), which agrees well with the mean value of 0.51 used by others (Villamil et al. 2016 and references therein). Further discussion and rationale for the adopted values, as well as potential differences between bipedal and quadrupedal paddling, are provided in Data S3.

#### 2.4.4 The λ Parameter

The λ parameter is a dimensionless quantity whose definition varies across studies. It has been interpreted either as a general correction factor for cumulative model error (e.g., Motani 2002) or as the ratio of active to passive drag (Hind and Gurney 1997). Because *λ* depends on swimming mode which remains unknown for all taxa examined here, we selected values from the literature. In human swimming studies, where *λ* represents the ratio of active to passive drag, reported values range from 1.5 and 2.5 (Zamparo et al. 2020 and references therein). We therefore used the mean value of *λ* = 2 for all paddling swimmers (Table 1). For undulatory swimmers such as crocodilians, direct estimates are lacking. However, drag produced during undulation at maximum speed in *Spinosaurus* was reported to be 3–5 times stationary drag (Sereno et al. 2022 and references therein). We assumed similar ratios apply to crocodiles and consequently used an intermediate value of *λ* = 4 for the crocodile (Table 1), likely a conservative (high) estimate given that our analyses target optimal rather than maximal swimming speeds. Additional comments are presented in the Data S3.

#### 2.4.5 Wave Resistance

Objects or animals moving at the water surface generate waves as they travel and the energy required to produce these waves manifests as wave resistance (also known as wave drag) (Figure 1). This form of drag is unique to surface swimming and decreases as depth increases. According to Hertel (1966, 227) wave drag can be up to 3–5 times larger than the total drag experienced by a body at a depth of three times its diameter. However, Hertel's (1966) experimental conclusion says nothing about the cruising speed, which also determines the wave resistance. This is where the Froude number (Fr) becomes essential.

The Froude number (Fr), a dimensionless ratio of inertial to gravitational forces widely used to compare surface‐swimming performance among different bodies, is given by:

(23) $$\mathrm{Fr}=\frac{u}{\sqrt{gL_{w}}}=\frac{\text{inertia forces}}{\text{gravitational forces}}$$

where *u* is the velocity of the object relative to the fluid, *L*_w_ is the waterline length of the body, and g is the gravitational acceleration 9.81 m/s^2^. Displacement hulls, including swimming animals, experience maximal wave drag at intermediate Froude numbers (Fr = 0.4–0.45), while lower and higher values reduce wave drag; the former by producing less wave energy and the latter as hydrodynamic lift increases with speed, transitioning the body from a displacement‐dominated regime to a lift‐dominated hydroplane mode (Aigeldinger and Fish 1995; Gough et al. 2015).

To assess wave drag, we can use as an approximation, a plot of wave drag vs. Froude numbers of a hull taken from Hoerner (1965, 11–12, fig. 10), where increments of 0.01 in *C*_d_ correspond to integer multiples of total underwater drag, herein designated as *α* (i.e., at Fr = 0.3 ➔*C*_d_ = 0.01➔*α* = 1, at Fr = 0.4➔ *C*_d_ = 0.02➔*α* = 2, etc.) (Frank Fish, pers. communication; see also Fish and Baudinette 1999; Figure 6). By interpolating across the full curve of Fr vs. *C*_d_, we calculated the corresponding multiplier α and evaluated Fr for each taxon across a range of speeds overlapping its predicted optimal swimming speed. In all cases, values fell well below thresholds associated with significant wave drag. Consequently, we set the wave‐drag multiplier *α* = 1 (i.e., no added wave resistance) in all calculations (Table 1; see also the ENHYDROSS core dataset available on Zenodo).

#### 2.4.6 Metabolic Data

##### 2.4.6.1 Extant Taxa

For our extant species, we used published allometric equations from the literature (Table 2). For our elephant model, we used the allometric equation for the basal metabolic rate (BMR = 18.16 M^0.58^) from Dunkin et al. (2013). The metabolic rate for a 92.4 kg ostrich was estimated empirically by Withers (1983) as 0.113 mL O_2_ g^−1^·h^−1^, from which McNab (2009) estimated its BMR as 209.66 kJ·h^−1^. Since 1 kJ·h^−1^ = 0.278 W approximately, multiplying the BMR by that number gives it as power equal to 58.24 W. Because our ostrich model has a different mass (76.4 kg) from those in the empirical studies, we chose to use the Struthioniformes allometric formula (BMR = 2.45 M^0.705^) from McNab (2009). Seymour (2013) derived an allometric relationship for the Standard Metabolic Rate (SMR = 0.101 M^0.829^ in ml O_2_/min) for *Crocodylus porosus*, covering several orders of magnitude of body mass (0.19–389 kg). Note that SMR is more appropriate for ectotherms than BMR, although the two terms are sometimes used synonymously in the literature and for our purposes here should be taken to mean the same thing. Our *Crocodylus porosus* has a body mass (496.1 kg) greater than the highest value used to derive this SMR equation, meaning that errors resulting from extrapolation may accrue. Based on the lower (0.803) and upper (0.855) limits of the 95% confidence intervals of the estimated exponent (0.829) in the allometric equation (SMR = 0.101 M^0.829^) of Seymour (2013), the corresponding metabolic power could range between 49.4 W and 68.2 W. To limit the complexity of our analyses, only the exponent value of 0.829 was utilized in this study. For the Aldabra tortoise, we used the allometric equation (BMR = 0.25404 M^0.82^) from Hughes et al. (1971) for minimum V̇O_2_ (=45.5M^0.82^ in ml/h), assuming this to be equivalent to BMR (or SMR depending on how one defines it). Finally, for the polar bear, we used two equations from Pagano et al. (2018) which differ with regard to posture. First, we used the post‐absorptive average resting metabolic rate (RMR) for adult female polar bears (*n* = 5) of 0.27 ± 0.01 mL O_2_ g^−1^·h^−1^ converted to Watts, a value which we term the “resting BMR.” Then we used the linear relation for walking polar bears (*n* = 35), V̇O_2_ = 0.44 + 0.12 × speed, but with speed set to zero, and converting it to Watts; this value is hereafter referred to as the “standing BMR.” It is unknown which of these values would be more appropriate for a swimming bear, but the range most likely brackets reality. Data for a resting subadult polar bear in water indicate a BMR of 0.39 mL O_2_ g^−1^·h^−1^ on average, whereas for swimming and diving the mean value was 0.59 mL O_2_ g^−1^·h^−1^ Pagano et al. (2019), which is higher than either of the values estimated for adults. We chose not to use either of these subadult values because our polar bear is modeled after an adult female, and because of the possible reasons for the observed differences between the subadult and adults listed by Pagano et al. (2019).

**TABLE 2:** Allometric equations for scaling BMR with body mass for a variety of higher clades, including endothermic (birds and mammals), poikilothermic ectotherms (reptiles), and ectotherms such as tunas and turtles which exhibit regional endothermy/heterothermy in their bodies to produce higher metabolic output and maintain elevated levels of activity compared to other ectotherms.

References	Type of BMR	Equation after conversion to Watts and simplified where appropriate
Motani (2002)	Reptiles (Upper)	0.14*(M^0.802)*10^(0.43*(1.01 + 1.04*(10^(−2))*(log(M) + 0.585)^2)^0.5)
	Mammals_Eq.8 (Upper)	2.93*(M^0.723)*10^(0.459*(1.00 + 1.94*(10^(−3))*(log(M) + 0.500)^2)^0.5)
	Mammals_Eq.8 (Lower)	2.93*(M^0.723)*10^−(0.459*(1.00 + 1.94*(10^(−3))*(log(M) + 0.500)^2)^0.5)
	Tunas & Turtles (Upper)	1.36*(M^0.760)*10^(0.358*(1.06 + 5.60*(10^(−2))*(log(M)−1.85)^2)^0.5)
	Tunas & Turtles (Lower)	1.36*(M^0.760)*10^−(0.358*(1.06 + 5.60*10^−2^*(log(M)−1.85)^2)^0.5)
Seymour (2013) and references therein	Saltwater Crocodile	0.338*M^0.829^
	Mammals	2.965*M^0.676^
White et al. (2006)	Reptiles (Q10)	0.630*M^0.768^
	Reptiles (UTD)	0.607*M^0.769^
	Birds (Q10)	2.756*M^0.644^
	Birds (UTD)	2.769*M^0.644^
	Mammals (Q10)	2.806*M^0.678^
	Mammals (UTD)	2.819*M^0.678^
McNab (2009)	Birds	3.669*M^0.652^
	Birds(nonpasserines)(1)	3.675*M^0.721^
	Birds(nonpasserines)(2)	3.627*M^0.724^
	Struthioniformes	2.450*M^0.705^
	Ratites (***)	2.611*M^0.705^
	Palaeognathae (****)	2.693*M^0.705^
Withers (1983)	Ratites (**)	2.172*M^0.73^
McNab (2008)	Mammals	2.853*M^0.721^
	Eutherians	2.954*M^0.724^
	Marsupials	2.275*M^0.724^
	Monotremes	1.359*M^0.724^
	Artiodactyla	4.417*M^0.707^
Dunham et al. (1989)	Squamates	((0.202*10^(0.038*T_c_−1.771)) + M^0.82)/3.6
Lovelace et al. (2020) and references therein	Monotremes	1.373*M^0.724^
	Marsupials(1)	2.288*M^0.724^
	Marsupials(2)	2.542*M^0.737^
Lee (2015)	*Alamosaurus*	1.88*M^2/3^
	*Maiasaura*	1.77*M^2/3^
Dunkin et al. (2013)	Elephants	18.16*M^0.58^
Pontzer et al. (2009) and references therein (+++)	Mammals	3.4*M^0.75^
	Birds (nonpasserines)	3.79*M^0.72^
	Reptiles	0.69*M^0.82^
Pagano et al. (2018)	Polar Bear_resting	(20.1/3600*1000)*0.27*M
	Polar Bear_standing	(20.1/3600*1000)*(0.44 + 0.12*U)*M [for U = 0]
Hughes et al. (1971)	Aldabra giant turtle_(BMR)	0.254*M^0.82^

*Note:* *M* and *T*_c_ denote body mass in kg and core temperature in °C., respectively. The symbol (**) indicates that the formula is derived from a sample of ostriches and kiwis (Apterygiformes). The symbol (***) indicates the usage of the mean (*O*) coefficient from Table 1 in McNab (2009) of the clades (Apterygiformes, Casuariiformes, Struthioniformes). The symbol (****) indicates the usage of the mean (*O*) coefficient from Table 1 McNab (2009) of the clades (Apterygiformes, Casuariiformes, Struthioniformes, Tinamiformes). The symbol (+++) indicates that BMR and RMR are used interchangeably within this paper. The T_c_ (body core temperature) in the squamate metabolism equation by Dunham et al. (1989) is 36°C for both our modeled extinct dinosaurs.

##### 2.4.6.2 Dinosaur Metabolism

It is beyond the scope of the current study to argue for or against particular metabolic regimes for dinosaurs or to attempt to resolve the debate over the metabolic allometry exponents in general. Instead, we have opted to include a range of BMRs/SMRs obtained from a list of published allometric scaling equations for our modeled hadrosaur and sauropod (Table 2). This approach is similar to that taken by other relevant studies of extinct taxa (Hartman et al. 2022; Lovelace et al. 2020), and it enables us to assess the sensitivity of our results to choice of metabolic rate. We provide a more detailed discussion about the subject in the Data S7.

#### 2.4.7 Fat Mass

Fat percentages for each extant taxon were obtained from the literature (Table 1), prioritizing taxonomically specific allometric equations or empirical data over broader‐order estimates to better capture interspecific variation in adiposity. For the elephant, we took the mean (9.75%) of 38 zoo elephants of various ages and both sexes (Chusyd et al. 2021: Table 1). Zoo animals might not be representative of the typical fat storage in the wild because of the more regular availability of food in captivity. On the other hand, the fat mass percentage of total body mass in the zoo elephants ranged from 25% to a mere 2%, demonstrating the plasticity of this physiological trait. For this reason, this was deemed a suitable sample size. For the ostrich, fat mass was estimated using the mean % (5.18%) of caul and abdominal fat, based on 14 individuals (Morris et al. 1995: Table 2). For the polar bear, we used the post‐swim mass loss recorded for the female bear which swam 687 km in 9 days (Durner et al. 2011), assuming that the 22% reduction in body mass (226 kg before and 159 kg after) was all attributable to fat loss. For the Aldabra tortoise, we used the median value (1.6%) from the range of fat‐to‐body volume ratios (0.07%–2.5%) measured in another testudinid species ( *Gopherus agassizii* ) (Walden et al. 2022), and assumed it to be equivalent to the fat mass‐to‐body mass ratio. Based on this, we calculated the fat mass for our modeled 25 kg tortoise as follows: Fat mass = Body mass (kg) × Fat‐to‐body mass ratio (%) = 25 kg × 0.016 = 0.4 kg. For *C. porosus* , we could not find a publication explicitly recording fat mass, so we used information from the mean values of seven slaughtered Nile crocodiles ( *C. niloticus* ) (Hoffman et al. 2000), a close relative of similar size. Specifically, total fat mass % (9.2%) was estimated by summing 2.3% of live weight for gut fat (Hoffman et al. 2000: Table 1) plus the 6.9% of live weight for the fat in torso, legs, neck and tail (Hoffman et al. 2000: Table 2). For the two extinct dinosaur models, we used two fat mass values to reflect plausible variation and avoid relying on a single estimate. For a conservative fat mass percentage, we used the same value as the ostrich (5.18%). The reasoning here is that neither of our dinosaurs is likely to be more athletic than an ostrich; therefore, this value is considered a minimum for such large herbivorous animals. For an upper limit, we doubled the minimum fat percentage to 10.36%, which is close to the values of our elephant and crocodile.

#### 2.4.8 Water Privation Tolerance Limits

Finally, we wanted to evaluate the extent to which our estimated swimming distances and durations differ from reality. In order to do this, we included water privation limits. How long an animal can last without water depends on a variety of factors, including environmental temperature and humidity, which can affect the rate of evaporation from an animal's body. Water loss from the body is also influenced by physiological adaptations which, other things being equal, might benefit animals adapted to xeric conditions if they found themselves required to swim long distances (Mazza et al. 2019 and references therein). Data on water deprivation limits are scarce, and even when available they are often unreliable as true limits: in some cases, they represent the shortest known duration without water, and in others they may or may not include water gained from ingestion of food. Consequently, water deprivation limits listed in the literature show few discernible patterns (Data S7: Table S7.1). Furthermore, as noted previously (Longrich et al. 2021; Mazza et al. 2019), animals can take advantage of their metabolic water, a byproduct of their fat oxidation. Each gram of fat amounts to 1.07 g of H_2_O (Mazza et al. 2019 and references therein). Because we are currently not aware of any method that can calculate how long an animal can last without water, we selected an arbitrary value, 14 days, as the maximum duration threshold without access to water; this duration is within the observed values for animals such as a camel (17 days) (Schmidt‐Nielsen 1959) and a dog (11–20 days) (Mazza et al. 2019 and references therein). We also calculated how a shorter, 7‐day threshold affects the results (see Section 2.5 and Data S7: Figure S7.5).

### 2.5 Sensitivity Tests

To test the effects of the various parameters on the results of the ENHYDROSS model, we performed seven analyses per animal, each varying one or more of the parameters *ε*_p_, *ε*_Α_, *C*_d_, fat mass (*F*), and a BMR × 2.3 enhancement factor, relative to a predefined combination of preferred parameter values per animal (N.B. not all animals were subjected to the same type of tests). The BMR × 2.3 factor was used as a proxy for elevated energetic costs associated with additional thermogenesis in water (see Data S5). A summary of these sensitivity tests is presented in Table 3. For *ε*_p_, we took the values mentioned previously (0.16, 0.25, 0.3, 0.33) for the paddlers, whereas for the crocodile we tested the values 0.43, 0.5, and 0.54. For all paddlers we considered *ε*_p_ = 0.3 as the preferred parameter value, whereas for the crocodile we used 0.5 instead. For the *ε*_Α_, the values 0.05 and 0.09 were tested for the mammals, the ostrich and the two nonavian dinosaurs; by contrast, the default values of 0.09 and 0.2 for the tortoise and crocodile, respectively, were kept constant in all tests because we had no information from the literature about a plausible range or alternative values. For all animals, we considered the frontal area to be our preferred choice in the *C*_d_ calculation, and thus used this when varying all other parameters. An analysis where all other parameters were set to their preferred values was carried out using the *C*_d_ estimated with the wetted surface areas. The *λ* parameter was left constant for all animal models. Varying *λ*, while simultaneously varying the other parameters, would further complicate interpretation of the sensitivity test outputs, with little gain in insight (see Data S3: Table S3.1). When varying all four of the hydrodynamic parameters (*ε*_p_, *ε*_A_, *λ*, and *C*_d_) of the *U*_opt_ equation based on the possible values obtained from the literature, there are 48 possible combinations (twice the number of cells in Table S3.1). Multiplying this by four to account for the two different fat masses and the presence or absence of the 2.3 × BMR enhancement (see Data S5) for our nonavian dinosaur models would result in a theoretical maximum of 192 different combinations per dinosaur. We did not run all of these combinations, and instead focused on a representative subset (Tables 3 and S3.1), that seem more realistic for reasons explained previously (Sections 2.4.1, 2.4.4 and Data S3). Finally, a total of 32 different BMR power laws, sourced from nine publications, were applied to each of our two nonavian dinosaurs (Table 2). In terms of water privation limits, all the above analyses were performed without any threshold at first and then with a 14‐day water privation time limit. An additional analysis with a 7‐day limit was also performed (Data S7).

**TABLE 3:** Sensitivity tests performed for the ENHYDROSS model with parameters in bold being the “preferred” ones for a given taxon.

Sensitivity test name	*Lambeosaurus lambei + Rapetosaurus krausei*	*Struthio camelus* + *Elephas maximus*	*Ursus maritimus*	*Aldabrachelys gigantea*	*Crocodylus porosus*
*ε*A*εp/*λ* = 6.3e‐03	ε_p_ = 0.25				*ε*_p_ = 0.43
*ε*A*εp/*λ* = 7.5e‐03 (preferred parameters)	***ε***_**Α**_ **= 0.05**	***ε***_**Α**_ **= 0.05**		***ε***_**Α**_ **= 0.09**	***ε***_**Α**_ **= 0.2**
	***ε***_**p**_ **= 0.3**	***ε***_**p**_ **= 0.3**		***ε***_**p**_ **= 0.3**	***ε***_**p**_ **= 0.5**
	***F* = 5.18% Body Mass**	***F* = default**		***F* = default**	***F* = default**
	***C***_**d**_ **_Frontal_Area**	***C***_**d**_ **_Frontal_Area**		***C***_**d**_ **_Frontal_Area**	***C***_**d**_ **_Frontal_Area**
*ε*A*εp/*λ* = 8.3e‐04	*ε*_p_ = 0.33				*ε*_p_ = 0.54
*ε*A*εp/*λ* = 7.2e‐03	*ε*_Α_ = 0.09			*ε*_p_ = 0.16	_
	*ε*_p_ = 0.16				
*ε*A*εp/*λ* = 7.5e‐03_F10	*F* = 10.36% Body Mass	_			
*ε*A*εp/*λ* = 7.5e‐03_Cd_Wetted_Area	*C*_d__Wetted_Area				
*ε*A*εp/*λ* = 7.5e‐03_BMR × 2.3	BMR × 2.3		_		

*Note:* All these tests were performed (1) without any time limit, (2) with a 14‐day and (3) with a 7‐day water privation time limit. F is fat mass. The significance of bold values indicated to parameters.

### 2.6 Passive Drifting Distances and Times as Upper Limits for Model Validation Purposes

The purpose of our ENHYDROSS model is primarily the estimation of swimming distances and durations and, like any other model, it is only as good as validation data shows it to be. Validation for our model's results comes from three variables: *U*_opt_, max swimming distance and corresponding time (recorded data collected from extant taxa lack information about COT_min_). With regard to swimming distance and duration, in particular, model estimates that exceed the maximum observed empirical values do not necessarily invalidate the modeling approach. This is because the empirical observations of animals in the wild do not necessarily represent maximum values because the animal might have been capable of swimming further after the point at which it was observed. In other words, empirical observations on swimming/floating distance and duration provide a lower limit on what is feasible, not an upper limit. By the same logic, cases where modeling underestimates maximum swimming/floating distance and duration, relative to empirical observations, are almost certainly problematic and would mean that the particular model used is probably wrong in some way or another. The only case where model underestimates would be compatible with a valid modeling approach would be when one of the initial assumptions is itself invalidated. In particular, neither Meijaard's (2001) model, nor ENHYDROSS, take ocean currents into account. Ocean currents can increase dispersal distances beyond those predicted by bioenergetic models of active swimming. In addition, passive drifting can prolong the maximum survival time of an organism in water, because of conservation of energy that would otherwise be spent on locomotion. This is a problem when it comes to validation, since an underestimate of duration could be attributed to either the organism's inactivity due to a period of passive drifting which prolonged its swimming duration, or alternatively to the model's inadequacy. To overcome this problem in a simple way, we have estimated the upper limit of duration an animal can have when it remains inactive in water, that is, passive drifting/floating. This was done using a special case of Equations (1) and (2) as described in Section 2.1.2. We also calculated passive drifting times using the 2.3 × BMR enhancement, as opposed to just the BMR, for a preliminary assessment on an average elevated cost that the endothermic animals (other than the polar bear) would experience while drifting passively but burning extra energy to stay warm (i.e., an *M*_T_ > 0). We then proceeded to calculate how far an animal can be transported solely by the action of ocean currents while it remains afloat, but inactive. Considering the range of modern oceanic surface currents is 0.25–2.5 m/s, with an average of 0.5 m/s (Mazza et al. 2019 and references therein), we selected a mild (0.25 m/s) and an intermediate strength (0.5 m/s) current for conservative estimates. In the specific case of the tortoise, however, since Gerlach et al. (2006) reported a range of 1–3 knots (~0.5–1.5 m/s) for possible current speeds, we instead tested an intermediate (0.5 m/s) and a strong (1 m/s) current. In this way we have created both a lower limit using the results from the ENHYDROSS model (or Meijaard's (2001) model for that matter), which simulates actively swimming animals without any ocean current action, as well as an upper limit of distance and duration, simulating a passive drifter governed by ocean currents (both in thermoneutral conditions, and not, where possible). In this case, passive drifting time is indeed a theoretical upper limit, but the same cannot be said for drifting distances necessarily, since: (1) higher current velocities can exist, for example, in instances of storm outbreaks, where an animal can be pushed further away within its maximum survival time; and (2) when active swimming is combined with the action of ocean currents, distances can indeed increase. However, for practical purposes and given our empirical data, we regard our drifting distances as sufficient as an upper limit. Together, these form a bracketed range in which any observation must fall in‐between, in order for the model to be considered valid. Furthermore, when the observations lie close to the lower limit, we could argue that the presence of ocean currents is limited, whereas the opposite is true if the results lie closer to the drifting limits. This can inform us as to which empirical instances were probably influenced by ocean currents, which in turn can point to departures from our model estimates that can be attributed to the effects of such currents, and thus eliminating part of the uncertainty with regards to the model's validation. Finally, because the way to calculate the passive drifting time (see Section 2.1.2) is independent of and more fundamental than either model, it is also much more robust and accurate in terms of an energetic limit to dispersal, so its role as an upper limit cannot be questioned (at least when it comes to thermoneutral conditions).

In short, a validated model should generally predict dispersal distances that equal or exceed empirical observations. Model underestimates of swimming distance and duration only fail to undermine the validity of a model when there is evidence for a strong impact of ocean currents, or the animal drifted passively (=inactive) for a substantial portion of its journey.

### 2.7 Applying the Model in a Paleogeographic Setting

#### 2.7.1 Hypotheses for Trans‐Tethyan Dispersals

**FIGURE 2:**
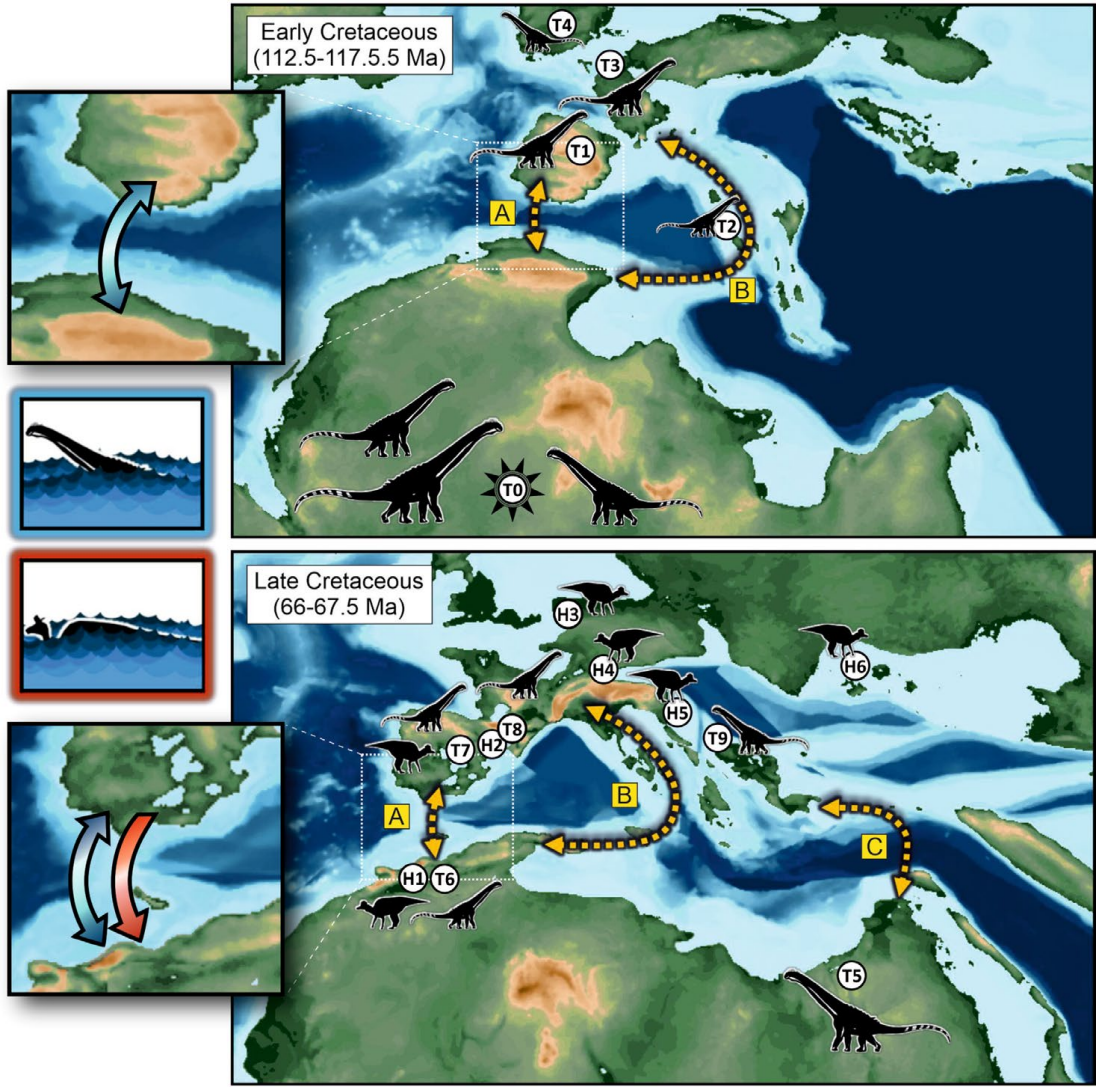
Paleogeographic maps of the European and African coastlines along the western Tethys Ocean during the late Early Cretaceous (117.5–112.5 Ma; top) and latest Cretaceous (67.5–66 Ma; bottom), adapted from Scotese et al. (2024). Note that although the maps represent discrete time intervals, the plotted dinosaur clades span broader temporal ranges, specifically from the Barremian to Cenomanian for Titanosauria and from the Campanian to Maastrichtian for Hadrosauridae. Dashed yellow arrows indicate proposed faunal exchange routes between Europe and Africa: (A) Alboran route, (B) Adria/Apulian route, and (C) proto‐Levant route. Insets highlight the Alboran route, emphasizing the suggested dispersal directions of Hadrosauridae (red arrow) and Titanosauria (blue arrows). Titanosauria: (T0) Western Gondwana titanosaurs; (T1) Iberia: Titanosauria indet. (Mocho et al. 2019); (T2) Italy: Titanosauria indet. (Dal Sasso et al. 2016); (T3) France: *Normanniasaurus genceyi* (Le Loeuff et al. 2013); (T4) UK: Titanosauria indet. (Upchurch et al. 2011); (T5) Egypt: *Aegyptosaurus baharijensis* (Stromer 1932), *Igai semkhu* (Gorscak et al. 2023), *Mansourasaurus shahinae* (Sallam et al. 2018), *Paralititan stromeri* (Smith et al. 2001); (T6) Morocco: Titanosauria indet. (Pereda‐Suberbiola et al. 2004); (T7) Iberia: *Abditosaurus kuehnei* (Vila et al. 2022), *Atsinganosaurus velauciensis* (Díez Díaz et al. 2018), *Lirainosaurus astibiae* (Sanz et al. 1999), *Lohuecotitan pandafilandi* (Díez Díaz et al. 2016), *Qunkasaura pintiquiniestra* (Mocho et al. 2024); (T8) France: *Ampelosaurus atacis* (Le Loeuff 1995), *Garrigatitan meridionalis* (Díez Díaz et al. 2021); (T9) Romania: *Magyarosaurus dacus*, *Paludititan nalatzensis*, *Petrustitan hungaricus*, *Uriash kadici* (Díez Díaz et al. 2025). Hadrosauridae: (H1) Morocco: *Ajnabia odysseus* (Longrich et al. 2021), *Minqaria bata*, Hadrosauridae indet. (Sidi Daoui), Hadrosauridae indet. (Mrah Lahrach) (Longrich et al. 2024); (H2) France and Spain: *Adynomosaurus arcanus* (Prieto‐Márquez et al. 2019), *Arenysaurus ardeveoli* (Pereda‐Suberbiola et al. 2009), *Blasisaurus canudoi* (Cruzado‐Caballero et al. 2010), *Canardia garonnensis*, Hadrosauridae indet. (Serrat del Rostiar) (Prieto‐Márquez et al. 2013), *Koutalisaurus kohlerorum* (Prieto‐Márquez et al. 2006), *Pararhabdodon isonensis*, Hadrosauridae indet. (Basturs Poble) (Fondevilla et al. 2018); (H3) The Netherlands and Belgium: *Orthomerus dolloi* (Madzia et al. 2020), Hadrosauridae indet. (Dalla Vecchia 2006); (H4) Germany: Hadrosauridae indet. (Dalla Vecchia 2006); (H5) Slovenia: Hadrosauridae indet. (Dalla Vecchia 2006); (H6) Ukraine: Hadrosauridae indet. (Dalla Vecchia 2006).

To explain their distributions and evolutionary relationships, a number of authors have hypothesized that several dinosaur lineages, including spinosaurid, carcharodontosaurid, and abelisauroid theropods, rebbachisaurid, and titanosaurian sauropods, and lambeosaurine hadrosaurs, as well as other taxa (e.g., neobatrachian frogs, notosuchian crocodyliforms, bothremydid turtles, madtsoiid snakes, lepisosteiform, characid and mawsoniid fishes) could have crossed the Tethys Ocean between Africa and Europe at sometime during the Cretaceous (e.g., Bosellini 2002; Buffetaut 1989; Canudo et al. 2009; Csiki‐Sava et al. 2015; Dal Sasso et al. 2016; Dalla Vecchia 2002, 1998, 1994; Díez Díaz et al. 2025; Galton 1977; Gheerbrant and Rage 2006; Longrich et al. 2024, 2021; Mezga et al. 2006; Nicosia et al. 2007; Ősi et al. 2010; Petti et al. 2020; Randazzo et al. 2021; Sacchi et al. 2009; Sereno et al. 1994; Upchurch In press; Zarcone et al. 2010). By reviewing these studies, two nonmutually exclusive hypotheses regarding the timing of trans‐Tethyan dispersals of titanosaurian sauropods emerge (Díez Díaz et al. 2025; Upchurch In press). One hypothesis suggests that Gondwanan lineages of titanosaurs dispersed via Africa into Europe during the late Early Cretaceous, presumably sometime between the Aptian and Cenomanian (Figure 2). Another proposes that one or more dispersals between Europe and Africa, potentially in both directions, took place during the latest Cretaceous (Figure 2). Regarding lambeosaurine hadrosaurs, Longrich et al. (2024, 2021) suggested that trans‐oceanic dispersal between Europe and Africa occurred during the latest Cretaceous (Campanian–Maastrichtian) (Figure 2).

Trans‐Tethyan dispersals during the Cretaceous could have taken place via three routes: (1) the Apulia/Adria route, which involved a series of islands or landspans (*sensu* Iturralde‐Vinent and Maphee 1999; Jacobs et al. 2011) formed by periodically emergent carbonate platforms that occurred between present‐day Italy+Croatia in the north and Algeria+Tunisia+Libya in the south (Bosellini 2002; Canudo et al. 2009; Dal Sasso et al. 2016; Dalla Vecchia 2002, 1998, 1994; Díez Díaz et al. 2025; Gheerbrant and Rage 2006; Mezga et al. 2006; Nicosia et al. 2007; Ősi et al. 2010; Petti et al. 2020; Randazzo et al. 2021; Sacchi et al. 2009; Upchurch In press; Zarcone et al. 2010) that could have facilitated geodispersal (*sensu* Lieberman 2000); (2) the Albora route, between southern Iberia and present‐day Morocco, which would have been a largely oceanic route, with perhaps a few stepping stone islands, and thus required long‐distance trans‐oceanic dispersal (Buffetaut 1989; Canudo et al. 2009; Galton 1977; Gheerbrant and Rage 2006; Longrich et al. 2021; Ősi et al. 2010; Sereno et al. 1994); and (3) the ‘proto‐Levant’ route, between Egypt/the Arabian Peninsula and the Taurus island and/or other nearby islands of present‐day Turkey, which would have also been a largely oceanic route as a stepping stone to the central European Tethys archipelago (Buffetaut et al. 2015; Dalla Vecchia 2002; Le Loeuff 1991; Ősi et al. 2010).

Having estimated the trans‐oceanic dispersal abilities of a hadrosaur and titanosaur using our ENHYDROSS approach, we applied these results to a palaeobiogeographic case study. Specifically, we examine the plausibility of one potential trans‐oceanic route between Europe and Africa during the Cretaceous: the Albora route. This route was chosen for its relative simplicity in terms of obtaining meaningful distance estimates for a trans‐oceanic journey. In contrast, the other two routes involve complex island networks with varying distance relationships across time slices, making them more challenging to analyze. Finally, the feasibility of such trans‐oceanic dispersal during the Cretaceous should be considered in the context of the unusually warm climatic conditions that characterized much of this interval, which are relevant to the physiological assumptions of our model (see Data S7).

#### 2.7.2 Estimating Shortest Trans‐Oceanic Dispersal Distances

In order to estimate the shortest shore‐to‐shore distances between southern Iberia and northwestern Africa during the Cretaceous, we used GPlates (Müller et al. 2018). For the paleogeographic reconstructions, we used those of Scotese et al. (2024), which present maps in 5 million year time slices. Given the relatively coarse spatial resolution of the coastlines in these paleogeographic reconstructions, we have chosen to estimate the shortest shore‐to‐shore distance (approximately within < 5 km error margins) between the two mentioned regions using the “Measure” tool from the GPlates tool palette, which measures the shortest geodesic distance between two selected points on the globe. The distances we have measured begin at 152.5 Ma (Late Jurassic) and form time slices of a 5 myr duration up to 62.5 Ma, although intervals more recent than 66 Ma (K/Pg boundary) are not relevant to our study because we are interested in nonavian dinosaurs. The palaeocoordinates for the selected paired points that define each distance segment can be accessed in the ENHYDROSS core dataset available on Zenodo. These Ibero‐Armorica to Africa distances during the Cretaceous serve as the limits, that is, the estimated minimum swimming distances across the Tethys Ocean that are required for a successful crossing, with which we compare and test our estimates of maximum feasible swimming distances derived for our dinosaur models. Because some time slices of Scotese et al. (2024) included islands, we measured two sets of distances: one without, and one with, islands present *en route*. This is important for investigating how the presence and location of stepping stone islands impact our results. According to the reconstructions of Scotese et al. (2024), islands were present in the region of the Albora dispersal route during the intervals of 147.5–142.5 Ma and 77.5–66 Ma, as far as the spatial resolution allows us to discern. Figure S7.3 (Data S7) shows the time slices, the oceanic gap distances measured for each, and the method used to determine distances in cases where islands were present.

However, it is important to acknowledge that paleogeographic reconstructions vary, both between models and even across versions of the same model. For example, earlier reconstructions by the same author (C. Scotese 2016; C. R. Scotese 2021) differ in island timing and configuration. Moreover, Buffan et al. (2023) have raised concerns that such variation can impact paleobiogeographic conclusions. To assess this potential variability, we also measured distances using another open‐access model: Cao et al. (2017). While we chose to base our main analysis on the most recent and highest‐resolution maps Scotese et al. (2024), these comparisons illustrate how the estimated minimum crossing distances can shift significantly depending on the chosen paleogeographic model. These model‐dependent differences are plotted in Figure S7.3 (Data S7).

### 2.8 Quantifying the Uncertainty of Dinosaurian BMR

Because we have a discrete set of plausible BMRs for non‐avian dinosaurs (see Section 2.4.6.2, Table 2), dispersal feasibility cannot be expressed confidently as a single predicted swimming distance. To accommodate this uncertainty, we reformulated the problem from estimating swimming distance for a given BMR to identifying the range of metabolic rates that would permit a successful crossing of a given marine gap.

To achieve this, we first generated curves describing the underlying continuous relationship between metabolic rate and maximum swimming distance, both with and without a time limit, enabling general trends and patterns to be identified. Note that throughout this section, “BMR” is used in the broader sense of metabolic heat production (*M*_b_ + *M*_T_), because the plotted relationship cannot distinguish basal metabolism (*M*_b_) from any additional thermogenic cost (*M*_T_) (see Data S7 for explanation).

Without a time limit, distance declines monotonically with increasing BMR, whereas under a fixed time limit the relationship becomes unimodal, with a single BMR yielding the greatest swimming distance (*Y*_max*_) among all possible maxima. Because this amounts to a type of model‐defined optimality, we refer to this value as the “optimal” BMR (Data S7: Figure S7.4), which should not be confused with any concept of biological optimality.

For any given continental gap distance, the time‐limited curve produces either a single value, at the special case of *Y*_max*_ corresponding to the “optimal” BMR, or, in all other cases, two possible BMR values capable of generating that distance. We define the interval between these values as the “BMR Range supporting a Successful Dispersal” (BRSD). The upper limit corresponds to BMRs above the “optimal” BMR value (governed by the no‐time‐limit relationship) given by:

(24) BMRmax=914×1000Available EnergyGapDistance149×109εAεpρλSCd^59

and the lower limit corresponds to BMRs below the “optimal” BMR value (determined by the time‐constrained portion of the curve) given by:

(25) BMRmin=100024×3600GapDistanceTime Limit145×910ρλSCd^εAεp

where gap distance is in km and time limit is in days.

The maximum possible dispersal distance (*Y*_max*_) is given by:

(26) Ymax*=Available Energy14×100εAεpρλSCd^514×24*36001000×Time Limit914

For the full derivation of equations (24–26) see Data S2 and for further explanation see Data S7.

The mathematically possible BRSDs can exceed the limits of the biologically plausible metabolic range obtained from the selected set of allometric equations (BRAL). Therefore, we constrained BRSDs using a set of rules based on the smallest and largest BMRs predicted by these allometric equations (SAL and LAL, respectively). The lower BRSD bound cannot fall below SAL, whereas the upper bound is not constrained by LAL and is defined by Equation (24) evaluated at the minimum gap distance for each case. An upper BRSD bound exceeding LAL is justified by the possibility of additional metabolic cost due to thermogenesis (*M*_T_). Although our model does not compute *M*_T_ directly, this feature provides qualitative insight into when thermogenesis (*M*_T_) could be tolerated while still permitting a successful crossing. Full explanation and boundary conditions are given in Data S7.

To standardize dispersal feasibility across taxa and gaps, we defined a percentage index (RBRSD) that represents the ratio of the overlapping segment between the BRSD and BRAL to BRAL. RBRSDs are given by:

(27) $$\text{RBRSD}=\frac{{\mathrm{BMR}}_{\max}-\max\left({\mathrm{BMR}}_{\min},\mathrm{SAL}\right)}{\text{BRAL}}\times 100\%,\text{if}\;\mathrm{LAL}>{\mathrm{BMR}}_{\max}$$

(28) $$\text{RBRSD}=\frac{\mathrm{LAL}-\max\left({\mathrm{BMR}}_{\min},\mathrm{SAL}\right)}{\text{BRAL}}\times 100\%,\text{if}\;\mathrm{LAL}\leq {\mathrm{BMR}}_{\max}$$

In this way, we have defined a metric analogous to a support value for a successful crossing of a given intercontinental gap. High RBRSD values indicate that a large portion of plausible BMRs would enable a successful crossing; low values indicate only a narrow feasible BMR window. Further explanation and worked examples are provided in Data S7.

## 3 Results

### 3.1 Model Outputs

#### 3.1.1 Extant Animals

**FIGURE 3:**
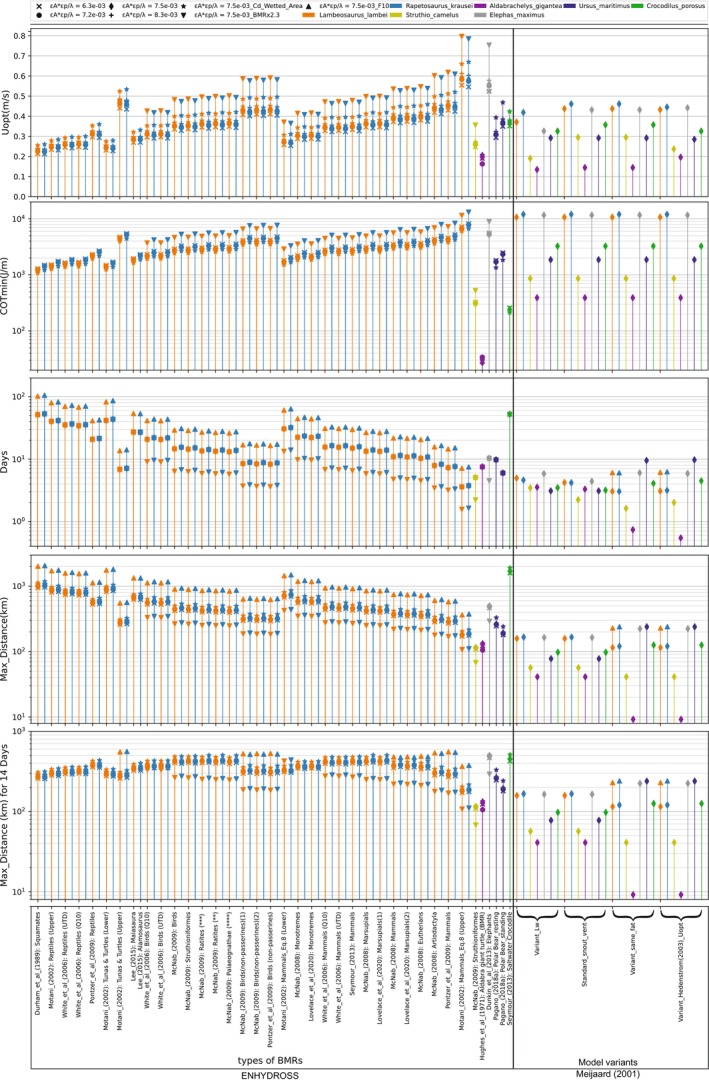
Left: Results for *U*_opt_, COT_min_, swimming duration (in days), maximum swimming distance (in km) and maximum swimming distance corresponding to a duration of 14 days per BMR formula used for each animal for the ENHYDROSS model. Right: Results for the same variables estimated using Meijaard's family of models (see Data S5). “Variant: Same fat*”* refers to calculations carried out using Meijaard's model but applying the same fat mass employed in the ENHYDROSS model where relevant. “Standard snout‐vent” refers to calculations carried out using Meijaard's standard methodology for which the waterline of the animal was equated to the snout–vent length. “Variant *L*_w_” refers to calculations carried out utilizing Meijaard's approach, but using the actual waterline length (*L*_w_) as measured by Henderson's buoyancy analyses or otherwise where possible (Data S6). “Variant Hedenstrom (2003) *U*_opt_ *”* makes use of Hedenström's (2003) Equation (6) for migration speed, but with the fat masses employed in ENHYDROSS. The various symbols represent sensitivity tests. For each sensitivity test, the reader is referred to Table 3. Note the logarithmic scale for the plots of COT_min_, swimming duration and maximum swimming distance (with and without a time limit).

The calculated *U*_opt_, COT_min_, swimming durations, and maximum swimming distances, with and without a 14‐day cutoff limit, along with the sensitivity tests for each parameter, are shown in Figure 3. This figure additionally shows the results from the preliminary model of Meijaard (2001) and its three variants evaluated herein.

Overall, optimal swimming speeds are mostly lower than 0.5 m/s, although in some cases, such as the elephant, speeds exceed this value (Figure 3). This is unsurprising given that the magnitude of feasible *U*_opt_ is relatively low, with a narrow range. The ENHYDROSS values obtained for the elephant, the polar bear, and the crocodile are within the range observed in reality (Table 4). However, even small differences in *U*_opt_ are amplified when estimating COT_min_, and values of the latter parameter are distinct for each animal (Figure 3). In the ENHYDROSS model, COT_min_ values are ranked the same way as mass values, i.e., animals with higher body mass have proportionally higher COT_min_ than those with lower body mass, with the exception of the crocodile (Figure 3). The crocodile, being semi‐aquatic, has more energetic economy for its mass relative to the other animals tested herein. The results for the swimming durations (Figure 3) are in alignment with the observed cases for the polar bear (resting BMR), tortoise, and crocodile in that they exceed the observed values (Table 4). Similarly, the results for the swimming distances (Figure 3) are in alignment (overestimates) with observed cases, and where there are underestimates, the influence of ocean currents is highly likely (polar bear with “resting” BMR, crocodile, tortoise), as evident from the results of passive drifting (Table 4). By contrast, the polar bear with the “standing” BMR results in underestimates of both swimming time and distance. However, while, drifting times under intermediate currents exceed the recorded 9 days, passive drifting distances appear too small (> 200 km less than recorded) to justify this case (Table 4).

**TABLE 4:** Main results of ENHYDROSS and Meijaard's model and its variants, compared with results obtained from passive drifting and recorded or inferred data from the literature.

		Sensitivity test	Elephant	Polar bear (resting BMR)	Polar bear (standing BMR)	Ostrich	Crocodile	Tortoise
ENHYDROSS Model	Uopt (m/s)	Preferred Parameters	0.56	0.31	0.37	0.26	0.37	0.20
		All tests range	0.52–0.75*	0.29–0.39	0.35–0.47	0.25–0.36*	0.35–0.42	0.16–0.21
	Max distance (km)	Preferred Parameters	498.6	263.7	192.6	115.7	1682.7	132.0
		All tests range	291.9*–515.9	247.1–331.2	180.5–241.9	67.8*–119.8	1594.4–1921.3	105.4–136.5
	Duration (Days)	Preferred Parameters	10.3	9.7	6.0	5.1	52.6	7.5
		All tests range	4.5*–10.3			2.2*‐5.1		
Meijaard's Model + variants	Uopt (m/s)	Standard	0.43	0.29		0.29	0.36	0.14
		All variants range	0.33–0.44	0.28–0.29		0.19–0.29	0.33–0.36	0.13–0.20
	Max distance (km)	Standard	164.1	77.7		56.7	97.6	41
		All variants range	164.1–224.0	77.7–239.2		41.1–56.7	97.6–125.7	9.2–41.0
	Duration (Days)	Standard	4.4	3.1		2.2	3.2	3.3
		All variants range	4.4–6	3.1–9.7		1.6–3.5	3.1–4.5	0.5–3.5
Passive Drifting	Max distance (km)	U_current_ = 0.25 m/s	150.7*–346.6	327.3	200.8	74.1*–170.4	1764.2	_
		U_current_ = 0.5 m/s	301.4*–693.2	654.5	401.6	148.2*‐340.8	3528.4	504.2
		U_current_ = 1 m/s	_	_	_	_	_	1008.4
	Duration (Days)	_	7.0*–16.0	15.2	9.3	3.4*–7.9	81.7	11.7
Recorded/Inferred data	Uopt (m/s)	_	0.43 (mean) ^[1]^	0.55 (mean of range: 0.194–1.03 ^[3]^)		_	0.41 (mean of 3 specimens with range: 0.256–0.506 ^[5]^)	_
			0.27–0.75 ^[2]^					
	Max distance (km)	_	48 ^[1,2]^	687 ^[4]^		_	2353 ^[6]^	740 ^[7]^
							2054 ^[6]^	
	Duration (Days)	_	_	9 ^[4]^		_	26.4 ^[6]^	6–21 days (?) ^[7]^
							38.8 ^[6]^	

*Note:* Values marked with asterisks indicate cases where the 2.3 × BMR enhancement was applied. Numbers in brackets correspond to the following references: Hadjisterkotis (2012) [1]; Johnson (1980) [2]; Durner et al. (2011) [3]; Pagano et al. (2012) [4]; Elsworth et al. (2003) [5]; Spennemann (2021) [6]; Gerlach et al. (2006).

When comparing the outputs of Meijaard's (2001) and ENHYDROSS models, some of the results in optimal speeds have substantial overlap (polar bear, ostrich, crocodile, tortoise), whereas others do not (elephant), although they all still fall within empirically observed ranges (Figure 3; Table 4). In the case of the elephant, the ENHYDROSS model's swimming speeds give substantially higher values (21.5%–74.6%) than those from all Meijaard (2001) models, under any test.

With regards to estimates of COT_min_, in nearly all cases the Meijaard (2001) model (and its variants) substantially exceed the ENHYDROSS results (Figure 3; Table 5). The only exception to this trend is the polar bear when the “standing BMR” is used. Similar to ENHYDROSS, the ranking of animals by COT_min_ in the Meijaard (2001) models follows the ranking by body mass. However, unlike ENHYDROSS, the Meijaard (2001) models do not exempt the crocodile from this pattern.

**TABLE 5:** COT_min_ results for ENHYDROSS and Meijaard's models expressed as coefficient multiples of Williams's (1999) allometric equation for COT (=7.79 × mass^0.71^ when converted to J/m units) for marine mammals.

Taxon	ENHYDROSS Model			Meijaard's Model + variants	
	Sensitivity Test	COT_min_ (J/m)	Coefficient multiple of William's COT_min_ equation	COT_min_ (J/m)	Coefficient multiple of William's COT_min_ equation
Elephant	Preferred Parameters	5229	2.29	11,639	5.1
	All tests range	5054–8932*	2.21–3.91*		
Polar bear (resting BMR)	Preferred Parameters	1691	4.63	1864	
	All tests range	1347–1805	3.68–4.94		
Polar bear (standing BMR)	Preferred Parameters	2315	6.33		
	All tests range	1843–2471	5.04–6.76		
Ostrich	Preferred Parameters	307	1.81	863	
	All tests range	296–524*	1.75–3.10*		
Crocodile	Preferred Parameters	243	0.38	3258	
	All tests range	213–257	0.33–0.40		
Tortoise	Preferred Parameters	27	0.36	391	
	All tests range	26–34	0.34–0.44		

*Note:* The coefficient multiple represents the additional costs of non‐specialists in aquatic locomotion. Values with asterisks mark cases where the 2.3 × BMR enhancement was applied.

In terms of swimming time, Meijaard's (2001) model estimates are substantially lower compared to the ENHYDROSS values in nearly all cases (Figure 3; Table 4). In addition, where recorded data exist (polar bear, crocodile, and tortoise), the Meijaard (2001) models give underestimated times, with the only exception being the polar bear in the two Meijaard variants that use the more animal‐specific fat mass (Table 4, Figure 3). For the latter case, the resulting durations are indistinguishable from those of the “resting” BMR bear when using the ENHYDROSS model. The same trends are observed with swimming distances, where once again the Meijaard (2001) models produce much smaller values than ENHYDROSS (except for the polar bear with the “standing BMR”) (Table 4, Figure 3). The Meijaard (2001) models also give higher distances compared to the elephant's empirically observed distance, but this difference is smaller than that between the observed value and that estimated by the ENHYDROSS model (Table 4). However, for the remaining cases, ENHYDROSS gives results that are closer to the observed data (Table 4).

#### 3.1.2 Nonavian Dinosaurs

There is a noticeable trend in the ENHYDROSS outputs that reflects the impact of inferred metabolic rate. Not surprisingly, when all other parameters are kept the same, an increase in BMR corresponds to an increase in *U*_opt_ and COT_min_ and a concomitant decrease in maximum swimming distance and duration (Figure 3). However, this changes radically when BMR is multiplied by the 2.3 “correction” factor (Figure 3). The latter results in the gap between the values obtained from ectothermic and endothermic BMRs increasing substantially, as is expected because the thermogenesis cost (*M*_T_) is absent in ectotherms. The results using the “mesothermic” tuna‐and‐turtle BMRs from Motani (2002) span the range of low ectothermic to high endothermic BMRs, whereas the results using the dinosaur‐tuned BMRs from Lee (2015) are comparable to those obtained from the other high ectothermic BMRs.

In general, for all endothermic BMRs, the two nonavian dinosaurs have relatively similar results, within a plausible range of *U*_opt_ and COT_min_ for animals of that mass and size when compared to values for endotherms such as the elephant. Similarly, the values obtained from ectothermic metabolic regimes suggest very long trans‐oceanic dispersal distances and durations, some of which appear to be unrealistic, at least at first sight. Nonetheless, we should remember that these results would have been considered sensible if similarly sized crocodiles and tortoises were tested (Figure 3) and most importantly that the extreme values are capped when a time threshold is applied (see Section 3.1.3 below). All tests suggest that the hadrosaur was slightly faster (higher *U*_opt_) than the titanosaur, despite the former's lower BMR. The exceptions are the wetted surface area test and those using the BMR allometric equations of Lee (2015); the latter are not directly comparable because they differ between taxa. Similarly, in all cases the hadrosaur is more economical (smaller COT_min_). Conversely, the titanosaur is estimated to have had a slightly higher maximum swimming distance and duration in all sensitivity tests. When swimming distances are standardized for body mass, the hadrosaur attains greater maximum distances than the titanosaur (see Data S7). Notwithstanding these differences in swimming efficiency and endurance, hydrostatic stability tests indicate that the hadrosaur had slightly greater lateral stability in water than the titanosaur, as reflected by its smaller negative metacentric height (Data S6).

The ENHYDROSS and Meijaard (2001) models have substantial overlap in optimal speeds under certain intermediate level metabolic regimes (Figure 3). However, the Meijaard (2001) models, as with the extant animals, give much higher COT_min_ in all but one case (and only marginally): the highest BMR case for the dinosaurs (the upper‐bound mammalian BMR equation of Motani (2002) when using the 2.3 × BMR test). Thus, reciprocally, the swimming distances and durations are much lower when compared to the ENHYDROSS estimates in most cases (Table 4, Figure 3). Overall, the results obtained from the Meijaard (2001) models for the nonavian dinosaurs are very similar to those for the elephant from the same models (Figure 3).

#### 3.1.3 Sensitivity Tests

The sensitivity tests reveal that varying *ε*_Α_ and *ε*_p_ within the tested range makes relatively little difference to the results. Thus, for the four sensitivity tests involving these two parameters, compared with the model using our preferred parameter values, the greatest difference is observed when *ε*_A_*ε_p_/*λ* = 6.3e‐03 (Figure 3; see also the ENHYDROSS core dataset available on Zenodo). In this test, the average relative difference for all taxa (including all dinosaur tested metabolic rates) is: *U*_opt_ ≤ 6.3%; COT_min_ ≤ 6.7%; maximum swimming distance ≤ 6.3%; and as explained previously the effect on swimming duration is null. On the other hand, utilizing the wetted surface area calculated drag coefficient, instead of the one calculated using the frontal surface, results in a mean 13.3% increase in both *U*_opt_ and swimming distance, and a mean 11.7% decrease in COT_min_. For all taxa, multiplying the BMR by 2.3 results in increasing, on average, the speed by 34.6% and the COT_min_ by 70.8%, whereas it lowers, on average, the distance by 41.5% and the time by 56.5%. By far the most pronounced effect, albeit expected given Equations (1) and (2), comes from doubling the fat mass (i.e., from 5.18% to 10.36%) in the case of the nonavian dinosaurs. This results in a doubling of the maximum swimming distance and duration, irrespective of the model used, illustrating that swimming distance and duration are proportional to fat mass for the reasons explained in Section 2.1.2.

#### 3.1.4 Effects of Water Deprivation Limits

**FIGURE 4:**
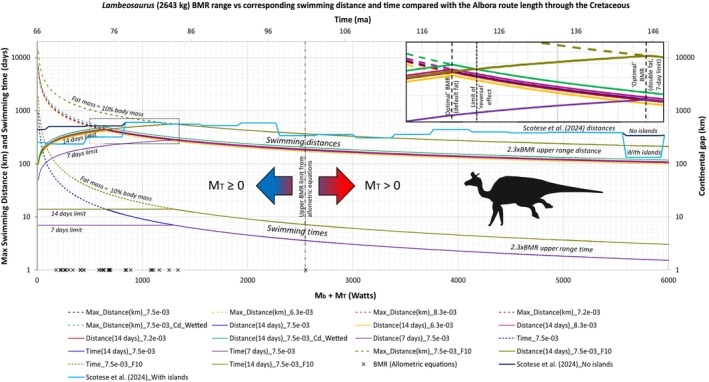
Trend curves showing how the possible metabolic rate (=*M*_b_ + *M*_T_) range varies with distance and time for *Lambeosaurus lambei*. Included are all the sensitivity tests and also the continental gap distances of Scotese et al. (2024) for the Albora route during the Cretaceous. Beyond the upper BMR limit of the allometric equations, a positive M_T_ is mandatory whereas within the BMR range obtained from the said equations, M_T_ may still be positive; for example, if a low BMR is multiplied by 2.3 and the result is still smaller than the upper limit. The enlarged section of the plot shows the two “optimal” BMRs and the “reversal effect” limit. The latter corresponds to the highest BMR among all sensitivity tests results for which the critical factor for longer distances reverses from fat to speed.

**FIGURE 5:**
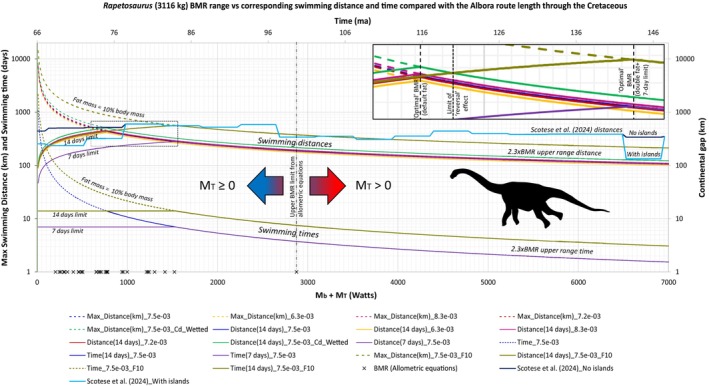
Trend curves showing how the possible metabolic rate (=*M*_b_ + *M*_T_) range with distance and time for *Rapetosaurus krausei*. Description of the main features of the plot is the same as that in Figure 4.

Without limits, cases with double fat mass and lower BMRs consistently yield the highest maximum swimming distances and durations for both nonavian dinosaurs. However, introducing a water deprivation limit caps distances below a certain BMR value, irrespective of fat mass, although cases with higher fat masses are affected the most (Figures 3, 4, 5). This can be seen in the resulting trend plots (Figures 4 and 5), in which the curves without and with a time limit for each test converge on a specific BMR. The point of convergence of the “no time limit” and “time limit” curves corresponds to specific “optimal” BMRs (see Section 2.8 and Data S7) for each sensitivity test and differs only when a different fat mass or time limit is used. In the default fat cases, for a 14‐day limit this convergence occurs at 652.7 W for *Lambeosaurus* and 769.5 W for *Rapetosaurus*. When the limit is halved to 7 days, the “optimal” BMR point for each animal shifts to a higher value, specifically twice the respective 14‐day limit value (i.e., 1305.3 W for *Lambeosaurus* and 1538.9 W for *Rapetosaurus*) (Figures 4 and 5). This also occurs under the 14‐day limit when the “double fat mass” curve meets its “no‐time‐limit” counterpart at twice the BMR value of the respective “optimal” BMR point of the default fat mass curve. Because in our case we have two “optimal” BMRs per dinosaur, related to the tests with the default fat and the test with the double fat mass (or equivalently 7‐day limit), we refer to them below as the first and second “optimal” BMRs, respectively.

When a time limit is imposed, fat mass is no longer universally the primary cause that results in longer distances from the set of sensitivity tests. Although beyond the second “optimal” BMR, higher fat mass always remains the dominant factor, speed becomes the dominant factor below a certain BMR. The BMR value upon which the critical factor reverses from fat to speed is herein called the “reversal effect” limit (Figures 4 and 5). This limit is always between the first and second “optimal” BMRs, depends on the sensitivity test, and differs between the two non‐avian dinosaurs. The “reversal effect” limit is higher for tests that result in higher speeds, with the highest being the “wetted surface area” test (Figures 4 and 5). Although the dominant factor for higher distances is fat mass, between this “reversal effect” limit and the second “optimal” BMR, the animals would not be able to convert all of their stored energy to swimming distance. In order to do so, they must have a BMR equal to or higher than the second “optimal” BMR. When comparing swimming distances with and without a time limit in Figure 3 one can see this “reversal effect,” where tests that give higher *U*_opt_ (particularly the “wetted‐surface area” test) result in greater swimming distances once water deprivation limits are applied.

Without a time limit, the larger‐bodied titanosaur consistently outperforms the hadrosaur in all tests per BMR equation in terms of maximum swimming distance, except the case where different allometric relationships were used for each animal, that is, the BMR equations of Lee (2015). In that case, all tests but the “wetted surface area” show the hadrosaur as slightly more capable of achieving longer distances (Figure 3). With the water deprivation limit imposed on the models, this is no longer the case, and the hadrosaur is predicted to have superior trans‐oceanic dispersal capacity relative to the titanosaur over several BMR regimes and tests (Figure 3). When body mass is equalized, the “optimal” BMRs of the two animals become identical, and differences in swimming distance consistently favor the hadrosaur regardless of time limits (Data S7: Figure S7.9).

Aside from the non‐avian dinosaurs, only the crocodile is affected by the water deprivation limit because the rest of the modeled animals have swimming durations of less than 14 days. However, the crocodile can last longer without water (as can be deduced from observations; see Table 4), so this cutoff does not represent a true water deprivation tolerance for this taxon; rather, it is merely a time limit applied for making homogeneous calculations and avoiding visual confusion in figures presenting results. This time threshold does not affect the distance estimates obtained from the Meijaard (2001) models because all of them result in swimming times well below the 14‐day water deprivation threshold (Figure 3; Table 4). This is the case even with the 7‐day threshold (Data S7: Figure S7.5) in all animals modeled, apart from the instance in which the accurate fat mass was applied to the polar bear.

### 3.2 Swimming Distances of Nonavian Dinosaurs Versus Cretaceous Intercontinental Distances

**FIGURE 6:**
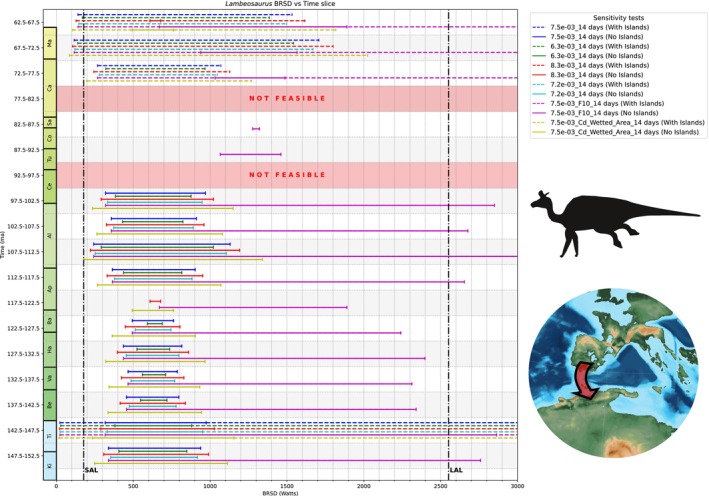
*Lambeosaurus lambei* BRSDs for the Albora route distance for every time slice, for every sensitivity test. Paleomap with unidirectional arrow shows the hypothetical direction of trans‐oceanic dispersal. Geological stratigraphic timeline is shown for comparison. Note that each time slice spans 5 million years but the data plotted inside each time slice are atemporal with regard to the inner span of 5 million years.

**FIGURE 7:**
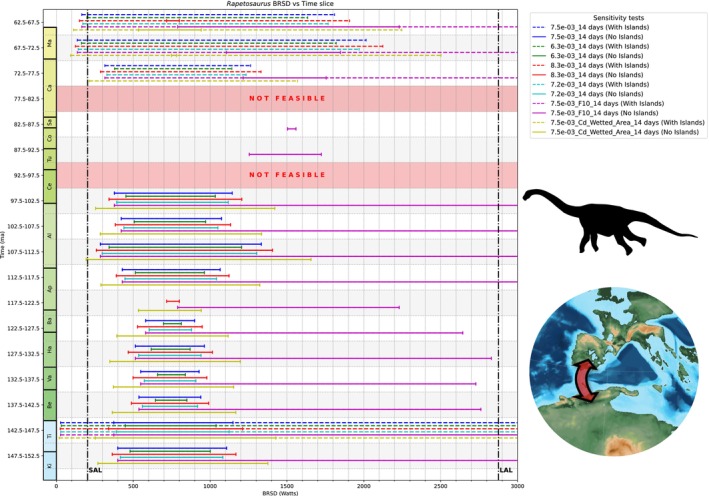
*Rapetosaurus krausei* BRSDs for the Albora route distance for every time slice, for every sensitivity test. Paleomap with bidirectional arrow indicates that the direction of trans‐oceanic dispersal could have been either way or both. Note that each time slice spans 5 million years but the data plotted inside each time slice are atemporal with regard to the inner span of 5 million years.

**FIGURE 8:**
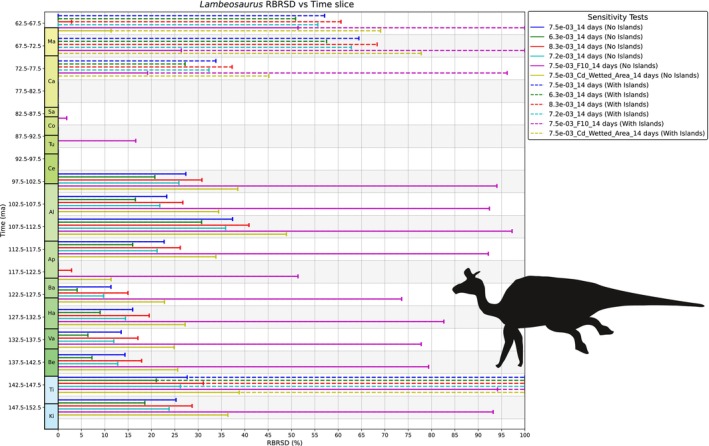
*Lambeosaurus* RBRSD values for the minimum continental gap between Iberia and Africa based on the paleogeographic model of Scotese et al. (2024) with and without islands present. Note that each time slice spans 5 million years but the data plotted inside each time slice are atemporal with regard to the inner span of 5 million years.

**FIGURE 9:**
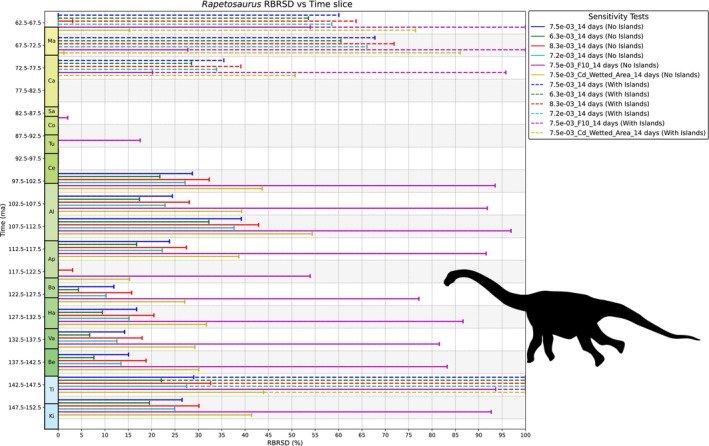
*Rapetosaurus* RBRSD values for the minimum continental gap between Iberia and Africa based on the paleogeographic model of Scotese et al. (2024) with and without islands present. Note that each time slice spans 5 million years but the data plotted inside each time slice are atemporal with regard to the inner span of 5 million years.

In Figures 4 and 5, the BMR of the nonavian dinosaurs is plotted as a continuum against swimming distance and time, with the Cretaceous continental gap distances between Iberia and Africa (with and without the presence of islands) shown for comparison. Figures 6 and 7 show BRSDs for a given continental gap distance corresponding to a specific time slice. Note that for visualization purposes, the x‐axis of the plots in Figures 6 and 7 was cut off at BMR = 3000 W, which spans just over the upper range values of the allometric equations. The entire span of values can be seen in the ENHYDROSS core dataset available on Zenodo. Finally, Figures 8 and 9 show the RBRSD indices which reflect the feasibility of dispersal given the range of possible BMRs (see Section 2.8 and Data S7 for explanation).

Four points stand out most prominently from Figures 6 and 7. First, there are no combinations of parameters that allow for a crossing of the Albora route during 97.5–92.5 Ma and 82.5–77.5 Ma. Second, with a conservative fat value (5.18%) and without accounting for islands, for most cases BRSDs are small and anchored at intermediate ranges of BMR (with “optimal” BMRs of 652.7 W for *Lambeosaurus* and 769.5 W for *Rapetosaurus*), while RBRSD values are almost always below 45% for both animals. Notably, under these conditions, the highest RBRSDs of 30.7%–48.9% for *Lambeosaurus* and 32.3%–54.4% for *Rapetosaurus* are during the early Albian (112.5–107.5 Ma). By contrast, when the fat mass is doubled, BRSDs increase and are now anchored around twice the BMR values for each animal, corresponding to the new “optimal” BMRs. Respectively, the RBRSDs increase substantially and often exceed 75%, while for the Albian they reach just over 95% for both dinosaurs. In addition, the higher fat mass value means that dispersal becomes feasible during the periods 77.5–72.5 Ma and 92.5–82.5 Ma. Furthermore, some other periods where dispersal was possible only under a very narrow range of BMRs now predict feasible dispersal under much wider BRSDs (Figures 6, 7, 8, 9). Third, unsurprisingly, the presence of islands increases the range of possible BMRs that the dinosaurs could have possessed if they were to make a successful crossing via active swimming. In fact, when combined with the 10.36% fat mass value, the presence of islands consistently corresponds to RBRSD values ranging from just over 95% to 100% in all relevant cases. Consequently, for the vast majority of the BRAL range (except the lowest values (< 300 W), which correspond to the majority of the ectothermic BMRs), the dinosaurs could have easily made the crossing if they island‐hopped (Figures 6, 7, 8, 9). Fourth, when higher fat mass or islands are present, the upper limits of BRSDs sometimes exceed the largest values from the allometric equations, widening the respective BRSDs substantially. All numerical data underlying the figures are provided in the ENHYDROSS core dataset (Excel file) available on Zenodo (DOI: https://doi.org/10.5281/zenodo.18015597).

## 4 Discussion

### 4.1 Implications for Extant Model Animals

#### 4.1.1 Acknowledging the Elephant in the Sea

The elephant is one of the most important cases that we need to understand, mainly because of its size and the few datapoints regarding its habitual swims. This animal also serves as an important model because it is loosely analogous, in terms of biomechanics (and debatably in terms of metabolism), with extinct large animals such as nonavian dinosaurs. It is therefore reassuring that more than one modeling approach (i.e., ENHYDROSS and Meijaard's (2001) models; Figure 3), as well as all sensitivity tests of our model, provide estimates that never fall outside the observed range of swimming speeds (Figure 3, Table 4). Whether the observed cruising speeds represent some form of energetic optimization is unknown currently, but at least our model yields results based on an appropriate definition of what the optimal swimming speed would be, given certain parameters and assumptions.

On the other hand, estimated maximum swimming distances should be treated with caution: both the ENHYDROSS and Meijaard (2001) models indicate values that are substantially higher than the longest known case (48 km) (Johnson 1980). For the sensitivity tests that vary the hydrodynamic parameters, the ENHYDROSS model yields distances that are typically 2–3 times greater than those derived from the Meijaard (2001) models; this seems to be caused mainly by the contrasting fat masses used by the different approaches. When the same fat mass is used, the difference is generally smaller, though still large (ENHYDROSS distances are 2.2 times higher). However, when the 2.3 × BMR enhancement is applied, along with the same fat mass, the difference decreases substantially (ENHYDROSS distances are 1.3 times higher). This shows that the two approaches tend to converge when some amount of thermogenesis is occurring. Meijaard's (2001) use of Prothero's (1995) equations for calculating fat mass provides an average value for all mammals spanning many orders of magnitude, with these equations requiring only one input parameter: body mass. Therefore, these equations may not be appropriate to all mammals under all situations (e.g., low nutritional or poor health conditions), nor do they account for natural variation in fat mass percentages among different species, some of which are inherently more obese than others (see Data S1). If one calculates the fat mass of an elephant using the confidence intervals of these equations (181.7–249.67 kg) and applies these values in the Meijaard (2001) models, we obtain maximum dispersal distances (140–192 km) and durations (3.75–5.16 days) that differ substantially, and the upper‐end fat mass is still significantly lower than the value we used herein based on the literature (290.65 kg). It is not unheard of for an elephant to have a fat mass between 2.01% and 24.59% of total body mass, as can be seen in Chusyd et al. (2021), from which our average was taken. The above points justify our concerns regarding the risk of: (1) using a single allometric equation for a diverse clade to obtain fat masses for a species without accounting for taxonomic differences in anatomy and physiology; and (2) using a single value when using fat mass as a proxy for energy reserves when modeling dispersal limits and testing biogeographic hypotheses.

The results of our study indicate that elephants can swim for substantial distances, and cases in which closely related extinct species are hypothesized to have swum comparable distances can be usefully revisited. For example, the 70–80 km distance from the Anatolian coast to Cyprus, which would have been shorter during glacial/low eustatic periods (30–60 km according to Hadjisterkotis 2012), is well below the maximum swimming distances for the elephant estimated by all models tested herein. Thus, even without the assistance of oceanic currents, such journeys seem entirely feasible for the ancestors of the Quaternary species *Paleoloxodon cypriotas* and *P. xylophagou* (Athanassiou et al. 2015; Hadjisterkotis 2012). Moreover, such inferences are further strengthened when one calculates the time required for crossing from Anatolia to Cyprus using the ENHYDROSS results. Given our preferred parameter result for *U*_opt_, we can estimate that it would take (60 km/0.56 m/s =) 1.24 days for a 60 km crossing and 1.65 days for an 80 km crossing. Even if we cannot be certain at present whether elephants can last without water for 10.3 days (preferred parameter test result), 1.24–1.65 days is well under the observed water deprivation limit for elephants (Data S7: Table S7.1). Hadjisterkotis (2012) estimated the crossing time of elephants from mainland to island to be a minimum of 15 h based on an average swimming speed of 1.5 km/h (=0.42 m/s). We can show that this corresponds to a minimum distance of (1.5 km/h * 15 h=) 22.5 km. Even so, the shortening of the actual distance (30–60 km) can be attributed to the effect of sea currents (Hadjisterkotis 2012). On the other hand, a 15‐h swimming duration would correspond to a distance closer to 30 km using the speed (0.56 m/s) predicted by our model. The visibility of the nearby opposite shore may have influenced the animal to swim faster than its optimal speed in order to minimize its time as opposed to its COT. If this was the case, then the duration of the swim might have been shorter than that obtained using our model and closer to the estimate given by Hadjisterkotis (2012). Alternatively, even just passive drifting aided by mild currents is a permissible option for such relatively small distances, and even with extra cost for thermogenesis assuming temperatures below thermoneutrality (Table 4). There are many other similar cases involving putative trans‐oceanic dispersal of elephants and their close relatives from the mainland to various Mediterranean and Indonesian islands (van der Geer et al. 2011 and references therein) which would potentially benefit from the application of our modeling approach in the future.

#### 4.1.2 Swimming Polar Bears and Survival in the Arctic Sea

The polar bear represents a critical example of an animal affected by climate change, as melting sea ice reduces alternative terrestrial “over ice” migration routes, forcing them to undertake increasingly longer swims to reach their hunting grounds (Durner et al. 2011; Monnett and Gleason 2006; Pagano et al. 2012; Pilfold et al. 2017). Given the rising energetic demands of these long swims, understanding the bears' swimming energetics and endurance limits is essential for conservation efforts. The ENHYDROSS model operates under the condition of null‐thermogenesis, which, as stated previously, can be satisfied for endotherms in two distinct ways: thermoneutral water temperatures or complete thermal substitution (see Section 2.1.3). In the case of polar bears swimming in Arctic waters, the former is highly unlikely given near‐freezing water temperatures. We therefore assume that null‐thermogenesis is achieved via complete thermal substitution (i.e., thermoregulatory heat demands are fully compensated by heat generated during locomotion), which, although optimistic, remains plausible and informative as we will see below.

The ENHYDROSS' *U*_opt_ prediction falls on the lower end of the spectrum of empirically observed values, as is also the case for the Meijaard (2001) models (Table 4). However, we should consider that the recorded speeds of bears in the wild probably incorporate the effect of ocean currents (Pagano et al. 2012). Thus, the swimming speed range of these animals is likely skewed towards the higher limit, which is also supported by our passive drifting results (Table 4). Given that ENHYDROSS (and Meijaard's 2001 models) significantly underestimate maximum swimming distances, it is likely that favorable ocean currents operated in the case of Durner et al.'s (2011) reported bear. Only passive drifting, with the action of favoring intermediate currents (0.5 m/s), results in both traveling distances and durations exceeding or approaching those recorded by Durner et al. (2011) (Table 4). More importantly, this occurs only when the “resting BMR” is used. Since the ENHYDROSS result for the ‘standing BMR’ already gives a far shorter swimming duration (6 days) compared to the observed case, this strongly suggests that “resting BMR” is more appropriate for use in the ENHYDROSS model (if we accept for validation purposes that the recorded case of the 9‐day swim involved only active swimming). If results from ENHYDROSS reflect reality for the “resting BMR” case, then the individual polar bear observed by Durner et al. (2011) could have lasted ~17 more hours which is a considerable time, but relatively not very far from the maximum recorded swimming duration. The 9‐day limit recorded by Durner et al. (2011) was exceeded by very similar amounts of time (12–17 h) in the Meijaard (2001) model variants when the accurate fat mass was used (Figure 3, Table 4). Because swimming speeds produced by the two models (ENHYDROSS and Meijaard) were very similar (excluding the “wetted surface area” test case), the same goes for swimming distances (Figure 3, Table 4). Thus, when the same fat mass is used, at least for the polar bear, both models produce very similar results in terms of swimming duration and distances.

The major problem when comparing the ENHYDROSS and Meijaard's (2001) models arises when seeking to understand the relative contribution of ocean currents with regard to swimming distance. Since the models we tested here give underestimates in this regard, and because ocean currents can indirectly extend survival time at sea if animals ride the current while remaining inactive, it presents a problem for clearly demonstrating which model is better. If, for example, the inactivity pauses are long, then the recordings of Durner et al. (2011) can be interpreted within either model because the results will gravitate towards our passive drifting estimations. If, on the other hand, the inactivity durations are fewer and smaller in extent, then either model could be accurate depending on the intermittency of activity. Moreover, such comparisons should ideally be made when the *M*_T_ is known and accounted for. Unfortunately, we do not have such data to work with at present and we did not attempt to experiment with different possible scenarios here because this would have made our study substantially longer.

In short, without accounting for: (1) the effects of the specific ocean currents of Durner et al.'s (2011) case; (2) the length of time when the swimming bear was inactive; and (3) the exact metabolic cost (accounting for both *M*_b_ and *M*_T_) for the body of that specific bear, we cannot obtain a direct validation of the model. When considering the temperature range of applicability for either model and the expected values at near‐polar ocean water temperatures, both ENHYDROSS and Meijaard's (2001) models are likely producing overestimates that are masked by ocean current effects on the observed data.

#### 4.1.3 Can Large Flightless Birds Cross Oceans via Swimming?

Like most terrestrial animals, ratites, such as emus and cassowaries, can swim and are often considered to be adept swimmers (Bakker 1971; Chernova and Fadeeva 2009; Gilliard 1958). However, the latter view has typically been propagated by anecdotal or non‐peer reviewed reports among zoologists, farmers, and others with opportunities to observe ratites in the wild; thus, rigorous studies addressing this aspect of ratite biology have not been published to date. The majority of sources related to ratite swimming that we have been able to locate are online videos showing the birds crossing rivers, or bathing to cool off in shallow ponds, lakes, or near to the shoreline (Data S8: Table S8.1). In all these cases, swimming speed estimation is nearly impossible due to the parallax effect among other things, while distance can only be estimated very roughly and never exceeds a few hundred meters. Nevertheless, it is worth highlighting a few of these cases. Recently a cassowary (*Casuarius* sp.) was reportedly observed emerging from the sea in Bingil Bay, north Queensland, where it swam a distance of about 200 m according to a witness (Table S8.1). Multiple videos online show emus crossing rivers (Table S8.1), involving swimming distances of probably a maximum of a couple of hundred meters. Another case concerns lesser rheas ( *Rhea pennata* ) sighted on a small island (Isla de Rey) in the Deseado River (Annick Morgenthaler, personal communication 2023). The island is about 300 m from the mainland on one side and 400 m from it on the other side. They all perished on that island for unknown reasons (Annick Morgenthaler, personal communication 2023), but exhaustion or hypothermia from swimming to reach the location is a plausible explanation, even though the crossing distance was not very large. This suggestion is supported by a similar and perhaps the most informative recorded case concerning 20 subadult lesser rheas that were observed swimming until exhaustion (four of which died the following day, possibly from hypothermia) over a distance of 400–500 m (Annick Morgenthaler, personal communication 2023; see Data S8, Table S8.1 for video link.). This event took place in the evening, with air and water temperatures being approximately 10°C–15°C and 10°C, respectively, and wind speeds less than 10 knots (Annick Morgenthaler, personal communication 2023). This represents very cold conditions for such birds whose feathers are most likely neither waterproof nor impenetrable to water, and in addition lack substantial adipose fat for insulation in water (for further explanation see Data S8). Thus, despite their capacity to swim or cross rivers, cold water would appear to be a strong barrier to successful dispersal for rheas, and perhaps other ratites.

The ENHYDROSS and Meijaard (2001) models yield plausible *U*_opt_ estimates for an ostrich, but validation of these speeds and other results must await future empirical observations. As evident from the few recorded cases listed above, a large, flightless bird with a cursorial lifestyle would seem to be too awkward a swimmer to be able to survive a swim of 115.7 km (5.07 days) as estimated by the ENHYDROSS model (preferred parameters), or even the shorter maximum swimming distance (67.8 km; 2.2 days) obtained from the 2.3 × BMR enhancement analysis. We should also bear in mind that the baseline ENHYDROSS results represent favorable, best‐case conditions associated with null‐thermogenesis, such as relatively warm water temperatures and typically calm waters. Unlike the polar bear case (Section 4.1.2), we do not consider complete thermal substitution to be a plausible scenario for a swimming ostrich. Instead, we speculate that, given their relatively poor insulation, heat loss in cold, wet conditions would likely far exceed any heat generated by muscular activity during swimming. If dispersing ratites were to experience more typical ocean conditions (e.g., strong waves and relatively low temperatures), the realized swimming distances would almost certainly be smaller than those predicted by our model. Even when applying the 2.3 × BMR enhancement as a crude allowance for additional thermogenesis, the resulting distances are still likely to represent optimistic upper bounds. Thus, for the ostrich, the ENHYDROSS model (and to a lesser degree the Meijaard (2001) models) yields swimming distances (see Table 4, Figure 3) much greater than what is anticipated given the body of indirect evidence available so far.

The above has interesting implications in terms of ratite biogeography. Earlier studies (e.g., Bourdon et al. 2009; Cracraft 1974, 1973; Johnston 2011) advocated that the present‐day distribution of ratites could have been a result of vicariance, driven by the breakup of the Gondwanan supercontinent during the Cretaceous and early Cenozoic. By contrast, molecular evidence has challenged this hypothesis, suggesting instead a more complex diversification pattern, including multiple losses of flight after the birds colonized island landmasses; this, in turn, suggests numerous episodes of trans‐oceanic dispersals (Allentoft and Rawlence 2012; Baker et al. 2014; Cooper et al. 2001; Grealy et al. 2017; Haddrath and Baker 2012; Maderspacher 2022; Mitchell et al. 2014; Phillips et al. 2010; Smith et al. 2013; Widrig and Field 2022; Yonezawa et al. 2017). While a smaller‐bodied, volant ancestor would effectively eliminate any need to invoke dispersal via means of swimming or drifting/rafting, some authors have noted that sweepstakes dispersal may explain some distributional patterns (Cooper et al. 1992; Yonezawa et al. 2017). Should future investigators want to address such possibilities, they could make use of our model and estimate whether these animals were capable of such swimming distances.

#### 4.1.4 Can Aldabra Tortoises Swim to Africa? Reexamining a Dispersal Case

It is clear that our model results are consistent with the inferences made by Gerlach et al. (2006) regarding trans‐oceanic dispersal for the Aldabra giant tortoise, albeit in terms of maximum swimming duration rather than distance. Gerlach et al. (2006) estimated that an Aldabra tortoise, which washed ashore in Tanzania on the east coast of Africa, would have taken 6–17 days to disperse the 740 km from Aldabra. This estimate assumes a westward drift aided by the South Equatorial Current (1–3 knots or 0.51–1.54 m/s), which would have contributed to the tortoise's overall velocity. This duration aligns with our estimated maximum swimming time of 7.5 days (Table 4). However, Gerlach et al. (2006) also noted that with a northeast monsoon slowing down the South Equatorial Current at the time, a 3‐week journey was more likely. Our passive drifting estimates, however, suggest a shorter maximum duration, with the parameters tested in our model yielding a maximum of 11.7 days. (Table 4). Nevertheless, it is important to note that we modeled our tortoise's fat mass based on a closely related species ( *Gopherus agassizii* ) due to limited data. To avoid bias in selecting either the higher or lower end of the range to fit the results, we chose the median value from this range of estimates. However, this does not necessarily imply that the fat mass we used was the most accurate, and the tortoise could well have been more obese. Had we used the upper end of this range (i.e., 2.5% of body mass), passive drifting times would extend to 18.24 days, closer to but still below the 3‐week estimate, while ENHYDROSS would increase the duration to 11.72 days. In addition, we know that the tortoise recorded by Gerlach et al. (2006) gained 2 kg (+8% of its original mass) 3 months after its arrival on Africa, an unknown portion of which was likely replenished as energy reserves. Even half of that (i.e., 1 kg of fat, or 4% of body mass) could have increased the results for duration up to 18.76 days for ENHYDROSS and 29.18 days for passive drifting. That said, based on current knowledge, including recent findings on *Testudo hermanni* (Tomović et al. 2020), tortoises appear to possess extremely low‐fat reserves, with fat bodies contributing only 0.0% to 0.5% of total body mass. Although occasional cases of obesity have been reported in overfed captive individuals, such conditions have not been observed in wild populations to date (Tomović et al. 2020). In contrast, the liver appears to function as a more substantial energy reserve, accounting for approximately 2.7% to 7.2% of body mass in tortoises (Tomović et al. 2020 and references therein). While the liver supports metabolic demands during reproduction, its role likely extends beyond reproductive periods, suggesting a broader function in energy storage (Tomović et al. 2020 and references therein). Therefore, if Gerlach et al.'s (2006) three‐week estimate for the tortoise's journey is accurate, it would more likely suggest that the tortoise relied on greater energy reserves than those assumed in our model, rather than indicating a flaw in ENHYDROSS.

In either case, it is unlikely that the tortoise had swum the entire distance without the aid of currents given the substantial underestimates of swimming distances by our model and the tortoise's rather slow swimming speed compared to even mild ocean currents. Instead, results from passive drifting alone (Table 4) strongly suggest that intermediate to strong currents (> 0.74 m/s) would be sufficient to carry a tortoise the 740 km from Aldabra to Africa, as hypothesized by Gerlach et al. (2006). On the other hand, an intermediate current (0.5 m/s) alone results in maximum drifting distances that fall short by over 200 km. Thus, a combination of favorable currents, passive drifting, and active swimming is the most plausible explanation for the trans‐oceanic dispersal event documented by Gerlach et al. (2006).

Tortoises have colonized remote oceanic islands like Madagascar, the Comoros, Mauritius, the Seychelles, and the Galapagos (Austin et al. 2003). Several anatomical adaptations greatly aid them in reaching such isolated islands, including their natural buoyancy, the positioning of their lungs near the top of their shells that aids in self‐righting, and their long necks, which help them keep their heads above water for easy breathing (Austin et al. 2003). In the case of the Galapagos, the distance from the source landmass, South America, exceeds 1000 km (Caccone et al. 1999). Our results strongly advocate for this to be a case of dispersal via mostly passive drifting and ocean currents rather than active swimming.

#### 4.1.5 Crocodiles Are Extremely Capable Oceanic Dispersers

The maximum swimming distance results (and their corresponding swimming durations) for our 4.82 m crocodile are extraordinary across all the tests, not just because such estimates are of the same order of magnitude as the maximum observed long‐distance dispersals (Spennemann 2021) (Table 4), but also because these results were obtained without taking into account sea currents which are known to be utilized by saltwater crocodiles. If such current‐assisted dispersal were factored in, this could essentially make the species capable of traveling nearly anywhere on the globe, without any problem in terms of energy reserves. This is evident from the passive drifting results which, for only a moderate 0.5 m/s current, extend the distance beyond 3500 km and a duration of 81.7 days (Table 4), further than any speculated distance presented by Spennemann (2021). Moreover, in theory, the energy reserves may not even be a limiting factor for these animals because presumably they can feed on marine fish *en route*. On the other hand, as ectotherms, even large crocodiles need to swim in relatively warm waters. Modern sea surface temperatures (SSTs) rarely exceed 30°C (Pearson et al. 2001), which is probably the main reason crocodiles are not found at high latitudes or in the colder waters of the Atlantic. By contrast, Cretaceous and early Cenozoic SSTs were warmer than today, with tropical SSTs of 28°C–32°C (Pearson et al. 2001). For the Late Cretaceous (100.5–66.0 Ma) in particular, SSTs may have reached 27°C–37°C in mid‐ to high‐southern latitudes (O'Connor et al. 2019). Such SSTs would have negated any disadvantage for ectothermic reptiles in terms of their lower critical temperature, where activity ceases due to hypometabolism. As a result, extinct crocodylian relatives, consisting of lineages of dyrosaurids and gavialoids, are known to have thrived in many of the Late Cretaceous to early Cenozoic oceans that were warmer than those of today (Barbosa et al. 2008; Hastings et al. 2011; Vélez‐Juarbe et al. 2007). Many of these marine taxa presumably shared physiological and behavioral similarities with modern saltwater crocodiles and would have been capable of long‐distance trans‐oceanic dispersals (Burke et al. 2025; Pligersdorffer et al. 2024). Indeed, a recent phylogenetic‐based biogeographic analysis found support for trans‐oceanic dispersals of numerous crocodylian (and closely related extinct) lineages during the Cretaceous and early Cenozoic, including journeys across the Atlantic Ocean on either side of the Equator (Groh et al. 2023). This might have been considered highly plausible for at least many of these lineages, even before our study's results, given the observed dispersal abilities of the extant *Crocodylus porosus* (Table 4) and the circumtropical distribution of the genus, *Crocodylus*, more broadly (e.g., Meredith et al. 2011; Oaks 2011). Nonetheless, a direct northern Africa–North America trans‐Atlantic crossing distance during the latest Cretaceous, as measured using GPlates, varies between c. 2600–4200 km (depending on the chosen endpoints) based on the reconstructions of Scotese et al. (2024). In light of our results, such a journey would be considered less likely, unless assisted by oceanic currents. Suitable westward and eastward currents are known in the Atlantic today (i.e., the westward North and South Equatorial Currents and their eastward countercurrents, together with transport associated with the Guinea Current system) and have potentially made long‐distance dispersals to islands feasible for crocodiles in modern times (e.g., Ceríaco et al. 2024). With regard to a latest Cretaceous westward current (Gordon 1973) aiding dispersal from western Africa to South America, even assuming no feeding *en route* (i.e., no re‐fueling) during dispersal, island‐hopping would not necessarily have been required for these vagile extinct crocodylian lineages provided they were of a substantial size and/or had suitable fat reserves (e.g., ~10% of body mass for an animal c. 4–6 m). Furthermore, Groh et al.'s (2023) biogeographic analyses also recovered support for multiple trans‐Pacific dispersals for many crocodylian clades at various times during the Cretaceous and the Cenozoic. If we consider the Cretaceous for illustrative purposes, such distances typically exceed 15,000 km (e.g., between South America and Southeast Asia) when measured in GPlates using the Scotese palaeogeographic models (C. Scotese 2016; Scotese et al. 2024, 2021). Our modeling approach enables us to evaluate the feasibility of such scenarios further. Given a hypothetical ocean current of 0.5 m/s, added to our estimated crocodile optimal speed of ~0.37 m/s, ENHYDROSS (with preferred parameters) indicates that the time required to traverse 15,000 km would be 199.7 days (i.e., 15,000 km × 1000 m × (0.5 + 0.37) m/s÷24 h÷3600 s). This is nearly four times the maximum swimming time (52.6 days) for our crocodile model and more than twice the maximum passive drifting time (81.7 days). Thus, although c. 200 days is well within the lifespan of an individual crocodile, it seems improbable that the presence of closely related taxa 15,000 km apart resulted from a single dispersal event. However, if the total distance is divided into smaller sections of one‐fourth or less to account for stopping time on small islands, the duration would be within the energetic limits we have calculated. Moreover, each of these travel segments between stop‐overs need not be performed sequentially by a single individual or even a single species, but by a lineage of multiple species: after all, the dispersal signals that correspond to such extreme distances (15,000 km) have been detected by biogeographic analyses using largely extinct species, so missing data are rife and evolutionary events that required tens or hundreds of thousands of years will often appear geologically instantaneous.

### 4.2 Implications for Nonavian Dinosaur Paleobiology

#### 4.2.1 Swimming‐Related Differences Between Hadrosaurs and Titanosaurs

Overall, the results of our study indicate that hadrosaurs were more efficient swimmers than titanosaurs. They generally exhibit higher optimal swimming speeds, lower cost of transport, and greater lateral stability in water (see Results; Data S6). The exception in terms of speed, noted in the case of the wetted surface area test, arises because a larger portion of the titanosaur is emergent when floating relative to the hadrosaur (see Figure S6.1), increasing the wetted surface of the latter relative to the former. Interestingly, the *C*_d_ of the titanosaur is greater than that of the hadrosaur under this scenario, whereas the opposite is true when the frontal surface formula is applied. This indicates that the relative impact of the *C*_d_ is not as great as that of the surface area (*S*) used in the *U*_opt_ equation.

By contrast, titanosaurs, due to their larger body mass, are predicted to achieve slightly greater maximum swimming distances and durations under many parameter combinations (see Results). However, when mass is equalized, the hadrosaur outperforms the titanosaur across all metrics because body mass influences basal metabolic rate and fat mass estimates through their respective allometric equations, which in turn affect swimming distances and durations via Equations (1) and (2) (see Data S7 for details).

These performance differences are consistent with anatomical and biomechanical contrasts between the two clades. Hadrosaurs possessed relatively more agile hind limbs, greater ankle flexibility, and limb proportions that likely reduced drag during recovery strokes. In addition, a facultative bipedal swimming gait may have further reduced interference drag (for details see Data S3 and S7), potentially enhancing swimming efficiency. Additional discussion and Supporting Information are provided in Data S7.

#### 4.2.2 Implications for Nonavian Dinosaur Palaeobiogeography

Although the feasibility of trans‐Tethyan trans‐oceanic dispersal has been proposed by numerous studies, the current work represents the first time that this has been demonstrated in terms of biophysical means.

The first period of potential sea‐crossings we examine is the “middle” Cretaceous. Out of the two modeled dinosaurs, only titanosaurs are of relevance during this period and the timing of possible dispersal(s) in question is sometime(s) during the Aptian–Cenomanian (see section 2.7.1 and reviews in Díez Díaz et al. 2025; Upchurch Upchurch In Press). Our results agree well with this timing, as can be seen in Figure 9 for the interval 117.5–97.5 Ma (middle Aptian–early Cenomanian), albeit with RBRSDs typically in the range of ~20%–45% for most tests (Figure 9). The early–middle Albian (112.5–107.5 Ma), in particular, stands out as the most likely period for crossings (RBRSDs > 30%) under the conservative fat mass (Figure 9). With substantial fat mass (~10%), crossings were apparently feasible throughout the Aptian–Cenomanian, even when temperatures were below thermoneutral (*M*_T_ > 0), as evident by the upper limits of the respective BRSDs, which exceed the upper BRAL limits. On the other hand, the late Barremian–middle Aptian (122.5–117.5 Ma) interval is shown to have very small BRSDs (comprised of BMR values from the mid‐range of BRAL) under the conservative fat mass case, while not all sensitivity tests allow for a crossing. The reduced RBRSDs observed for this time reflect the early Aptian (120 Ma) sea‐level highstand (Scotese et al. 2024), which resulted in more extensive flooding of continental areas and, consequently, longer oceanic distances. This does not contradict, but rather refines, previously proposed windows of dispersal.

The second period of potential faunal exchange we have examined is the latest Cretaceous, specifically the Campanian–Maastrichtian, during which time both titanosaurs and hadrosaurs are hypothesized to have crossed between Europe and Africa. The latest Cretaceous is considered a period of sea‐level regression (Scotese et al. 2024) and the middle Campanian and Campanian/Maastrichtian boundary sea‐level drops have been previously recognized as favorable intervals for dispersals (e.g., see Csiki‐Sava et al. 2015). Our results partially support the conclusions of previous studies regarding trans‐Tethyan exchanges in general (e.g., Buffetaut 1989; Canudo et al. 2009; Csiki‐Sava et al. 2015; Ezcurra and Agnolín 2012; Gheerbrant and Rage 2006; Le Loeuff 1991; Longrich et al. 2024, 2021; Sereno et al. 1994; Vila et al. 2022; Weishampel et al. 2010). However, aside from a couple of tests with slightly above zero BRSDs, this appears to be the case only if substantial fat mass and/or the presence of islands are incorporated. When both of these factors operate, the RBRSDs increase to values ranging between just over 95% and up to 100%. At the same time, crossings are feasible even with substantial additional energetic demands due to thermogenesis (*M*_T_ > 0). Such trans‐oceanic dispersals appear to have been particularly feasible during the middle Campanian–end‐Maastrichtian (77.5–66 Ma). During this time, titanosaurian lineages could have crossed the Alboran route in either direction. Hence, the conclusions of other authors (e.g., Sallam et al. 2018), which indicate a dispersal to Africa of a European titanosaurian lineage that led to *Mansourasaurus*, are supported by our results. During the same interval, lambeosaurine hadrosaurs could indeed have swum from Iberia to Africa, giving rise to the Moroccan species *Ajnabia odysseus* and *Minqaria bata*, supporting the hypothesis of Longrich et al. (2024, 2021). In addition to the commonly recognized late Early and latest Cretaceous periods as viable windows for trans‐Tethyan dispersal of dinosaurs, our findings suggest that such crossings might have been possible at certain other times during the Early Cretaceous and the latest Jurassic (Figures 6, 7, 8, 9).

Aside from identifying potential time intervals when dispersal was feasible, our results also pinpoint periods where crossings were effectively impossible, helping to eliminate uncertainties in dispersal scenarios and redirect research towards alternative explanations for observed distribution patterns. For example, during the interval 100–80 Ma, global sea level was at a highstand and many continental regions were flooded as a result (Scotese et al. 2024). The short‐lived late Cenomanian–early Turonian and Santonian–Campanian drowning events are also observed in the depositional record of the Adriatic‐Dinaric carbonate platform in the central European archipelago (Mezga et al. 2006 and references therein). This is well reflected in our results: the middle Cenomanian to middle Turonian (97.5–92.5 Ma) and early to middle Campanian (82.5–77.5 Ma) intervals do not permit a crossing under any parameter combination. Furthermore, between these periods, that is, middle Turonian to early Campanian (92.5–82.5 Ma), crossings would have been possible only for dinosaurs with substantial fat reserves (e.g., 10.36% fat mass or more) and intermediate to high BMRs (Figures 6, 7). However, the low RBRSD values (< 20%), even with the high fat requirements, make this interval an unlikely one for a successful crossing (Figures 8, 9). Therefore, the 97.5–77.5 Ma interval represents a time of very low crossing probability, at least via active swimming without current assistance. This information, in turn, can help refine previously suggested timings of faunal exchange that overlap with these intervals. Several studies, including Gheerbrant and Rage (2006) and Ősi et al. (2010), have proposed dispersals between Europe and Africa during this timeframe. However, given our results, the Albora route appears improbable during these highstand phases, and alternative explanations should be considered—such as gaps in the fossil record that delay the apparent timing of dispersal events, or dispersal occurring via other routes like Adria or proto‐Levant.

In conclusion, the Alboran route was probably traversable for most of the Cretaceous (i.e., during 13 out of 17 Cretaceous time slices) by swimming hadrosaurs and titanosaurs, if they had substantial fat mass (~10% body mass) and intermediate to high BMR. Nonetheless, even with conservative fat (~5% body mass), crossings were still possible for certain periods. When present, stepping stone islands in particular would have been important facilitators of dispersal via island‐hopping. The aforementioned timings are consistent with, and often help to refine, the conclusions of previous studies that considered the Albora route or some variant of it. While our study focuses on this single trans‐Tethyan route, the feasibility of alternative pathways remains an open question. A more detailed investigation is needed to explore the intricate nature of the trans‐Tethyan dispersal network across the European archipelago and assess the relative significance of each route, an undertaking we aim to pursue in future work.

### 4.3 Model Insights, Limitations, and Outlook

#### 4.3.1 Model Performance

Overall, the ENHYDROSS predictions conform closely to our expectations of a model that is performing relatively accurately: model outputs in terms of *U*_opt_ never stray out of the range recorded in the wild or experiments. At the same time, maximum estimated swimming durations and distances tend to be similar to, or greater than, empirical observations (presumably because the animals concerned had not reached their ultimate limits when observed) regardless of sensitivity test. Furthermore, when underestimates occur, there is strong evidence based on the passive drifting distances and times that these were the result of ocean currents with one exception (the polar bear under the “standing” BMR case—see Section 4.1.2). On one hand, this is good in terms of the validity and robustness of the model. On the other hand, this illustrates the complexity of attempting to make predictions regarding the swimming and aquatic dispersal abilities of terrestrial vertebrates in the wild. This is because of the strict assumptions underlying the model, including null‐thermogenesis and the restriction to continuous active swimming, without accounting for periods of inactivity (i.e., intermittent locomotion) or transport by ocean currents (passive drifting). The assumption of null‐thermogenesis is also the reason for the marked differences in COT_min_ estimates compared with those yielded by Meijaard's (2001) models. For a comparison between ENHYDROSS and Meijaard's (2001) models see Data S5.

#### 4.3.2 Understanding meta‐Optimality in Terms of Time‐Limits and Fat Reserves

We have created a meta‐approach that, out of a plausible range, identifies the BMR, or more precisely the sum *M*_b_ + *M*_T_ (and thus *U*_opt_ and COT_min_) that maximize the swimming distance, given a specified fat mass value and water‐deprivation time limit. In this sense, we have invented a meta‐optimality criterion for maximizing swimming distances when BMR is unknown for a particular taxon. Our results show that for the 14‐day time limit and under a fat mass of 5.18% body mass, our nonavian dinosaurs can swim the furthest at intermediate BMRs. These “optimal” BMRs are on par with the results produced by McNab's (2009, 2008) allometric equations for all birds, Struthioniformes, ratites, Paleognathae, and marsupials (Figure 3). On the other hand, for either a seven‐day limit or a fat mass of 10.36% body mass, the “optimal” BMRs are on par with the highest metabolic rates predicted by the mammalian scaling equations. We must emphasize for clarity that in neither case does this imply that our modeled nonavian dinosaurs necessarily had such metabolisms, but it gives a relative scale which one can use to identify how far an animal could have traveled in a limited time and under a given metabolic regime.

In the result section, we noted that the “optimal” BMR for the double fat curves in Figures 4 and 5 corresponded to twice the value of the respective “optimal” BMR point of the default fat (preferred parameters) curves for each dinosaur. This subtle, yet interesting point is actually trivial and a direct result of Equation (1) and one needs only to multiply “Available energy” and COT_min_ by 2 each to see them canceling out. This is interpreted as follows: for any animal with a certain fat mass and BMR at the “optimal” BMR or higher, to continue to be able to burn all its available energy stored in fat within its given time limit, if that fat mass is increased by *X* times, the animal's BMR must also increase by *X* times. Otherwise, it will succumb to the detrimental effects of water deprivation prior to exhausting its energy reserves for swimming.

Similarly, the reason why the “optimal” BMR of the 7‐day limit curve is halved compared to the 14‐day limit curve (Figures 4 and 5) is once again explained by examining Equations (1) and (2). If swimming time is halved, then the other side of the equation is also halved and when Equation (1) is then substituted in Equation (2) one can see that this is equivalent to multiplying BMR (i.e., *M*_b_) by a factor of 2 in the denominator. The interpretation derived from this is that for any animal with a set time limit (e.g., dehydration) to be able to burn all its available energy stored in fat, if that time limit is decreased by *X* times, the animal's BMR must increase by *X* times.

In summary, the shorter the time limit, the higher the “optimal” BMR point. Therefore, for relatively short time limits (e.g., from thirst or dehydration), animals with relatively high BMR (i.e., high *U*_opt_ and high COT_min_) will be advantaged because they will be able to cover more distance in a given time compared to animals that have the same characteristics (including fat mass) but lower BMRs. Cases where time limit is not an issue on the other hand, will benefit animals with lower metabolisms. Indeed, this is confirmed from the scarce data from the wild, particularly the ectothermic animals which can last for a prolonged time in the sea (Table 4). Having large amounts of fat is no longer an advantage when the animal cannot convert it to distance traveled within a limited time. On the other hand, being able to quickly convert as much energy as possible into distance traveled, is certainly advantageous and results in the highest swimming distances in such cases.

#### 4.3.3 Model Limitations and Prospects for Future Studies

With regards to the ENHYDROSS model itself, there are several points which could be refined in the future. The limitations of our model reflect its underlying assumptions and eliminating them would be a priority for advancing our model's capacities, applicability range and accuracy.

The first, and perhaps the most important of these assumptions, is that of null‐thermogenesis. The effects of even modest decreases in water temperature on an animal's heat loss cannot be underestimated, given the approximately 23‐fold higher thermal conductivity of water compared to air. This makes our assumption rather optimistic for the wild, at least with regard to endothermic animals. Similarly, whether complete thermal substitution is feasible and realistic with regard to low‐speed swimming by terrestrial animals in sea water at typical ocean temperatures is currently unknown because of the lack of empirical measurements. This in turn prevents us from verifying the veracity of our ENHYDROSS results in the absence of thermoneutral conditions, particularly in cases such as the polar bear (Section 4.1.2). Related to this is the scarcity of studies on the changes of BMR (*M*_b_) and thermogenesis (*M*_T_) or metabolism, in general, experienced by terrestrial animals (especially large ones) when immersed in water while resting and moving. Such data can be used to improve as well as validate our model. For example, just as Meijaard (2001) used the COT scaling equation by Williams (1999) by choosing the maximum 5.1× multiplier (range 2.4–5.1× COT for marine mammals) to represent the additional locomotory (or otherwise) cost for terrestrial mammal swimmers, so did we in an analogous manner with the 2.3 × BMR enhancement test. In effect, these are crude average approximations and, in our case, only useful for a comparison between the Meijaard and ENHYDROSS models. Being average approximations means that they cannot capture the differences in thermoregulation costs caused by species differing in shape, size, insulation, and physiology. Being crude approximations means that these results should be treated with caution because the optimal speed calculated in either model (Meijaard's and ENHYDROSS) is not really optimal using such ad hoc metabolic adjustments. This highlights the need for a next‐generation mechanistic model that explicitly accounts for thermoregulation outside null‐thermogenesis conditions, thereby allowing wider applicability and increased accuracy. Such a model would be of great value to conservation efforts for many animals undergoing long swims, seasonal migrations or dispersals to islands anywhere in the globe, from the poles to the tropics.

With regards to ectothermic animals, our model is less challenged by ocean surface temperature issues. For the crocodile and the tortoise, the ENHYDROSS model can be used without concern that the results will be changed dramatically by a future model, provided that environmental temperatures do not fall below a certain value that will cause these ectothermic animals to cease movement because of hypometabolism. Of course, such animals are affected by changing environmental temperatures and, much like endotherms, a day‐night cycle would need to be implemented to better capture how prolonged journeys in the sea affect their locomotion and pause of activity.

Concerning hydrodynamics, the drag coefficients were calculated using approximation formulae which, of course, are bound to deviate from the real values to some extent. In fact, the calculated *C*_d_ values for our terrestrial animals are of the same order of magnitude as those of fully aquatic species like dolphins: This is unlikely to reflect reality because the *C*_d_ of a dinosaur or an elephant will certainly be much higher than that of a streamlined aquatic species in the same Reynolds number domain. CFD studies *sensu* Gutarra et al. (2019), will be required to deduce exactly how much the results using such formulae for terrestrial animals differ from reality. Moreover, the propulsive and aerobic efficiencies used in ENHYDROSS are based on values for much smaller animals than those targeted here, and may well be different in larger taxa. However, the difference is unlikely to be significant enough to affect our results. We have varied these values within reasonable ranges and the resulting differences in our estimates are not significantly large (Figure 3; see also Data S3 and the ENHYDROSS core dataset available on Zenodo), although we did not test for all possible combinations because of their sheer number (Table S3.1). Any study on aquatic locomotion that can measure empirically the propulsive and/or aerobic efficiencies of large terrestrial paddling animals will reveal how much our chosen parameter values depart from reality.

Here, we have focused on terrestrial and semi‐aquatic animals for which trans‐oceanic dispersal was a topic of interest. Due to the scarce recorded data from the wild and the challenges in obtaining them, we could not include any measurements for the COT_min_ for validation. However, the novel Equations (8), (9) and (22), (23) for estimating COT_min_ are so fundamental and important in terms of application breath, they call for validation beyond our restricted domain of study. This could be achieved using large amounts of empirical data for aquatic animals (e.g., Hind and Gurney 1997; Videler and Nolet 1990; Williams 2022, 1999). Furthermore, our mechanistic framework can be adapted to marine organisms, for example, by modifying the optimality criteria (see Section 2.1.1) to account for behaviors such as stop‐overs, feeding en route, reproduction and so on, which can help improve already existing models (e.g., Braithwaite et al. 2015). Our novel equations could also be incorporated/adapted into models with broader scope and depth, improving or complementing certain aspects of them relating to *U*_opt_, COT_min_ and hence dispersal limits (Pirotta 2022 and references therein). When combined with Ecological Niche Models that predict potential habitat occupancy, this approach could provide a powerful tool for linking individual dispersal capabilities with population‐level distributions for marine taxa, opening new avenues for comparative studies of dispersal and range expansion under defined optimality criteria.

The mechanistic approach we used here for obtaining *U*_opt_ and COT_min_, traditionally confined to migration and locomotion studies, has now acquired a role in biogeographic research evaluating trans‐oceanic dispersal, and could be used synergistically with phylogenetic biogeographic methods for cross‐validation. In particular, the results from models such as ENHYDROSS could potentially inform decisions made in phylogenetic approaches such as the dispersal multiplier matrices used in BioGeoBEARS (Matzke 2013).

Moreover, our modeling approaches could be implemented in spatially explicit Agent‐Based models similar to those by Hertler et al. (2025, 2022). Such spatially explicit models can incorporate ocean currents and other palaeoclimatic conditions (e.g., winds, temperature). This could improve our ENHYDROSS predictions and might result in vastly different conclusions when, for example, strong ocean currents deflect swimming/drifting pathways away from the shortest distance between two landmasses.

## 5 Conclusions

We have presented a new energetic model that can estimate both optimal swimming speeds (*U*_opt_) and their corresponding minimum cost of transport (COT_min_), derived from fundamental principles; to our knowledge, this has not been done previously in aquatic locomotion studies. With the theoretically derived *U*_opt_ and COT_min_ equations and a number of empirically measured parameter values that are substituted in them, our model is then capable of calculating the maximum swimming distances and durations in any clade of vertebrates and in particular terrestrial ones, estimates that were urgently needed for modeling trans‐oceanic dispersal in biogeographic studies. Consequently, our model has, for the first time, formally linked a traditionally biogeographic concept with the realm of aquatic locomotion, through the optimal swimming speed and cost of transport equations and their underlying parameters.

We have applied the ENHYDROSS model to five extant animals, successfully predicting that, under certain model parameters and in some cases the influence of ocean currents, with the possible exception of the ostrich, these animals can swim long to extreme distances and for prolonged durations in line with empirical observations. This reinforces the idea that close relatives of these animals could have successfully dispersed across large bodies of water in the past, as hypothesized by other lines of evidence (e.g., Athanassiou et al. 2015; Caccone et al. 1999; Groh et al. 2023).

We have compared the results of ENHYDROSS with those generated by the Meijaard (2001) model, showing that when observational data exist for endothermic animals, both models produce results aligned with reality (albeit sometimes with certain adjustments, such as the presence of ocean currents). However, ENHYDROSS better captures dispersal potential in ectotherms, where Meijaard's (2001) model systematically underestimates due to its focus on mammals. While the relative accuracy of the two models, especially for endotherms, remains to be determined, ENHYDROSS is more generalizable and flexible, yielding biophysically justifiable results grounded in mechanistic, hydrodynamic, and physiological principles, and therefore offering greater explanatory power while aligning with observations. This is particularly true under the assumption of null‐thermogenesis. When this assumption does not hold, we demonstrate how a simple BMR enhancement based on the marine–terrestrial mammal difference can reduce dispersal limits for endothermic animals. Even under these conditions, ENHYDROSS COT_min_ estimates approach—but generally remain below—those of the previous model, suggesting Meijaard's (2001) approach may yield overly conservative estimates.

We have applied ENHYDROSS to two nonavian dinosaurs, a hadrosaur and a titanosaur, both belonging to lineages with putative long distance trans‐oceanic dispersing capabilities. Hadrosaurs displayed superior swimming efficiency in terms of optimal swimming speed (*U*_opt_) and cost of transport (COT_min_), albeit by a relatively small margin. Conversely, the titanosaur, being more massive and thus proportionally storing more fat, had the capacity to last longer and travel further, again albeit by a relatively small margin. However, under a 14‐day water deprivation time limit, neither dinosaur outperforms the other in all cases, as metabolic rate in combination with other hydrodynamic variables becomes critical factors. Furthermore, using simple distance measurements from an up‐to‐date paleogeographic model, we have shown that trans‐oceanic dispersal hypotheses involving Europe and Africa in the Cretaceous, particularly the early to middle Albian (107.5–112.5 Ma), are feasible with varying levels of support. In some cases, this feasibility is achieved even with relatively little fat (5.18% of body mass). Doubling fat reserves to 10.36% provides strong support for the feasibility of such dispersals, particularly during periods of lower sea levels such as the Late Cretaceous (72.5–67.5 Ma) when island hopping may have been possible. On the other hand, other intervals, such as 97.5–77.5 Ma, show weak to no support for trans‐oceanic crossings.

Our energetic model should be particularly useful for biogeographers, ecologists, paleobiologists, conservationists, and marine biologists, and it will be of best use when combined with case specific geographical, geological, and climatic information.

## Author Contributions

**Alexandros Pantelides:** conceptualization (lead), data curation (lead), formal analysis (equal), investigation (lead), methodology (lead), resources (lead), validation (lead), visualization (lead), writing – original draft (lead), writing – review and editing (equal). **Paul Upchurch:** conceptualization (lead), investigation (equal), methodology (equal), project administration (lead), resources (supporting), supervision (lead), validation (supporting), visualization (supporting), writing – original draft (equal), writing – review and editing (lead). **Philip D. Mannion:** conceptualization (supporting), funding acquisition (supporting), supervision (supporting), writing – review and editing (equal). **Elias Gravanis:** formal analysis (lead), methodology (equal). **Donald M. Henderson:** methodology (equal), software (lead), visualization (equal), writing – review and editing (supporting). **Phaedon Kyriakidis:** conceptualization (equal), methodology (supporting), project administration (lead), resources (supporting), supervision (lead), writing – review and editing (supporting).

## Funding

This work was supported by the Royal Society, UF160216, URF\R\221010.

## Conflicts of Interest

The authors declare no conflicts of interest.

## Supporting information


**Data S1:** ece373280‐sup‐0001‐SupplefileS1.pdf.

**Data S2:** ece373280‐sup‐0002‐SupplefileS2.pdf.

**Data S3:** ece373280‐sup‐0003‐SupplefileS3.pdf.

**Data S4:** ece373280‐sup‐0004‐SupplefileS4.pdf.

**Data S5:** ece373280‐sup‐0005‐SupplefileS5.pdf.

**Data S6:** ece373280‐sup‐0006‐SupplefileS6.pdf.

**Data S7:** ece373280‐sup‐0007‐SupplefileS7.pdf.

**Data S8:** ece373280‐sup‐0008‐SupplefileS8.pdf.

## Data Availability

All the required data are uploaded as Supporting Information.
