# Supplementary material for: ENHYDROSS: A New Mechanistic Model Supports the Trans‐Oceanic Dispersal Capability of Terrestrial Vertebrates
Source: Ecol Evol. 2026 Mar 30;16(4):e73280. doi: 10.1002/ece3.73280 (PMC13107292; doi:10.1002/ece3.73280)
Supplement: Supplementary file 1 — Data S1: ece373280‐sup‐0001‐SupplefileS1.pdf. [file ECE3-16-e73280-s003.pdf]

S1. Revisiting Meijaard's (2001) preliminary energetic model for swimming mammals

S1.1. The Meijaard (2001) model and its limitations

Here we will outline Meijaard's (2001) earlier model and explain why adjustments and additions are required. The study by Meijaard (2001) represents the first attempt to model constraints on trans-oceanic dispersal by combining organismal energetics with hydrodynamic principles. Essentially, Meijaard's (2001) modelling approach can be separated into four linked components, each related to a key equation. These are: 1) the implicit choice of an optimality criterion and the calculation of the associated speed of movement; 2) the calculation of the cost of transport (COT) based on that optimality criterion; 3) the calculation of the available energy reserves of the animal; and 4) the calculation of the maximum swimming distance and its corresponding duration.

For component 1, Meijaard (2001) modelled the energetics of swimming mammals based on the criterion of distance maximization (i.e. the optimum swimming speed is that which minimizes the organism's energy expenditure per unit distance, or in other words the cost of transport). In order to calculate this optimal speed, Meijaard (2001) first estimated the waterline length (L_w) of his target mammals, assuming it to be equal to snout-vent length. Using $L_{w,}$ he then calculated the hull speed (i.e. the theoretical maximum speed at which a hull body can travel on the surface of water by displacing the wave crest that propagates in front of it, as opposed to riding on top of it). The formula for the hull speed (V_{hull}) in ship engineering applies for the waterline length (L_w):

$$V_{hull} = \sqrt{\frac{gL_w}{2\pi}} \qquad \text{(S1)}$$

Optimal speed was calculated as 16.5% of hull speed, a value inferred from human and elephant observed velocities; hence the equation:

$$U_{opt} = 16.5\% \times V_{hull} \qquad \text{(S2)}$$

For component 2, Meijaard (2001) used the total (mass-specific) cost of transport scaling relationship developed by Williams (1999) based on marine and semi-aquatic mammals, multiplied by a factor of 5.51 to account for non-specialized semi-terrestrial and thus terrestrial swimmers, in Joules per kg per meter:

$$COT_{TOT} = 39.73\, m^{-0.29} \qquad \text{(S3)}$$

Note that in opting to use the concept of optimal speed, the Meijaard model implicitly assumes that Williams (1999) formula for the total cost of transport (COT_{TOT}) is equal to COT_{min}.

For component 3, Meijaard (2001) used two allometric equations derived by Prothero (1995) to estimate fat mass in two mammalian size classes, the primary energy source for long-distance movement. Prothero's (1995) allometric scaling relation for fat mass in small animals ranging from 4 g to 21.5 kg is given by:

$$\log F = \log m - 1.207 \Rightarrow F = \frac{m}{10^{1.207}} = \frac{m}{16.106...} \qquad \text{(S4)}$$

And for large animals between 21.5 kg – 10000 kg the relation is:

$$\log F = \log m - 1.146 \Rightarrow F = \frac{m}{10^{1.146}} = \frac{m}{13.995\ldots} \quad \text{(S5)}$$

where F is the fat mass and m is the body mass of the animal both measured in kilograms.

Meijaard (2001) then converted the calculated fat masses to energy by multiplying by the energy content of fat (39 MJ/kg) and a conversion efficiency of 23%, assuming that all fuel used by the swimming mammals relied exclusively on that source.

The remaining calculations (component 4) were estimates of the distance the animal can travel before running out of energy, and the time required for such a journey given an optimal speed, minimum cost of transport, and fuel store. These are straight-forward equations:

$$Max\ sea\ traveling\ time = \frac{Available\ energy}{Total\ Metabolic\ Rate} \quad \text{(S6)}$$

$$Max\ Distance\ travelled = Max\ Time\ travelled \times U_{opt} \quad \text{(S7)}$$

However, because the Meijaard's (2001) model does not incorporate total metabolic rate (W) directly (as shown in eq. S6), an intermediate step must be taken to estimate it. Specifically, one has to multiply COT with speed and mass (Meijaard, 2001):

$$Total\ Metabolic\ Rate = COT_{TOT} \times\ m \times\ U_{opt} \quad \text{(S8)}$$

Despite its apparent simplicity, Meijaard's (2001) model had predictive power, with its results apparently well in accord with the observed swimming distances for a number of mammals. However, Meijaard (2001), regarded the model as preliminary for several reasons, including: 1) it overestimated the swimming distances of smaller animals; 2) it did not account for energy obtained from protein and carbohydrate fuel (although within the paper he remarked that for long distance movement most fuel comes from oxidation of lipids and thus this would be a minor omission); 3) it ignored the effects of sea currents and waves; and 4) it was not tailored for individual anatomical, biomechanical and physiological characteristics (for example, he noted that predictions of fat mass from body weight alone may be inaccurate). Additionally, by design, Meijaard's (2001) model only dealt with mammals, substantially limiting its applicability to the wide array of organisms that have potentially participated in transoceanic dispersal. These issues would be reason enough to revisit Meijaard's (2001) model and attempt refinements and extension; however, during this process we have come across further problems that need to be addressed.

S1.2 Additional problems with Meijaard's model

First, the way the optimal swimming speed was calculated in Meijaard's (2001) model (component 1) is problematic. The assumption that optimal swimming speed equals 16.5% of the hull speed was based solely on two data points: one for humans and the other for elephants (the latter based on an average estimate (0.43 m/s) obtained by recording the same individual in two consecutive swims (Johnson, 1980 and references therein). There are good reasons to expect that optimal swimming speed will vary depending on a number of factors aside from waterline length (L_W), such as metabolism, hydrodynamic drag (a factor that depends on the animals' shape and size), and locomotory style (e.g. paddling or undulatory swimming). Unfortunately, the impact of these other factors on optimal swimming speed

cannot be assumed to be negligible. Thus, humans and elephants may not be representative of other mammals, let alone non-mammalian vertebrates, and so the universality of the 16.5% fraction of the hull speed is questionable. Furthermore, the assumption that head-body (snout-vent) length equals waterline length (L_w) does not hold universally. Meijaard (2001) justified the use of snout-vent length as a proxy for L_w on the basis of evidence from a single study on Australian water rats (Fish and Baudinette, 1999). The majority of mammals have relatively short necks and tails that contribute little to their length, so their snout-vent length is almost identical to their entire body length (L_b), and thus the assumption that L_b equals L_W may indeed hold in many cases, e.g. in both a giraffe (Henderson and Naish, 2010: fig. 2) and a polar bear (e.g. Pagano et al., 2019: fig. 2). However, other examples demonstrate the non-universality of this relationship. The waterline length for a floating 4.38 m long elephant is approximately 2.6 m (see Supplementary File S6: Figure S6.1.). This is a particular concern for the accuracy of the studies by Meijaard (2001) and (Hertler et al., 2025, 2022), given the dependence of the hull speed correction factor on the elephant in the former and the focus on its extinct relative *Stegodon* in the latter. Similarly, the waterline length is shorter than snout-vent length in horses (Henderson and Naish, 2010: fig. 2: note that the middle of the horse's back lies below the surface of the water), hadrosaurid dinosaurs (Henderson, 2014: fig. 27.3), ceratopsian dinosaurs (Henderson, 2014: fig. 27.5, 27.6), and the ankylosaurian dinosaur *Euoplocephalus* (Mallon et al., 2018: fig. 3). In contrast to *Euoplocephalus*, snout-vent length is estimated to be very similar to L_W in the ankylosaur *Sauropelta* (Mallon et al., 2018: fig.3), illustrating how the relationship between these two parameters can vary even within a clade of closely related taxa with similar body plans. Finally, for sauropod dinosaurs, much depends on the assumed degree of neck elevation. If the neck was elevated out of the water, then L_W would have been less or equal to snout-vent length; by contrast, if the entire length of the necks was floating, then L_W would be greater than or equal to the snout-vent length, depending on the taxon and the position of the tail (Henderson, 2004: fig. 2).

A second problem concerns the linkage between optimal swimming speed and COT. The latter is defined as the amount of metabolic energy required to travel a certain distance, or for mass-specific COT, the energy needed to move a unit mass over a unit distance. However, the specific definition is not crucial here. What is important is that COT, by either definition, is influenced by the speed of locomotion. This is because the metabolic energy expended is utilized for (among other things such as basal metabolism) the energy cost of locomotion needed to achieve a certain speed and consequently move. With Meijaard's (2001) approach, this coupling of COT and speed is entirely ignored because the calculation of the 16.5% of the hull speed has nothing to do with the COT obtained from Williams' (1999) empirical allometric equation. The latter equation only depends on the mass of an animal and does not take into account the animal's speed measured from the fraction of the hull speed (or any other parameter for that matter). In fact, under Meijaard's (2001) approach, the calculation of the speed has little to do with the optimality criterion. The fraction of the hull speed was simply a consistent way to calculate swimming speeds for various mammals that seemed close to what could be universally assumed to be approximately optimal. In effect, the optimality criterion was enforced with component 2, i.e. the selection of COT_{min} to begin with, since this selection was grounded on a relationship derived by Williams (1999) based on more data from animals that presumably were swimming at or close to an optimal speed. The speed calculated by Meijaard (2001) was just termed 'optimal' – in fact, this had zero effect on the swimming distances estimated provided the COT_{min} was known (see main text eq. 1 and section 2.1.2 for further explanation). Only the swimming time was affected by the

estimated speed, and, arguably not very much at that, given that the low speeds involved in such swimming activity would not have differed by much if another speed equation had been used that was close to that range (see main text eq. 2 and section 2.1.2 for further explanation; see also the Results of the current study). Thus, when using Meijaard's (2001) model, one could argue that the swimming speed calculation is not important because it has only a minor impact on estimated swimming duration and distance. However, such a conclusion reflects a problem with Meijaard's (2001) approach, rather than providing a justification for downplaying the impact of swimming speed. First, while COT_{min} alone can be used to estimate maximum swimming distances, we cannot currently calculate it together with swimming speed without experimental data or allometric equations. This is not a critique of Meijaard's (2001) methodology *per se*, rather this is a knowledge gap. Second, merely adjusting the COT from marine mammals via a single multiplier to approximate a value for any terrestrial species based solely on input mass, as suggested by Williams (1999) and adopted by Meijaard (2001), would in all likelihood result in inaccuracies. Meijaard (2001) conservatively used a 5.1 x COT multiplier based on the range of values (2.4–5.1) suggested by Williams (1999). Although this was a reasonable approach as a first attempt, the difference between a 2.4 x and 5.1 x multiplier for the COT will translate into a marked difference in the estimated swimming distances. Because COT depends on speed, and the latter depends on many other parameters (e.g. locomotory mode, hydrodynamic drag, and metabolism – see sections 2.1.3 and 2.1.6), we cannot be confident that COT will be the same for all terrestrial vertebrates. Thus, in terms of both definition and optimization, calculating COT without accounting for the speed that corresponds to that specific COT is erroneous and potentially leads to inaccurate results.

A third problem concerns component 3, where a calculation of the fat mass (i.e. the energy reserves available to each animal) is required. First, as noted above, the equations presented by Prothero (1995) apply solely to mammals, and thus other vertebrates probably have different scaling relationships. Second, Prothero's (1995) equations were derived from a regression line with large dispersion (73% mean percentage deviation), which warrants caution regarding its usage (as acknowledged by both Prothero [1995] and Meijaard [2001]). This is to be expected because interstitial and depot fat is highly variable across both individuals and species, and is closely tied to nutrition (Pond, 1978 and references therein). Notably, body fat mass often comprises over half of an animal's live weight, and yet it can be reduced to almost 0% under some circumstances (Pond, 1978 and references therein). Third, estimating fat mass from an allometric equation that spans nearly three orders of magnitude of body mass and applies to all mammals may be inadvisable. Although Prothero's (1995) equations provide a reasonable estimate for the average mammal's fat mass, they cannot capture variations among clades resulting from diverse lifestyles, diets, and individual factors such as life stage or reproductive cycle. In addition, the exponent confidence intervals of these equations were not used by Meijaard (2001), something that could potentially have had a substantial impact on model predictions in terms of swimming distance and duration. Thus, relying on a single fat mass value may not sufficiently account for the diversity within species or individuals, potentially leading to inaccurate estimation of the limits on dispersal distance.

The overall message from the above is that using the model by Meijaard (2001) poses several problems that require appropriate amendments and modifications.

References

Fish, F.E., Baudinette, R.V., 1999. Energetics of locomotion by the Australian water rat (*Hydromys chrysogaster*): a comparison of swimming and running in a semi-aquatic mammal. Journal of Experimental Biology 202, 353–363. https://doi.org/10.1242/jeb.202.4.353

Henderson, D.M., 2014. Duck Soup: The Floating Fates of Hadrosaurs and Ceratopsians at Dinosaur Provincial Park, in: Hadrosaurs, Life of the Past. Indiana University Press, Bloomington, pp. 459–466.

Henderson, D.M., 2004. Tipsy punters: sauropod dinosaur pneumaticity, buoyancy and aquatic habits. Biol. Lett. 271, S180–S183. https://doi.org/10.1098/rsbl.2003.0136

Henderson, D.M., Naish, D., 2010. Predicting the buoyancy, equilibrium and potential swimming ability of giraffes by computational analysis. Journal of Theoretical Biology 265, 151–159. https://doi.org/10.1016/j.jtbi.2010.04.007

Hertler, C., Reschke, J.O., Hölzchen, E., Anwar, I.P., Puspaningrum, M.R., Büscher, N., Ngetich, E.K., 2022. SEAcross ABM v1.0 (1.0). https://doi.org/10.5281/ZENODO.6833780

Hertler, C., Van Der Geer, A.A.E., Puspaningrum, M.R., Reschke, J.-O., Anwar, I.P., Hölzchen, E., 2025. Stegodon SEA-crossing: Swim, Shrink, and Disperse. Earth History and Biodiversity 100026. https://doi.org/10.1016/j.hisbio.2025.100026

Johnson, D.L., 1980. Problems in the Land Vertebrate Zoogeography of Certain Islands and the Swimming Powers of Elephants. Journal of Biogeography 7, 383. https://doi.org/10.2307/2844657

Mallon, J.C., Henderson, D.M., McDonough, C.M., Loughry, W.J., 2018. A “bloat-and-float” taphonomic model best explains the upside-down preservation of ankylosaurs. Palaeogeography, Palaeoclimatology, Palaeoecology 497, 117–127. https://doi.org/10.1016/j.palaeo.2018.02.010

Meijaard, E., 2001. Successful sea-crossings by land mammals; a matter of luck, and a big body. A preliminary and simplified model. Geol. Res. Dev. Centre, Spec. Publ 87–92.

Pagano, A.M., Cutting, A., Nicassio-Hiskey, N., Hash, A., Williams, T.M., 2019. Energetic costs of aquatic locomotion in a subadult polar bear. Mar. Mam. Sci. 35, 649–659. https://doi.org/10.1111/mms.12556

Pond, C.M., 1978. Morphological Aspects and the Ecological and Mechanical Consequences of Fat Deposition in Wild Vertebrates. Annu. Rev. Ecol. Syst. 9, 519–570. https://doi.org/10.1146/annurev.es.09.110178.002511

Prothero, J., 1995. Bone and fat as a function of body weight in adult mammals. Comparative Biochemistry and Physiology Part A: Physiology 111, 633–639. https://doi.org/10.1016/0300-9629(95)00050-H

Williams, T.M., 1999. The evolution of cost efficient swimming in marine mammals: limits to energetic optimization. Philos Trans R Soc Lond B Biol Sci 354, 193–201. https://doi.org/10.1098/rstb.1999.0371
