# Supplementary material for: ENHYDROSS: A New Mechanistic Model Supports the Trans‐Oceanic Dispersal Capability of Terrestrial Vertebrates
Source: Ecol Evol. 2026 Mar 30;16(4):e73280. doi: 10.1002/ece3.73280 (PMC13107292; doi:10.1002/ece3.73280)
Supplement: Supplementary file 2 — Data S2: ece373280‐sup‐0002‐SupplefileS2.pdf. [file ECE3-16-e73280-s006.pdf]

S2. Equations

S2.1. Derivation of the ENHYDROSS model equations (6), (8), (19) and (21)

In this section we describe step-by-step how we derived equations (6), (8), (19) and (21) of the main text.

For equation (6) we start by stating the necessary equations (4 and 5) from the main text for clarity. First, we state the equation for COT_{TOT}:

$$COT_{TOT} = \frac{M_b+M_T+M_L}{U} \tag{4}$$

Where U is the swimming speed in m/s, M_b is the BMR, M_T is the metabolic cost of thermogenesis, if one exists, and M_L is the metabolic cost of locomotion all measured in Watts. The latter is defined as:

$$M_L = \frac{0.5\rho\lambda SC_d}{\varepsilon_p\varepsilon_A}U^3 \tag{5}$$

Where ρ is the density of water, S is the surface area of the animal (here we mean either the submerged frontal or the entire wetted area of the animal), C_d is the drag coefficient, ε_p and ε_A are the propulsive and aerobic efficiencies respectively, U is the swimming speed (not necessarily the optimal speed) and λ is the active/passive drag ratio.

Here, we are interested in the special case where $U=U_{opt}$ which is where $COT_{TOT}=COT_{min}$ by our definition of optimality. The function COT_{TOT} has a minimum where its derivative is zero. Thus, if we take the derivative of equation (4) we obtain the equation for the U_{opt} as demonstrated below:

$$(COT_{TOT})' = 0 = \frac{d}{dU}\left(\frac{M_b+M_T+M_L}{U_{opt}}\right) = -\left(\frac{M_b+M_T}{U_{opt}^2}\right) + \frac{\rho\lambda SC_dU}{\varepsilon_p\varepsilon_A}$$

$$\Rightarrow \left(\frac{M_b+M_T}{U_{opt}^3}\right) = \frac{\rho\lambda SC_d}{\varepsilon_p\varepsilon_A}$$

$$\Rightarrow U_{opt} = \left(\frac{\varepsilon_p\varepsilon_A(M_b+M_T)}{\rho\lambda SC_d}\right)^{1/3} \tag{6}$$

Regarding the U_{opt} equation (main text: eq. 6 and 7), we note that in some cases (Blanco, 2023; Gutarra and Rahman, 2022; Motani, 2002; Villamil et al., 2016) there is a 0.5 in the denominator inside the parenthesis, which can be traced back to Motani (2002). It is unclear to us as to how Motani (2002) derived this formula because they state that this was done by taking the derivative of equation (4). However, if one takes the derivative of equation (4), the 0.5 of the denominator in the *U_{opt}* equations (6) and (7) is eliminated, as demonstrated above.

Back-substituting equations (5+6) into equation (4) we obtain the COT_{min} equation (8) in the following way:

$$COT_{min} = \frac{M_b + M_T}{\left(\frac{\varepsilon_p\varepsilon_A(M_b + M_T)}{\rho\lambda SC_d}\right)^{1/3}} + \frac{1}{2}\frac{\rho\lambda SC_d}{\varepsilon_p\varepsilon_A}\left(\frac{\varepsilon_p\varepsilon_A(M_b + M_T)}{\rho\lambda SC_d}\right)^{2/3}$$

$$= (M_b + M_T)\left(\frac{\rho\lambda SC_d}{\varepsilon_p\varepsilon_A(M_b + M_T)}\right)^{1/3} + \frac{1}{2}\frac{\rho\lambda SC_d}{\varepsilon_p\varepsilon_A}\left(\frac{\varepsilon_p\varepsilon_A(M_b + M_T)}{\rho\lambda SC_d}\right)^{2/3}$$

$$= \left(\frac{\rho\lambda SC_d}{\varepsilon_p\varepsilon_A(M_b + M_T)}(M_b + M_T)^3\right)^{1/3} + \frac{1}{2}\left(\left(\frac{\rho\lambda SC_d}{\varepsilon_p\varepsilon_A}\frac{(M_b + M_T)}{(M_b + M_T)}\right)^3\left(\frac{\varepsilon_p\varepsilon_A(M_b + M_T)}{\rho\lambda SC_d}\right)^2\right)^{1/3}$$

$$= \left(\frac{\rho\lambda SC_d(M_b + M_T)^2}{\varepsilon_p\varepsilon_A}\right)^{1/3} + \frac{1}{2}\left((M_b + M_T)^3\left(\frac{\rho\lambda SC_d}{\varepsilon_p\varepsilon_A}\frac{1}{(M_b + M_T)}\right)^3\left(\frac{\varepsilon_p\varepsilon_A(M_b + M_T)}{\rho\lambda SC_d}\right)^2\right)^{1/3}$$

$$= \left(\frac{\rho\lambda SC_d(M_b + M_T)^2}{\varepsilon_p\varepsilon_A}\right)^{1/3} + \frac{1}{2}\left((M_b + M_T)^3\frac{\rho\lambda SC_d}{\varepsilon_p\varepsilon_A(M_b + M_T)}\right)^{1/3}$$

$$= \left(\frac{\rho\lambda SC_d(M_b + M_T)^2}{\varepsilon_p\varepsilon_A}\right)^{1/3} + \frac{1}{2}\left(\frac{\rho\lambda SC_d(M_b + M_T)^2}{\varepsilon_p\varepsilon_A}\right)^{1/3}$$

$$= \frac{3}{2}\left(\frac{\rho\lambda SC_d(M_b + M_T)^2}{\varepsilon_p\varepsilon_A}\right)^{1/3}$$

$$= \frac{3}{2}\left(\frac{\rho\lambda SC_d(M_b + M_T)^2}{\varepsilon_p\varepsilon_A}\frac{(M_b + M_T)}{(M_b + M_T)}\right)^{1/3}$$

$$= \frac{3}{2}\left(\frac{\rho\lambda SC_d(M_b + M_T)^3}{\varepsilon_p\varepsilon_A(M_b + M_T)}\right)^{1/3}$$

$$= \frac{3}{2}\left(\frac{\rho\lambda SC_d}{\varepsilon_p\varepsilon_A(M_b + M_T)}\right)^{1/3}\left((M_b + M_T)^3\right)^{1/3}$$

$$= \frac{3}{2}\frac{(M_b+M_T)}{U_{opt}} \qquad (8)$$

To obtain equation (19) let us first state equation (18) for the total cost of transport (COT_{TOT}) for clarity:

$$COT_{TOT} = \frac{M_b+M_T}{U} + \frac{1}{2}\frac{\rho\lambda S\widehat{C_d}}{\varepsilon_p\varepsilon_A}U^{9/5} \qquad (18)$$

By taking the derivative of this expression and setting $COT_{TOT} = 0$ we solve for U as we did previously and we once again obtain U_{opt}, but this time modified so that it does not contain an extra U term on the right side of the equation (thus avoiding the circularity - see main text section 2.1.6):

$$(COT_{TOT})' = 0 = \frac{d}{dU}\left(\frac{M_b + M_T + M_L}{U_{opt}}\right) = -\left(\frac{M_b + M_T}{U_{opt}^2}\right) + \frac{1}{2}\left(\frac{9}{5}\right)\frac{\rho\lambda S\widehat{C_d}}{\varepsilon_p\varepsilon_A}U_{opt}^{4/5}$$

$$\Rightarrow \left(\frac{M_b+M_T}{U_{opt}^{(2+\frac{4}{5})}}\right) = \frac{9}{10}\frac{\rho\lambda S\widehat{C_d}}{\varepsilon_p\varepsilon_A}$$

$$\Rightarrow \left(\frac{M_b+M_T}{U_{opt}^{(14/5)}}\right) = \frac{9}{10}\frac{\rho\lambda S\widehat{C_d}}{\varepsilon_p\varepsilon_A}$$

$$\Rightarrow \quad U_{opt} = \left(\frac{10}{9}\frac{\varepsilon_p \varepsilon_A (M_b+M_T)}{\rho\lambda S\widehat{C_d}}\right)^{5/14} \tag{19}$$

Back-substituting equation 19 to equation 18 we obtain equation 21 in the following way:

$$COT_{TOT} = \frac{M_b+M_T}{U} + \frac{1}{2}\frac{\rho\lambda S\widehat{C_d}}{\varepsilon_p\varepsilon_A}U^{9/5} \tag{18}$$

$$\Rightarrow \quad COT_{min} = \frac{M_b+M_T}{\left(\frac{10}{9}\frac{\varepsilon_p\varepsilon_A(M_b+M_T)}{\rho\lambda S\widehat{C_d}}\right)^{5/14}} + \frac{1}{2}\frac{\rho\lambda S\widehat{C_d}}{\varepsilon_p\varepsilon_A}\left(\frac{10}{9}\frac{\varepsilon_p\varepsilon_A(M_b+M_T)}{\rho\lambda S\widehat{C_d}}\right)^{\frac{5}{14}\times\frac{9}{5}}$$

$$= (M_b+M_T)\left(\frac{9}{10}\frac{\rho\lambda S\widehat{C_d}}{\varepsilon_p\varepsilon_A(M_b+M_T)}\right)^{5/14} + \frac{1}{2}\left(\frac{\rho\lambda S\widehat{C_d}}{\varepsilon_p\varepsilon_A}\right)^{14/14}\left(\frac{10}{9}\frac{\varepsilon_p\varepsilon_A(M_b+M_T)}{\rho\lambda S\widehat{C_d}}\right)^{9/14}$$

$$= (M_b+M_T)\left(\frac{9}{10}\frac{\rho\lambda S\widehat{C_d}}{\varepsilon_p\varepsilon_A(M_b+M_T)}\right)^{5/14}\left(1+\frac{1}{2}\left(\frac{9}{10}\right)^{-5/14}\left(\frac{10}{9}\right)^{9/14}\frac{1}{(M_b+M_T)^{9/14}}\left(\frac{\rho\lambda S\widehat{C_d}}{\varepsilon_p\varepsilon_A}\right)^{9/14}\left(\frac{\varepsilon_p\varepsilon_A(M_b+M_T)}{\rho\lambda S\widehat{C_d}}\right)^{9/14}\right)$$

$$= (M_b+M_T)\left(\frac{9}{10}\frac{\rho\lambda S\widehat{C_d}}{\varepsilon_p\varepsilon_A(M_b+M_T)}\right)^{5/14}\left(1+\frac{1}{2}\left(\frac{10}{9}\right)^{5/14}\left(\frac{10}{9}\right)^{9/14}\right)$$

$$= (M_b+M_T)\left(\frac{9}{10}\frac{\rho\lambda S\widehat{C_d}}{\varepsilon_p\varepsilon_A(M_b+M_T)}\right)^{5/14}\left(1+\frac{1}{2}\left(\frac{10}{9}\right)\right)$$

$$= (M_b+M_T)\left(\frac{9}{10}\frac{\rho\lambda S\widehat{C_d}}{\varepsilon_p\varepsilon_A(M_b+M_T)}\right)^{5/14}\left(\frac{14}{9}\right)$$

$$= \frac{14}{9}\frac{(M_b+M_T)}{U_{opt}} \tag{21}$$

S2.2. Derivation of the BRSD equations (24) (25) and (26)

To obtain equations (24-26) (see section 2.8) we need to rework equations (1), (2), (19) and (21). Writing equations (1-2) again for convenience:

$$Max\ swimming\ distance = \frac{Available\ energy}{COT_{min}} \tag{1}$$

$$Swimming\ time = \frac{Max\ swimming\ distance}{U_{opt}} \tag{2}$$

If we then substitute equations (19) and (21) into equation (1):

$$Gap\ distance := Max\ swimming\ distance = \frac{Available\ energy}{COT_{min}}$$

$$\Rightarrow \quad Gap\ distance = \frac{Available\ energy}{\frac{14}{9}\frac{(M_b+M_T)}{\left(\frac{10}{9}\frac{\varepsilon_p\varepsilon_A(M_b+M_T)}{\rho\lambda S\widehat{C_d}}\right)^{5/14}}}$$

$$\Rightarrow \quad (M_b+M_T) = \frac{9}{14}\frac{Available\ energy}{Gap\ distance}\left(\frac{10}{9}\frac{\varepsilon_p\varepsilon_A}{\rho\lambda S\widehat{C_d}}\right)^{5/14}(M_b+M_T)^{5/14}$$

$$\Rightarrow \quad (M_b+M_T)^{9/14} = \frac{9}{14}\frac{Available\ energy}{Gap\ distance}\left(\frac{10}{9}\frac{\varepsilon_p\varepsilon_A}{\rho\lambda S\widehat{C_d}}\right)^{5/14}$$

$$\Rightarrow \qquad (M_b + M_T) = \left[\frac{9}{14}\frac{Available\ energy}{Gap\ distance}\left(\frac{10}{9}\frac{\varepsilon_p\varepsilon_A}{\rho\lambda S\widehat{C_d}}\right)^{5/14}\right]^{14/9}$$

$$\Rightarrow \qquad (M_b + M_T) = \left(\frac{9}{14}\frac{Available\ energy}{Gap\ distance}\right)^{14/9}\left(\frac{10}{9}\frac{\varepsilon_p\varepsilon_A}{\rho\lambda S\widehat{C_d}}\right)^{5/9}$$

Where $M_b + M_T$ is equal to the maximum BMR of the BRSD for a given distance, the equation can be written in a simpler form:

$$BMR_{max} = \left(\frac{9}{14}\frac{Available\ energy}{Gap\ distance}\right)^{14/9}\left(\frac{10}{9}\frac{\varepsilon_p\varepsilon_A}{\rho\lambda S\widehat{C_d}}\right)^{5/9}$$

By including the conversion units in order to be able to use kilometers for distance directly, the equation then becomes equation (24):

$$BMR_{max} = \left(\frac{9}{14\times1000}\frac{Available\ energy}{Gap\ distance}\right)^{14/9}\left(\frac{10}{9}\frac{\varepsilon_p\varepsilon_A}{\rho\lambda S\widehat{C_d}}\right)^{5/9} \qquad (24)$$

The above equation will result in the BMR necessary for an animal to be able to cross a given gap distance, without any time limit. However, when a time limit is applied, this would not work for BMRs below the 'optimal' BMR. For the latter situation, we need another equation that contains the time limit parameter. As it happens, U_{opt} can be written as max swimming distance under a time limit divided by that time limit. Hence, taking the latter and equating with equation (19) we get:

$$U_{opt} = \left(\frac{10}{9}\frac{\varepsilon_p\varepsilon_A(M_b + M_T)}{\rho\lambda S\widehat{C_d}}\right)^{5/14} = \frac{Max\ swimming\ distance_{\ Time\ Limit}}{Time\ Limit} = \frac{Gap\ distance}{Time\ Limit}$$

$$\Rightarrow \qquad (M_b + M_T)^{5/14} = \frac{Gap\ distance}{Time\ Limit} \times \left(\frac{9}{10}\frac{\rho\lambda S\widehat{C_d}}{\varepsilon_p\varepsilon_A}\right)^{5/14}$$

$$\Rightarrow \qquad (M_b + M_T) = \left(\frac{Gap\ distance}{Time\ Limit}\right)^{14/5} \times \left(\frac{9}{10}\frac{\rho\lambda S\widehat{C_d}}{\varepsilon_p\varepsilon_A}\right)$$

Once again, where $M_b + M_T$ is equal to the lower BMR of the BRSD (see Supplementary File S7 for more information about the term definition) for a given distance, the equation can be written in a simpler form as:

$$BMR_{min} = \left(\frac{Gap\ distance}{Time\ Limit}\right)^{14/5} \times \left(\frac{9}{10}\frac{\rho\lambda S\widehat{C_d}}{\varepsilon_p\varepsilon_A}\right)$$

By including the conversion units in order to be able to substitute kilometers for distance, the equation then becomes equation (25):

$$BMR_{min} = \left(\frac{1000\times Gap\ distance}{24\times3600\times Time\ Limit}\right)^{14/5} \times \left(\frac{9}{10}\frac{\rho\lambda S\widehat{C_d}}{\varepsilon_p\varepsilon_A}\right) \qquad (25)$$

For equation (26) we just need to equate equations (24) and (25) and solve for distance:

$$BMR_{min} = BMR_{max}$$

$$\Rightarrow \quad \left(\frac{1000\times Gap\ distance}{24\times3600\times Time\ Limit}\right)^{14/5} \times \left(\frac{9}{10}\frac{\rho\lambda S\widehat{C_d}}{\varepsilon_p\varepsilon_A}\right) = \left(\frac{9}{14\times1000}\frac{Available\ energy}{Gap\ distance}\right)^{14/9}\left(\frac{10}{9}\frac{\varepsilon_p\varepsilon_A}{\rho\lambda S\widehat{C_d}}\right)^{5/9}$$

$$\Rightarrow (1000 \times Gap\ distance)^{14/5} \times (Gap\ distance)^{14/9} = (24 \times 3600 \times Time\ Limit)^{14/5} \times \left(\frac{9}{14\times1000} Available\ energy\right)^{14/9} \left(\frac{10}{9}\frac{\varepsilon_p\varepsilon_A}{\rho\lambda S\widehat{C_d}}\right)^{5/9} \left(\frac{9}{10}\frac{\rho\lambda S\widehat{C_d}}{\varepsilon_p\varepsilon_A}\right)^{-1}$$

$$\Rightarrow (Gap\ distance)^{196/45} = \left(\frac{24\times3600\times Time\ Limit}{1000}\right)^{14/5} \times \left(\frac{9}{14\times1000} Available\ energy\right)^{14/9} \left(\frac{10}{9}\frac{\varepsilon_p\varepsilon_A}{\rho\lambda S\widehat{C_d}}\right)^{14/9}$$

$$\Rightarrow Gap\ distance = \left[\left(\frac{1}{14\times100}\frac{Available\ Energy\times\varepsilon_A\varepsilon_p}{\rho\lambda S\widehat{C_d}}\right)^{14/9} \times \left(\frac{24*3600}{1000} \times Time\ Limit\right)^{14/5}\right]^{45/196}$$

$$\Rightarrow Gap\ distance = \left(\frac{1}{14\times100}\frac{Available\ Energy\times\varepsilon_A\varepsilon_p}{\rho\lambda S\widehat{C_d}}\right)^{5/14} \times \left(\frac{24*3600}{1000} \times Time\ Limit\right)^{9/14} \quad (26)$$

References

Blanco, R.E., 2023. *Tyrannosaurus rex* runs again: a theoretical analysis of the hypothesis that full-grown large theropods had a locomotory advantage to hunt in a shallow-water environment. Zoological Journal of the Linnean Society 198, 202–219. https://doi.org/10.1093/zoolinnean/zlac104

Gutarra, S., Rahman, I.A., 2022. The locomotion of extinct secondarily aquatic tetrapods. Biological Reviews 97, 67–98. https://doi.org/10.1111/brv.12790

Motani, R., 2002. Swimming speed estimation of extinct marine reptiles: energetic approach revisited. Paleobiology 28, 251–262. https://doi.org/10.1666/0094-8373(2002)028<0251:SSEOEM>2.0.CO;2

Villamil, J., Demarco, P.N., Meneghel, M., Blanco, R.E., Jones, W., Rinderknecht, A., Laurin, M., Piñeiro, G., 2016. Optimal swimming speed estimates in the Early Permian mesosaurid *Mesosaurus tenuidens* (Gervais 1865) from Uruguay. Historical Biology 28, 963–971. https://doi.org/10.1080/08912963.2015.1075018
