# Supplementary material for: ENHYDROSS: A New Mechanistic Model Supports the Trans‐Oceanic Dispersal Capability of Terrestrial Vertebrates
Source: Ecol Evol. 2026 Mar 30;16(4):e73280. doi: 10.1002/ece3.73280 (PMC13107292; doi:10.1002/ece3.73280)
Supplement: Supplementary file 3 — Data S3: ece373280‐sup‐0003‐SupplefileS3.pdf. [file ECE3-16-e73280-s008.pdf]

S3. Hydrodynamic Parameters

S3.1. Aerobic efficiency

The aerobic efficiency (ε_A) of muscles or the whole animal, measures the proportion of biochemical energy derived from aerobic metabolism that is converted into mechanical work during muscular activity. Using our terminology, ε_A shows how much of the aerobic metabolic power allocated to locomotion (M_L) is converted into mechanical power ($=\varepsilon_A*M_L$) (main text: Figure 1). In mammals, ε_A ranges from 0.24 to 0.33, and in vertebrates more broadly from 0.2 to 0.4 (Lovelace et al., 2020; Smith et al., 2005). Additionally, studies on rats have shown that the efficiency peaks at intermediate exercise stress (Gibbs and Gibson, 1972). Hind and Gurney (1997) and Motani (2002) utilized a modified formula for ε_A derived from Gibbs and Gibson's (1972) results. Specifically, Hind and Gurney (1997) set the peak efficiency calculated by Gibbs and Gibson (1972) for rats to match the intermediate velocity (~5m/s) of the former's studied animals (seals), producing a modified velocity-dependent relationship:

$\varepsilon_A=0.0679 + 0.4411\,(U/5)^3 - 0.422\,(U/5)^6$, where U is the swimming speed.

Thus, for speeds lower than 1−2 m/s, Hind and Gurney's (1997) ε_A estimated values would approximate 0.07 which is an order of magnitude smaller compared to the 0.2–0.4 range mentioned above (Lovelace et al., 2020; Smith et al., 2005). This agrees with Fish (1996), who stated that ε_A cannot exceed 0.05 for swimming, paddling mammals whose swimming speeds are typically low. Fish (1996) measured the ε_A for the muskrat and found it to peak at 0.046 at 0.75m/s, whereas for ducks, humans, and minks, the values are 0.047, 0.052, and 0.014, respectively (Fish, 1992, 1984 and references therein). Unfortunately, we cannot use the velocity-dependent equation of Hind and Gurney (1997) because it is calibrated with the speed range observed in seals and we do not have the full range of observed velocities which would be needed to allow a similar calibration for our model organisms. Blanco (2023) selected a muscle efficiency of 0.1 as a conservative lower value for swimming dinosaurs, in comparison with 0.2 used by Massare (1988) for swimming marine reptiles. However, unlike Massare (1988), Blanco (2023) sought to calculate maximum speeds as opposed to optimal speeds. Nevertheless, the results of Gibbs and Gibson (1972), as well as the equation by Hind and Gurney (1997), suggest that even 0.1 is probably an overestimate, regardless of whether this value is used for the optimal or maximum speed calculation. This is because 0.1 will not correspond to the peak ε_A, but to a lower value since the peak ε_A would coincide with an intermediate stress and therefore certainly not to maximum speed (maximum stress) or optimum speed (probably lower than what is traditionally considered 'intermediate stress' because the optimal speed is the most economical speed). Thus, considering the results for peak ε_A in ducks, humans, minks, and muskrats, 0.1 still seems to be a very high value even in the face of a probable increase in efficiency due to the large size of dinosaurs (Blanco, 2023 and references therein). Further corroboration of this point comes from the aerobic efficiencies of turtles. Turtles in general have higher aerobic efficiencies than other tetrapods of similar body size (values of 0.35 are typical, compared to 0.2 in mice and dogfish), which is probably related to specialized anatomy and muscle physiology (mainly composed of slow-twitch type I muscle fibres), among other factors (Ewart et al., 2022 and references therein). Sea turtles use forelimb flapping for thrust-generation in water, and their aerobic efficiency drops compared to their terrestrial relatives moving on land; yet, it is still higher than in

terrestrial mammal paddlers. For example the green sea turtle's ε_A was estimated to peak around 0.09 (Fish, 1984 citing Prange, 1976) (but for a range of low speeds including optimal speed; conceivably at higher speeds aerobic efficiency could be higher). It seems highly unlikely that a terrestrial dinosaur had an aerobic efficiency as high as that of a sea turtle in water. Therefore, we used the maximum possible value (0.05) stated by Fish (1996) for the paddling animals, except for the tortoise, for which we used a higher value of 0.09 to account for its elevated aerobic efficiency compared to the other paddlers (main text: Table 1).

For the crocodile, which uses undulatory locomotion in its habitual movement in water, the value was kept as 0.2 (main text: Table 1) following the example of Villamil et al. (2016) and Massare (1988) for other marine reptiles.

S3.2. Propulsive efficiency

The propulsive efficiency (ε_p), also known as hydrodynamic or Froude efficiency in some contexts (though the terms are not strictly synonymous; see Zamparo et al. (2020, 2011)), of the swimming mode is the ratio of thrust power (i.e. the useful power output= $\varepsilon_A * \varepsilon_p * M_L$) to total power (total mechanical work produced=$\varepsilon_A * M_L$) of an organism (Fish, 2020, 1996) (main text: Figure 1).

The mean for ε_p values proposed for undulating swimmers is 0.51 (Villamil et al., 2016 and references therein) and the range is $0.43<\varepsilon_p<0.54$ for a typical undulating fish, such as the American eel (Fish et al., 2021). Fish et al. (2021b) speculated that an alligator could possibly exceed the propulsive efficiency of an eel, but they pointed out that drag associated with the possession of limbs must be taken into account for a proper comparison. Given the absence of more appropriate data, a value of $\varepsilon_p=0.5$ is taken here for the saltwater crocodile (main text: Table 1) by rounding the mean (0.485) of the range $0.43<\varepsilon_p<0.54$ (Fish et al., 2021), which is also broadly consistent with the 0.51 average obtained by Villamil et al. (2016 and references therein).

Webb and Blake (1985) stated that propulsive efficiency for paddlers is between 0.2 and 0.3. For semi-aquatic paddling mammals, such as the muskrat and rice rat, which are among the few paddlers where experimental data are available, the maximum ε_p for paddling is 0.33 and 0.25 respectively (Fish, 1992, 1984), with values around 0.33 generally considered to be the maximum for drag-based oscillatory systems (Fish, 2000, 1996, 1992). Blanco (2023) used the conservative value of 0.16 for dinosaurs, basing his choice on studies by Blake (1980, 1979) on fish whole fin-beat cycles. The latter studies accounted for the added mass moved by the fins and also included the reduction of the fin's power stroke thrust alone (with ε_p = 0.18 for that case), by adjusting for the drag caused during the recovery stroke (a mere 11% reduction). However, Blanco (2023) effectively used a λ parameter equal to 1, unlike us who used a value of 2 (See below); this acts as the enhancer of active drag and thereby the reducer of propulsive efficiency by a factor of 2 during the paddling cycle. Hence, the maximum propulsive efficiencies of paddling mammals mentioned above are considered to provide a more appropriate range here, although ultimately there is no right choice given our lack of empirical data. As such, the preferred value for the propulsive efficiency (ε_p) is here taken to be the rounded value of 0.3 for the two dinosaurs, the ostrich, the elephant and the polar bear, with sensitivity tests exploring the impact of lowering this value to 0.25 and 0.16 and

raising it to 0.33. Thus, for the paddling animals, the ε_p is varied within the plausible range of 0.16–0.33 across the sensitivity tests (main text: Table 1).

One issue to consider here is that the propulsive efficiency (ε_p) of quadrupedal paddling can be lower than bipedal paddling because of physical interference between forelimbs and hindlimbs, which increases interference drag (Fish, 1993; Williams, 1983). However, due to a lack of available data with regards to our selected animals, we have opted not to make any subjective adjustment to differentiate the value of ε_A of our bipedal paddlers (the ostrich and potentially *Lambeosaurus*) from the quadrupedal ones. Another issue arises when a tetrapod employs facultative bipedalism. For example, hadrosaurs employed bipedal and quadrupedal terrestrial gaits (e.g. see Sellers et al., 2009), so bipedal and/or quadrupedal paddling might both have been options during aquatic locomotion. If hadrosaurs swam using their hindlimbs alone, they could potentially have had a higher ε_p compared to a quadrupedal animal. This would mean a higher swimming speed and, if this just involved a mere switch in gait form, it could mean that (for example) bipedal swimming was used for quickly fleeing from predators in water. In any case, for long distance swimming, it seems unlikely that hadrosaurs did not use their forelimbs, at least for some form of awkward stabilization, especially when coping with waves. For this reason, we did not attempt to differentiate between these different scenarios for the ε_p of the hadrosaur model.

S3.3. The λ parameter

The exact definition of λ is currently not well established (Villamil et al., 2016). While some authors (e.g. Motani, 2002) have interpreted it as a general correction factor required to account for cumulative parameter-errors resulting in differences between the predicted results of their optimal speed equation compared to the observed values, others (Hind and Gurney, 1997) have interpreted it as the ratio of active to passive drag. Regardless of this lack of agreement, λ depends on the specific swimming mode and is unknown for all of our modeled organisms. Consequently, accurate predictions of trans-oceanic swimming distances and durations are severely hampered without some sort of advanced dynamic CFD simulation or measurement experiment involving the real animal. Pending such studies, we must therefore rely on values of λ derived from the literature. Studies on human swimmers have shown that λ (interpreted as the active to passive drag ratio) is between 1.5 and 2.5 (Zamparo et al., 2020 and references therein), so we used the mean value of 2 for limb-based ('paddling') swimming (main text: Table 1). For whales, Gough et al., (2019 and references therein) refer to values between ~2–3 but as a drag correction factor due to the heaving body and caudal tail. For undulatory swimmers, such as crocodiles, we are not aware of any values of λ. However, given that in the *Spinosaurus*, the drag during undulation was estimated to be 3 and 5 times the stationary drag (Sereno et al., 2022 and references therein), we have assumed that similar values would occur in crocodilians. Therefore, for our crocodile model, we selected the mid-range value of 4 (main text: Table 1) which is probably a conservative (higher than reality) value given that U_{opt} (and thus the drag) would be lower overall compared to the maximum values estimated for *Spinosaurus*.

S3.4 Sensitivity Tests

The sensitivity tests for the hydrodynamic parameters of the U_{opt} equation are shown in table S3.1.

**ε_A*ε_p/λ**		**λ**		
**ε_A*ε_p**		1.5	2	2.5
0.0297	=0.9*0.33	0.0198	0.0149	0.0119
0.027	=0.9*0.3	0.0180	0.0135	0.0108
0.0225	=0.9*0.25	0.0150	0.0113	0.0090
0.0144	=0.9*0.16	0.0096	**0.0072**	0.0058
0.0165	=0.05*0.33	0.0110	**0.0083**	0.0066
0.015	=0.05*0.3	0.0100	**0.0075**	0.0060
0.0125	=0.05*0.25	0.0083	**0.0063**	0.0050
0.008	=0.05*0.16	0.0053	0.0040	0.0032

Table S3.1. Range of values for possible combinations of literature sampled values, of the three parameters ε_A ε_p and λ for our non-avian dinosaurs and some of the other animals (see main text Table 3). Combinations chosen for sensitivity tests are shown in bold.

References

Blake, R.W., 1980. The Mechanics of Labriform Locomotion: II. An analysis of the recovery stroke and the overall fin-beat cycle propulsive efficiency in the Angelfish. Journal of Experimental Biology 85, 337–342.

Blake, R.W., 1979. The Mechanics of Labriform Locomotion: I. Labriform Locomotion in the Angelfish (*Pterophyllum Eimekei*): an Analysis of the Power Stroke. Journal of Experimental Biology 82, 255–271. https://doi.org/10.1242/jeb.82.1.255

Blanco, R.E., 2023. *Tyrannosaurus rex* runs again: a theoretical analysis of the hypothesis that full-grown large theropods had a locomotory advantage to hunt in a shallow-water environment. Zoological Journal of the Linnean Society 198, 202–219. https://doi.org/10.1093/zoolinnean/zlac104

Ewart, H.E., Tickle, P.G., Sellers, W.I., Lambertz, M., Crossley, D.A., Codd, J.R., 2022. The metabolic cost of turning right side up in the Mediterranean spur-thighed tortoise (*Testudo graeca*). Sci Rep 12, 431. https://doi.org/10.1038/s41598-021-04273-w

Fish, F.E., 2020. Aquatic Animals Operating at High Reynolds Numbers: Biomimetic Opportunities for AUV Applications, in: Soboyejo, W., Daniel, L. (Eds.), Bioinspired Structures and Design. Cambridge University Press, pp. 235–270. https://doi.org/10.1017/9781139058995.011

Fish, F.E., 2000. Biomechanics and Energetics in Aquatic and Semiaquatic Mammals: Platypus to Whale. Physiological and Biochemical Zoology 73, 683–698. https://doi.org/10.1086/318108

Fish, F.E., 1996. Transitions from Drag-based to Lift-based Propulsion in Mammalian Swimming. Am Zool 36, 628–641. https://doi.org/10.1093/icb/36.6.628

Fish, F.E., 1993. Comparison of Swimming Kinematics between Terrestrial and Semiaquatic Opossums. Journal of Mammalogy 74, 275–284. https://doi.org/10.2307/1382382

Fish, F.E., 1992. Aquatic Locomotion, in: Tomasi, T.E., Horton, T.H. (Eds.), Mammalian Energetics: Interdisciplinary Views of Metabolism and Reproduction. Comstock Pub. Associates, Ithaca, N.Y.

Fish, F.E., 1984. Mechanics, Power Output and Efficiency of the Swimming Muskrat (*Ondatra Zibethicus*). Journal of Experimental Biology 110, 183–201. https://doi.org/10.1242/jeb.110.1.183

Fish, F.E., Rybczynski, N., Lauder, G.V., Duff, C.M., 2021. The Role of the Tail or Lack Thereof in the Evolution of Tetrapod Aquatic Propulsion. Integrative and Comparative Biology 61, 398–413. https://doi.org/10.1093/icb/icab021

Gibbs, C.L., Gibson, W.R., 1972. Energy production of rat soleus muscle. American Journal of Physiology-Legacy Content. https://doi.org/10.1152/ajplegacy.1972.223.4.864

Gough, W.T., Segre, P.S., Bierlich, K.C., Cade, D.E., Potvin, J., Fish, F.E., Dale, J., di Clemente, J., Friedlaender, A.S., Johnston, D.W., Kahane-Rapport, S.R., Kennedy, J., Long, J.H., Oudejans, M., Penry, G., Savoca, M.S., Simon, M., Videsen, S.K.A., Visser, F., Wiley, D.N., Goldbogen, J.A., 2019. Scaling of swimming performance in baleen whales. Journal of Experimental Biology 222, jeb204172. https://doi.org/10.1242/jeb.204172

Hind, A.T., Gurney, W.S., 1997. The metabolic cost of swimming in marine homeotherms. J Exp Biol 200, 531–542. https://doi.org/10.1242/jeb.200.3.531

Lovelace, D.M., Hartman, S.A., Mathewson, P.D., Linzmeier, B.J., Porter, W.P., 2020. Modeling Dragons: Using linked mechanistic physiological and microclimate models to explore environmental, physiological, and morphological constraints on the early evolution of dinosaurs. PLoS ONE 15, e0223872. https://doi.org/10.1371/journal.pone.0223872

Massare, J.A., 1988. Swimming capabilities of Mesozoic marine reptiles: implications for method of predation. Paleobiology 14, 187–205. https://doi.org/10.1017/S009483730001191X

Motani, R., 2002. Swimming speed estimation of extinct marine reptiles: energetic approach revisited. Paleobiology 28, 251–262. https://doi.org/10.1666/0094-8373(2002)028<0251:SSEOEM>2.0.CO;2

Prange, H.D., 1976. Energetics of swimming of a sea turtle. Journal of Experimental Biology 64, 1–12. https://doi.org/10.1242/jeb.64.1.1

Sellers, W.I., Manning, P.L., Lyson, T., Stevens, K., Margetts, L., 2009. Virtual Palaeontology: Gait Reconstruction of Extinct Vertebrates Using High Performance Computing. Palaeontologia Electronica 12, 1–26.

Sereno, P.C., Myhrvold, N., Henderson, D.M., Fish, F.E., Vidal, D., Baumgart, S.L., Keillor, T.M., Formoso, K.K., Conroy, L.L., 2022. *Spinosaurus* is not an aquatic dinosaur. eLife 11, e80092. https://doi.org/10.7554/eLife.80092

Smith, N.P., Barclay, C.J., Loiselle, D.S., 2005. The efficiency of muscle contraction. Progress in Biophysics and Molecular Biology 88, 1–58. https://doi.org/10.1016/j.pbiomolbio.2003.11.014

Villamil, J., Demarco, P.N., Meneghel, M., Blanco, R.E., Jones, W., Rinderknecht, A., Laurin, M., Piñeiro, G., 2016. Optimal swimming speed estimates in the Early Permian mesosaurid *Mesosaurus tenuidens* (Gervais 1865) from Uruguay. Historical Biology 28, 963–971. https://doi.org/10.1080/08912963.2015.1075018

Webb, P.W., Blake, R.W., 1985. Chapter 7. Swimming, in: Hildebrand, M., Bramble, D.M., Liem, K.F., Wake, D.B. (Eds.), Functional Vertebrate Morphology. Harvard University Press, pp. 110–128. https://doi.org/10.4159/harvard.9780674184404.c7

Williams, T.M., 1983. Locomotion in the North American mink, a semi-aquatic mammal. I. Swimming energetics and body drag. J Exp Biol 103, 155–168. https://doi.org/10.1242/jeb.103.1.155

Zamparo, P., Capelli, C., Pendergast, D., 2011. Energetics of swimming: a historical perspective. Eur J Appl Physiol 111, 367–378. https://doi.org/10.1007/s00421-010-1433-7

Zamparo, P., Cortesi, M., Gatta, G., 2020. The energy cost of swimming and its determinants. Eur J Appl Physiol 120, 41–66. https://doi.org/10.1007/s00421-019-04270-y
