# Supplementary material for: ENHYDROSS: A New Mechanistic Model Supports the Trans‐Oceanic Dispersal Capability of Terrestrial Vertebrates
Source: Ecol Evol. 2026 Mar 30;16(4):e73280. doi: 10.1002/ece3.73280 (PMC13107292; doi:10.1002/ece3.73280)
Supplement: Supplementary file 4 — Data S4: ece373280‐sup‐0004‐SupplefileS4.pdf. [file ECE3-16-e73280-s002.pdf]

S4. Selection criteria for modelled organisms

In order to validate our ENHYDROSS model, we tested it against data from documented cases of swimming in open water in extant animals. The choice of model organisms was based on several criteria. First, given that our model is about active swimming and energetic reserves, we have concentrated our efforts on finding exemplar cases from moderate to large (>1 m length) vertebrates because swimming capabilities and energetic reserves become more biogeographically relevant with increasing body size (or at least the study by Meijaard (2001) would suggest so). Conversely, smaller vertebrates would be expected to disperse more easily via other means (e.g. rafting), depending on the species and a multitude of other factors (see e.g. Mazza et al., [2019, 2013]). Moreover, while our swimming model can be applied to smaller animals, its biogeographic relevance is limited in such cases, as smaller animals do not typically swim long distances at regional or continental scales. Second, we want to include both endotherms and ectotherms in order to increase the range of applicability of our model. Third, we seek to make ENHYDROSS capable of modelling not just mammals, but also birds and reptiles. The fourth, but most important criterion, was availability of relevant empirical data such as observed swimming speed, distance, and duration, as well as anatomical (surface area) and physiological data (fat % of body mass). The best fits to these criteria are: an Asian elephant (*Elephas maximus*) and a polar bear (*Ursus maritimus*), representing large mammalian endotherms; an ostrich (*Struthio camelus*) as an example of a large avian endotherm; and a saltwater crocodile (*Crocodilus porosus*) and an Aldabra giant tortoise (*Aldabrachelys gigantea*) as moderate to large reptilian ectotherms. None of our model animals have information for all three parameters of swimming speed, distance, and duration, and in one case (the ostrich), none of these are known empirically. Inclusion of the latter animal, however, was deemed worthwhile as an exploratory test with regards to a cursorial avian swimming via paddling, rather than necessarily a validating one.

Beyond the validation of our model, our study has the additional goal of applying it to test biogeographic hypotheses concerning Cretaceous dinosaurs. Hence, we have also selected non-avian dinosaur models using another set of criteria. First we chose species that we consider potentially capable of swimming long distances based on their floating posture (Henderson, 2014, 2004) and (not very poor) hydrostatic lateral stability based on their metacentric height (Henderson, 2018). No ceratopsian or ankylosaurian ornithischian was selected because it is highly unlikely that such animals would have been able to swim long distances while continuously struggling to raise their head above the water surface to breathe (see Henderson, 2014; Mallon et al., 2018). This, we assume, would have made them poor long-distance swimmers and thus we did not select them for testing. The second criterion focused on dinosaur groups that have been hypothesized as taking part in trans-oceanic dispersals during the Cretaceous based on probabilistic biogeographic inference. One such group is the hadrosaurs, such as the lineage that gave rise to the Moroccan lambeosaurines *Ajnabia odysseus* (Longrich et al., 2021) and *Minqaria bata* (Longrich et al., 2024). Such derived hadrosaurids are hypothesized to have arrived in Africa by crossing the Tethys from Europe sometime during the Campanian–Maastrichtian (Longrich et al., 2024, 2021). The titanosaurian sauropods, specifically a clade of Afro-Eurasian taxa, including the middle Campanian African species *Mansourasaurus shahinae* (Sallam et al., 2018) and *Igai semkhu* (Gorscak et al., 2023) and the early Maastrichtian Ibero-Armorican taxon *Abditosaurus kuehnei* (Vila et al., 2022), display phylogenetic relationships that suggest dispersals between Europe and Africa at approximately the same time (or slightly earlier) as the hadrosaurs (see

Díez Díaz et al., 2025; Upchurch, In press). The third and final criterion was that there was sufficient information through skeletal remains for an accurate 3D silhouette reconstruction. For this we had to choose closely related species with excellent preservation and skeletal completeness. The hadrosaur model selected represents *Lambeosaurus lambei*. Specifically, the model was constructed based on the body of the nearly identical in overall morphology *Corythosaurus casuarius* (Paul, 1987, page 42, Fig. 25) whereas the crest was based on the *L. lambei* specimen (TMP82.38.01) from the Royal Tyrell Museum of Paleontology. Our titanosaur model is based on *Rapetosaurus krausei* (Curry Rogers, 2009; Curry Rogers and Forster, 2001). Even by satisfying these three criteria, problems remained, in that we lacked several physiological and other kinds of biomechanical data, crucial for utilizing our model. To fill the gaps in our knowledge of dinosaurs, we have made inferences based on living taxa. These include: metabolic rates and how these scale with body mass; how much fat mass is realistically expected for a dinosaur; and the values for the various parameters of the optimal swimming speed (ε_p, ε_A and λ).

References

Curry Rogers, K., 2009. The postcranial osteology of *Rapetosaurus krausei* (Sauropoda: Titanosauria) from the Late Cretaceous of Madagascar. Journal of Vertebrate Paleontology 29, 1046–1086. https://doi.org/10.1671/039.029.0432

Curry Rogers, K., Forster, C.A., 2001. The last of the dinosaur titans: a new sauropod from Madagascar. Nature 412, 530–534. https://doi.org/10.1038/35087566

Díez Díaz, V., Mannion, P.D., Csiki-Sava, Z., Upchurch, P., 2025. Revision of Romanian sauropod dinosaurs reveals high titanosaur diversity and body-size disparity on the latest Cretaceous Haţeg Island, with implications for titanosaurian biogeography. Journal of Systematic Palaeontology 23, 2441516. https://doi.org/10.1080/14772019.2024.2441516

Gorscak, E., Lamanna, M.C., Schwarz, D., Díaz, V.D., Salem, B.S., Sallam, H.M., Wiechmann, M.F., 2023. A new titanosaurian (Dinosauria: Sauropoda) from the Upper Cretaceous (Campanian) Quseir Formation of the Kharga Oasis, Egypt. Journal of Vertebrate Paleontology e2199810. https://doi.org/10.1080/02724634.2023.2199810

Henderson, D.M., 2018. A buoyancy, balance and stability challenge to the hypothesis of a semi-aquatic *Spinosaurus* Stromer, 1915 (Dinosauria: Theropoda). PeerJ 6, e5409. https://doi.org/10.7717/peerj.5409

Henderson, D.M., 2014. Duck Soup: The Floating Fates of Hadrosaurs and Ceratopsians at Dinosaur Provincial Park, in: Hadrosaurs, Life of the Past. Indiana University Press, Bloomington, pp. 459–466.

Henderson, D.M., 2004. Tipsy punters: sauropod dinosaur pneumaticity, buoyancy and aquatic habits. Biol. Lett. 271, S180–S183. https://doi.org/10.1098/rsbl.2003.0136

Henderson, D.M., 2003. Effects of stomach stones on the buoyancy and equilibrium of a floating crocodilian: a computational analysis. Can. J. Zool. 81, 1346–1357. https://doi.org/10.1139/z03-122

Longrich, N.R., Pereda-Suberbiola, X., Bardet, N., Jalil, N.-E., 2024. A new small duckbilled dinosaur (Hadrosauridae: Lambeosaurinae) from Morocco and dinosaur diversity in the late Maastrichtian of North Africa. Sci Rep 14, 3665. https://doi.org/10.1038/s41598-024-53447-9

Longrich, N.R., Pereda-Suberbiola, X., Pyron, R.A., Jalil, N.-E., 2021. The first duckbill dinosaur (Hadrosauridae: Lambeosaurinae) from Africa and the role of oceanic dispersal in dinosaur biogeography. Cretaceous Research 120, 104678. https://doi.org/10.1016/j.cretres.2020.104678

Mallon, J.C., Henderson, D.M., McDonough, C.M., Loughry, W.J., 2018. A "bloat-and-float" taphonomic model best explains the upside-down preservation of ankylosaurs. Palaeogeography, Palaeoclimatology, Palaeoecology 497, 117–127. https://doi.org/10.1016/j.palaeo.2018.02.010

Mazza, P.P.A., Buccianti, A., Savorelli, A., 2019. Grasping at straws: a re-evaluation of sweepstakes colonisation of islands by mammals: Natural rafting of land mammals. Biol Rev 94, 1364–1380. https://doi.org/10.1111/brv.12506

Mazza, P.P.A., Lovari, S., Masini, F., Masseti, M., Rustioni, M., 2013. A Multidisciplinary Approach to the Analysis of Multifactorial Land Mammal Colonization of Islands. bisi 63, 939–951. https://doi.org/10.1525/bio.2013.63.12.7

Meijaard, E., 2001. Successful sea-crossings by land mammals; a matter of luck, and a big body. A preliminary and simplified model. Geol. Res. Dev. Centre, Spec. Publ 87–92.

Paul, G.S., 1987. The science and art of restor-ing the life appearance of dinosaurs and their relatives: a rigorous how-to guide, in: Czerkas, S.M., Olson, E.C. (Eds.), Dinosaurs Past and Present. Natural History Museum of Los Angeles County/University of Washington Press, Seattle and Washington, pp. 5–49.

Sallam, H.M., Gorscak, E., O'Connor, P.M., El-Dawoudi, I.A., El-Sayed, S., Saber, S., Kora, M.A., Sertich, J.J.W., Seiffert, E.R., Lamanna, M.C., 2018. New Egyptian sauropod reveals Late Cretaceous dinosaur dispersal between Europe and Africa. Nat Ecol Evol 2, 445–451. https://doi.org/10.1038/s41559-017-0455-5

Sereno, P.C., Myhrvold, N., Henderson, D.M., Fish, F.E., Vidal, D., Baumgart, S.L., Keillor, T.M., Formoso, K.K., Conroy, L.L., 2022. *Spinosaurus* is not an aquatic dinosaur. eLife 11, e80092. https://doi.org/10.7554/eLife.80092

Upchurch, P., In press. The biogeographic history of Mesozoic dinosaurs, in: Weishampel, D.B., Barrett, P.M., Makovicky, P.J., Carrano, M.T. (Eds.), The Dinosauria III. Cambridge University Press, Cambridge.

Vila, B., Sellés, A., Moreno-Azanza, M., Razzolini, N.L., Gil-Delgado, A., Canudo, J.I., Galobart, À., 2022. A titanosaurian sauropod with Gondwanan affinities in the latest Cretaceous of Europe. Nat Ecol Evol 6, 288–296. https://doi.org/10.1038/s41559-021-01651-5
