# Supplementary material for: ENHYDROSS: A New Mechanistic Model Supports the Trans‐Oceanic Dispersal Capability of Terrestrial Vertebrates
Source: Ecol Evol. 2026 Mar 30;16(4):e73280. doi: 10.1002/ece3.73280 (PMC13107292; doi:10.1002/ece3.73280)
Supplement: Supplementary file 5 — Data S5: ece373280‐sup‐0005‐SupplefileS5.pdf. [file ECE3-16-e73280-s001.pdf]

S5. Additional tests

S5.1. Testing various components in Meijaard’s (2001) model

In order to compare the performance of the ENHYDROSS model, we also ran a separate set of analyses using Meijaard's (2001) methodology for our animals. This set of analyses comprises an exact replicate of Meijaard's (2001) method (See Supplementary File S1) and three additional variants of it. In the first variant, instead of using the snout-vent length as waterline length, we used the more precise values obtained from the buoyancy analyses by DMH or by our calculations in the case of the tortoise. The exception to this was the polar bear model, for which the waterline length was always set equal to snout-vent length by assumption (see Supplementary File S6). For the second variant, instead of employing the empirical formula for calculating fat mass for mammals used by Meijaard (2001), we used the more accurate fat masses for each animal that we compiled from the literature and incorporated into the ENHYDROSS model. In the third variant, instead of using Meijaard's (2001) U_{opt} equation (which was based on just two data points for the 16.5% fraction of hull speed), we utilised the statistically more powerful equation presented by Hedenström, (2003, equation 6) which gives the migratory speed of swimming animals in m/s:

$$U_{migr} = 0.11\, m^{0.17} \qquad \text{(S5.1)}$$

Although migratory speed does not necessarily coincide with U_{opt}, it is a good substitute: migratory animals need to conserve energy while travelling, so assuming that they move at or close to U_{opt} is reasonable. In this third variant, we also incorporated the same fat masses used in the second variant.

S5.2. Applying a BMR enhancement factor in the ENHYDROSS model

In order to compare Meijaard's (2001) model with our ENHYDROSS model, we ran an analysis for the latter using an enhanced BMR for each endothermic animal, so as to approximate the higher energetic cost that would be expected if additional thermogenesis (M_T) had occurred. Because there is currently no way to calculate M_T in the ENHYDROSS model, we used a simple multiplication of BMR by a factor of 2.3 for our endothermic extant animals (ostrich and elephant), as well as BMRs derived from all the mammalian and avian allometric equations used for the two non-avian dinosaurs. This value comes from Williams (2022), who calculated it to be the multiplicative difference between the expected and measured BMR values of marine mammals based on McNab's (2008) BMR scaling relationships for terrestrial mammals. The assumption here is that, much as the BMR of marine mammals is on average approximately 2.3 times higher due to the substantially greater heat loss imposed by aquatic environments, swimming terrestrial endothermic animals will elevate their BMR in much the same way to produce additional heat (M_T) so they can maintain their body temperature. Strictly speaking, such a manipulation is forbidden for two reasons: a) M_T depends on the swimming velocity and so multiplying by 2.3 would violate the optimization calculation; and b) the optimization does not account for temperature variance because our initial assumption for the ENHYDROSS model was that of null thermogenesis (M_T=0), which for endotherms presupposes either thermoneutral conditions or 100% thermal substitution. Additionally, for the same reasons we found Meijaard's (2001) utilization of a single number (a multiple of COT) for all terrestrial mammals (see Supplementary File S1) to be problematic, we also

consider the 2.3×BMR manipulation problematic as a treatment for all terrestrial vertebrates. However, before a next generation ENHYDROSS-like model is developed in a future study, this analysis serves as an estimation as to how far our model's results are from those obtained by Meijaard's (2001) model, especially in terms of COT. This is a useful comparison because the use of Williams's (1999) COT in Meijaard's (2001) approach does in fact account for a variable (and unknown amount of) thermogenesis depending on the species. Finally, we did not apply this enhancement to the polar bear because it is a marine mammal, and so this enhancement factor is already accounted for in the BMRs used by Williams (2022).

S5.3. Comparisons between ENHYDROSS and Meijaard's (2001) models

The observed differences between our model and that of Meijaard (2001) can be attributed, for the most part, to the very high COT_{min} of the latter obtained from Williams' (1999) equation (see main text: Table 5, Supplementary File S1: Equation S3). Besides the concern we expressed when explaining the problems of using a single multiplier for COT, the maximum possible coefficient of x5.1 used in Williams' (1999) equation is probably an over-estimate in terms of energetic cost. This is especially true under conditions of null-thermogenesis, which constitute the baseline assumption of our model and are most readily satisfied under relatively warm water temperatures. From our model results, only the polar bear's COT_{min} falls close to the results of Meijaard (2001) models (slightly above and below), but this is hardly a surprise since its metabolism is elevated for a mammal of its size and this is taken into account in our approach by the use of the respective metabolic rate equation for polar bears. Even when applying the 2.3 x BMR enhancement in our model, our estimated COT_{min} values approach those obtained from Meijaard's (2001) models only in the non-avian dinosaurs (and then only barely exceed them when the highest endothermic BMRs are assumed). By contrast, under the 2.3 x BMR enhancement, COT_{min} values for the ostrich and elephant are 39.3% and 23.3% lower, respectively, than those produced by Meijaard's (2001) model (main text: Figure 3, Table 5; see also the ENHYDROSS core dataset (Excel file) available on Zenodo: https://doi.org/10.5281/zenodo.18015597).

The feasible dispersal distance estimates obtained when applying the 2.3 x BMR enhancement are substantially smaller than any of the other ENHYDROSS sensitivity test results, highlighting the radical difference between cases of M_T=0 and cases where thermogenesis is present (M_T>0). It comes as no surprise, therefore, that the high COT_{min} estimates of the Meijaard models translate into substantially smaller feasible distances (and durations) compared to those derived from our model. Thus, we strongly suspect that even if the smaller Meijaard dispersal distance estimates appear more realistic (e.g. elephant and ostrich) when compared to empirically observed values in the wild, our model is likely to be far more accurate in terms of biophysics. The severely underestimated dispersal durations and distances produced by the Meijaard family of models for the tortoise and crocodile are a direct consequence of this. The higher mammalian based COT_{min} obtained from William's equation, increases the COT_{min} more than 10 times, compared to the ENHYDROSS model, for these ectothermic animals. This means that effectively, these ectothermic reptiles were modeled as mammal-like in the way they burned energy (fat). Clearly this is erroneous, but it is expected because the Meijaard model (and hence its variants) were developed for mammals alone. This family of models also underestimates the swimming duration for the polar bear, but only when the fat mass value used was calculated by Prothero's (1995) scaling

equation (but see main text section 4.1.2). In the two Meijaard variants where the fat mass was the same as in the ENHYDROSS model, the swimming durations were very similar to the latter's estimates and the empirically observed value of 9 days (main text: Table 4). This merely reflects the fact that polar bears store much greater fat mass than the average mammal because of their demanding lifestyle and extreme environment: this justifies our concern when applying a single allometric equation for calculating a representative fat mass for all mammals. Therefore, we can see that at least in the ectotherms and the polar bear, the original Meijaard model (i.e. William's COT equation) systematically yields underestimates.

S5.4. Comparison of Meijaard's model variants

Amongst the Meijaard's variants, there are differences only with regards to U_{opt}, dispersal distances and durations, because COT_{min} was uniformly calculated in all of them with William's allometric equation. In addition, with regards to U_{opt}, the variant with the adjusted fat mass ("same fat mass") produced exactly the same results as the standard Meijaard model. In the variant with the accurate waterline (L_w), in terms of U_{opt}, all animals except the polar bear (whose L_w=snout-vent length) had noticeably smaller estimates compared to the standard snout-vent Meijaard model (the difference being <0.1 m/s for the majority of cases). Similarly, the variant which used an allometric equation for speed (Hedenstrom's (2003) U_{opt}) instead of the fraction of hull speed, had lower U_{opt} values than the standard snout-vent model, with the exception of the elephant and tortoise. These differences, albeit small, demonstrate the dangers of relating speed to either the concept of waterline alone (whether this is the snout-vent length or the actual L_w), or using an allometric equation with just mass as an input. Animals which do not follow the snout-vent length rule for the waterline, will deviate from the snout-vent length derived U_{opt} and vice versa. Likewise, animals whose body mass is high, will have higher swimming speeds according to the Hedenstrom's (2003) U_{opt} variant, whereas this is an oversimplification because of conflation with size. There are no substantial data to support either of these relationships and it is unlikely that such evidence will ever exists, at least with regards to terrestrial animals because their bauplans are not optimized for swimming. After all, optimal swimming speed is a complex quantity and it is determined by many parameters, as shown by equations (6) and (19) (see main text). By comparison, our ENHYDROSS model is much more versatile by design, to accommodate individual differences in swimming modes and thus produce more accurate speeds.

With regard to estimated dispersal distances, the differences between the Meijaard variants were clearly dependent almost entirely on the value selected for fat mass. Hence, each pair of model variants ('standard model' and 'corrected L_w' on one hand, and 'same fat mass' and 'Hedenstrom's (2003) U_{opt}' on the other) yielded the same distances per pair for each animal considered. The latter pair of variants yielded larger dispersal distances for the elephant, crocodile, bear and, when their fat mass was doubled (10.36%), the non-avian dinosaurs too. The rest of the cases (ostrich, tortoise) had smaller estimated dispersal distances in comparison to those produced by the other pair of models ('standard snout-vent' and 'corrected L_w'). There is also a clear pattern related to the ranking of fat masses. Animals with fat mass larger than the one resulting from Prothero's (1995) equation used by Meijaard (2001) always results in higher dispersal distances, whereas those with lower fat mass result in shorter ones. This highlights once again (see Supplementary File S1 and main text: Section

4.1.1) our concerns regarding using a single allometric equation (let alone a generic one derived from a large sample of mammalian species) to represent all mammals. Estimated feasible dispersal distances are very sensitive to the amount of energy reserves. Therefore, when testing biogeographic hypotheses, one must be careful to: 1) sample an accurate fat quantity tailored to the animals under study; and 2) use a pair of such values to obtain a bracketing range of estimated distances. It is this range of lower and upper estimates of what is feasible, rather than a single distance derived from preferred parameter values and assumption sets, that is potentially most informative when evaluating biogeographic hypotheses.

The situation differs when it comes to feasible swimming duration, as the outcome for the 'same fat mass' and 'Hedenström's (2003) U_{opt}' variants was determined by a combination of U_{opt} and amount of body fat. In addition, the corrected L_w variant yielded longer feasible dispersal durations than those suggested by the standard "snout-vent" model, reflecting exactly the reciprocal relationship seen for U_{opt} between the two variants. The polar bear was an exception, retaining the same value, for the reason explained above. Similar comments to those – (1), (2) – made in the above paragraph also hold for dispersal durations.

References

Hedenström, A., 2003. Scaling migration speed in animals that run, swim and fly. Journal of Zoology 259, 155–160. https://doi.org/10.1017/S0952836902003096

McNab, B.K., 2008. An analysis of the factors that influence the level and scaling of mammalian BMR. Comparative Biochemistry and Physiology Part A: Molecular & Integrative Physiology 151, 5–28. https://doi.org/10.1016/j.cbpa.2008.05.008

Meijaard, E., 2001. Successful sea-crossings by land mammals; a matter of luck, and a big body. A preliminary and simplified model. Geol. Res. Dev. Centre, Spec. Publ 87–92.

Prothero, J., 1995. Bone and fat as a function of body weight in adult mammals. Comparative Biochemistry and Physiology Part A: Physiology 111, 633–639. https://doi.org/10.1016/0300-9629(95)00050-H

Williams, T.M., 2022. Racing Time: Physiological Rates and Metabolic Scaling in Marine Mammals. Integrative And Comparative Biology 62, 1439–1447. https://doi.org/10.1093/icb/icac054

Williams, T.M., 1999. The evolution of cost efficient swimming in marine mammals: limits to energetic optimization. Philos Trans R Soc Lond B Biol Sci 354, 193–201. https://doi.org/10.1098/rstb.1999.0371
