# Supplementary material for: ENHYDROSS: A New Mechanistic Model Supports the Trans‐Oceanic Dispersal Capability of Terrestrial Vertebrates
Source: Ecol Evol. 2026 Mar 30;16(4):e73280. doi: 10.1002/ece3.73280 (PMC13107292; doi:10.1002/ece3.73280)
Supplement: Supplementary file 6 — Data S6: ece373280‐sup‐0006‐SupplefileS6.pdf. [file ECE3-16-e73280-s005.pdf]

S6. Buoyancy tests and associated information

S6.1. Background on buoyancy and hydrostatics

Perhaps the most important difference between an organism living in water and one on land is that in water, the effects of gravity are reduced because buoyant forces can counteract the organism's weight. According to Archimedes' principle, a floating or a submerged object experiences an upward force equal to the weight of the displaced fluid, acting at the *center of buoyancy* (KB) (Giles et al., 1995). The density of an organism dictates whether it has neutral, positive or negative buoyancy when at rest. If an organism's density matches that of the surrounding fluid (i.e. it has neutral buoyancy), there is no vertical (buoyant) force, allowing it to remain suspended in the water column or float at the surface. A denser body is negatively buoyant and sinks, while a less dense body is positively buoyant and rises (Atkinson, 2022). Most animal tissues have a density close to that of water, tending to be slightly positively buoyant (Fish, 1993).

Aquatic animals have developed numerous adaptations to actively (hydrodynamic) or passively (hydrostatic) control their buoyancy. The hydrostatic control variety includes: pachyostotic or osteosclerotic bones, blubber, regulation of air volume in lungs and ingestion of gastroliths. On the other hand, hydrodynamic control is exerted by the body and control surfaces like fins and flippers that produce lift (Gutarra and Rahman, 2022). Terrestrial animals, though less adapted to aquatic life, can increase their buoyancy using similar features when present. For example, they can inflate their lungs, accumulate low-density fat tissue like blubber, possess low bone density from air sacs (as in birds and non-avian dinosaurs) or possess air-trapping feathers and fur. Hydrostatic and hydrodynamic stability is usually aided by overall body shape and limb and tail movements.

There are two main criteria for assessing the hydrostatic stability of a body in water. First, a sufficient but not necessary condition for a body to be stable in water is that the *center of gravity* (KG) must be located below the *center of buoyancy* (KB) of the body (Biran and López-Pulido, 2014: page 41; Giles et al., 1995). If the two points coincide the body is said to be in neutral equilibrium where a small tilt does not produce any righting moment but instead the body would remain in its new position (Giles et al., 1995). If the KG is above the KB then the body is in unstable equilibrium and the slightest motion will misalign the two centers causing a moment force -a torque- (because of the weight force vector of the body in the downwards direction and the buoyancy force vector on the upward direction having a horizontal distance between them) which will cause the body to rotate and tilt until it reaches a stable equilibrium again (Giles et al., 1995; Henderson, 2018).

The second criterion is associated with the position of the metacenter (KM) and the measure of the metacentric height. The metacenter is defined as the point where the centerline of a body intersects the vertical line that passes through the center of buoyancy (KB) when the body is laterally tilted at a small angle (Giles et al., 1995; pp. 58-59). The standard definition of metacentric height is the distance between the metacenter (KM) and the center of gravity (KG) (Barrass and Derrett, 2006; page 45). For a floating body to be stable, its center of gravity (KG) should be below the metacenter (KM) in which case it is said that the body has a *positive metacentric height* (Barrass and Derrett, 2006; page 45-47; Biran and López-Pulido, 2014; pp 40-41; Giles et al., 1995; page 58; Henderson, 2018). Conversely, it is unstable when the KG is

above the KM, in which case it is said that the body has a *negative metacentric height* (Barrass and Derrett, 2006; page 45-47; Biran and López-Pulido, 2014; pp 40-41; Giles et al., 1995; page 58; Henderson, 2018). A larger (positive) metacentric height indicates a higher initial stability of a floating body (Abbas et al., 2021). It follows logically that for cases with a *negative metacentric height*, the latter quantitatively expresses instability: i.e. the greater the distance between the metacenter (KM) and the center of gravity (KG) the higher the instability.

S6.2. Data used for buoyancy analyses

To perform a buoyancy analysis as pioneered by DMH (Henderson, 2018, 2014, 2004, 2003; Henderson and Naish, 2010; Mallon et al., 2018) one needs to first estimate the volume, mass and center of mass for the animal of interest. This is done according to the 3-D mathematical slicing method outlined in Henderson (1999). For that method to work, an animal silhouette must first be obtained, either from photographs or by "fleshed-out" skeletal reconstructions (Henderson, 1999). The Indian elephant model was based on two sources. The basic body form was derived from photographs on page 72 of Muybridge (1887). Underwater photographs of a swimming Indian elephant showing immersed limb and body posture came from Chadwick (1991). The ostrich model was based on two sources. Illustrations of lateral and whole body views were taken from Deeming et al. (1996). DMH personally took measurements of the limbs and axial skeleton of an adult ostrich skeleton held in the Collections of the Royal Tyrrell Museum of Palaeontology. The specimen catalogue number is TMP1997.30.251. For the crocodile the textbook by (Grigg and Kirshner, 2015: pp. 146–147, figs. 4.16-4.18, 4.27; page 155, fig. 4.27) was consulted. For *Rapetosaurus* the silhouette from Curry Rogers and Forster (2001) and for *Lambeosaurus* the one from Paul (1987: page 42, fig. 25) were used to create the 3D models. The *Lambeosaurus* silhouette was constructed based on the body of the nearly identical in overall morphology *Corythosaurus casuarius* (Paul, 1987: page 42, fig. 25) whereas the crest was based on the *L. lambei* specimen (TMP82.38.01) from the Royal Tyrell Museum of Paleontology.

While the volume of each model is estimated using the silhouette selected for each animal (*sensu* Henderson, 1999), the mass is calculated after estimating the respective body densities. However, each animal body is a composite of tissues, lungs, bones and various other features which have different densities that need to be taken into account. Here, the basic axial body density was set to 1000 kg/m^3 for all animals. For the titanosaur, the precaudal pelvic and trunk densities were lowered to 850 kg/m^3 to reflect the presence of air sacs based on the observation that the air sacs in modern birds occupy about 15% of the trunk region; the same trunk density was used for the ostrich as well. The necks of the ostrich and the titanosaur models were further reduced to 300 kg/m^3. The elephant's ears and trunk were also set to 1000 kg/m^3. The limb densities were set to 1050 kg/m^3. This is slightly denser than water to account for the larger fraction of bone relative to soft tissue in the limbs.

Lung volumes were set to somewhere between 8 – 10% of axial body volume. The titanosaur and the ostrich were assigned values towards the higher end of this range, whereas the hadrosaur and the crocodile were assigned values towards the lower end. The elephant lung volume was based on the mammalian lung – body mass scaling relationship presented in (Schmidt-Nielsen, 1984, page 100). The lungs were treated as hollow cavities with a density of air (essentially zero when compared to the other body density values) and represent

localized mass-deficits in the anterior trunk region. The net effect for the presence of lungs is to displace the axial centre of mass posteriorly by a small amount.

The effects of bone pneumaticity and the associated reduction in mass and density are reflected in the regional density variations of the axial body. When calculating the masses and centres of mass of the models, explicit use of the local density variations and the volumes of tissues with those densities was made, to accurately compute the masses of specific body regions. The exact centroid of the lung cavity was also calculated and the effect of that mass deficit was included to further reduce the density in the area immediately surrounding the lungs in the thoracic region. The buoyant nature, and final equilibrium orientation, of the floating titanosaur and ostrich models are a result of accounting for the all-regional mass reductions from the basic 1000 kg/m^3 due to pneumatization and the presence of a lung cavity.

When combined, the result of all the various densities used in axial bodies and limbs give the bulk, whole body densities. The various densities used for each animal are shown in table S6.1.

	Lambeosaurus lambei	*Rapetosaurus krausei*	*Struthio camelus.*	*Elephas maximus*	*Crocodilus porosus*
Axial body density (kg/m^3)	1000	850 (precaudal+trunk); 300 (neck)	850 (trunk); 300 (neck)	1000	1000
Limb density (kg/ m^3)	1050	1050	1050	1050	1050
Lung density (kg/ m^3)	1.3 (Air)	1.3 (Air)	1.3 (Air)	1.3 (Air)	1.3 (Air)
Other body parts density (kg/ m^3)	–	–	–	1000 (ears +proboscis)	–
Total body density (kg/m^3)	912	766	852	881	992

Table S6.1. Various densities used to calculate the mass for each animal model.

S6.3. Waterline length and choice of animal floating pose

For a swimming or floating body, the waterline length is the length of the body that is in contact with the surface of the water. It is typically measured when the object or animal is in its natural floating or swimming position. In cases where the waterline is disconnected, the sum of the constituent lengths gives the waterline length. It is therefore crucial, that for a buoyancy study, a natural pose for the animal of interest is obtained/created. Of course, since animals move, there can be many natural poses (e.g. during a paddling cycle), so in our case we chose a bilaterally symmetrical natural pose where limbs either hang freely or assume a position that seems to be maintained during most of the propulsive phase of swimming.

There is an additional reason for this choice: for paddling animals, a symmetrical configuration of limbs typically (provided that they do not extend horizontally, i.e., parallel to the direction of flow, but instead at an angle to it) results in a larger frontal area exposed to the flow than an asymmetrical configuration. This in turn, results in lowering the U_{opt} by increasing $S_{frontal}$ (which in our case is only the submerged part of the frontal area) and C_d. The increase in C_d stems from our use of an adjusted characteristic width, specifically, an equivalent diameter Z_{eq}, rather than the maximal trunk diameter Z, which incorporates the additional drag produced by the larger submerged frontal area that includes the limbs (see main text: Section 2.1.5).

One can argue that an asymmetrical position of the limbs relative to each other would be more accurate in depicting a typical paddling phase, but at least for the quadrupedal animals this is not necessarily the case because of how different swimming gaits position the front limbs in relation to the hindlimbs. Since we do not know the exact swimming gaits and kinematics for the modelled animals and to keep a consistent methodology (as much as possible given the many uncertainties involved) we chose the bilaterally symmetric position of the limbs (see Figure S6.1). This also produces conservative results of U_{opt}, COT_{min} and maximum swimming distance, which counterbalance to some degree the effect of probable underestimates of C_d, given that the equations used for the latter are for a prolate spheroid (see main text: Section 2.1.5).

S6.4. Data obtained from buoyancy analyses

The results of the buoyancy analyses by DMH are shown in figure S6.1. The waterline length of each modelled animal was measured from the resulting buoyancy figures as shown in figure S6.1. Data on the wetted surface area were provided by DMH. For the frontal submerged area of each animal, the STL files of the 3D models were passed to Rhinoceros 3D version 7.0 software (McNeel, 2020) where the command *MeshOutline* was used to create a closed curve outline around the mesh. Using the height of the waterline relative to the body, as obtained from the buoyancy analyses (Figure S6.1), we delineated the exposed area, selected and removed it, and then calculated the remaining surface area using the command *Area* (Figure S6.2).

As detailed in Section S6.3, frontal area measurements were performed using each animal's designated swimming pose. The crocodile is the only case where the swimming and passive floating poses differ (Figures S6.1 and S6.2). This difference has no effect on the buoyancy analyses but does alter the estimated frontal surface area, as the limbs are held parallel to the body during swimming to reduce drag.

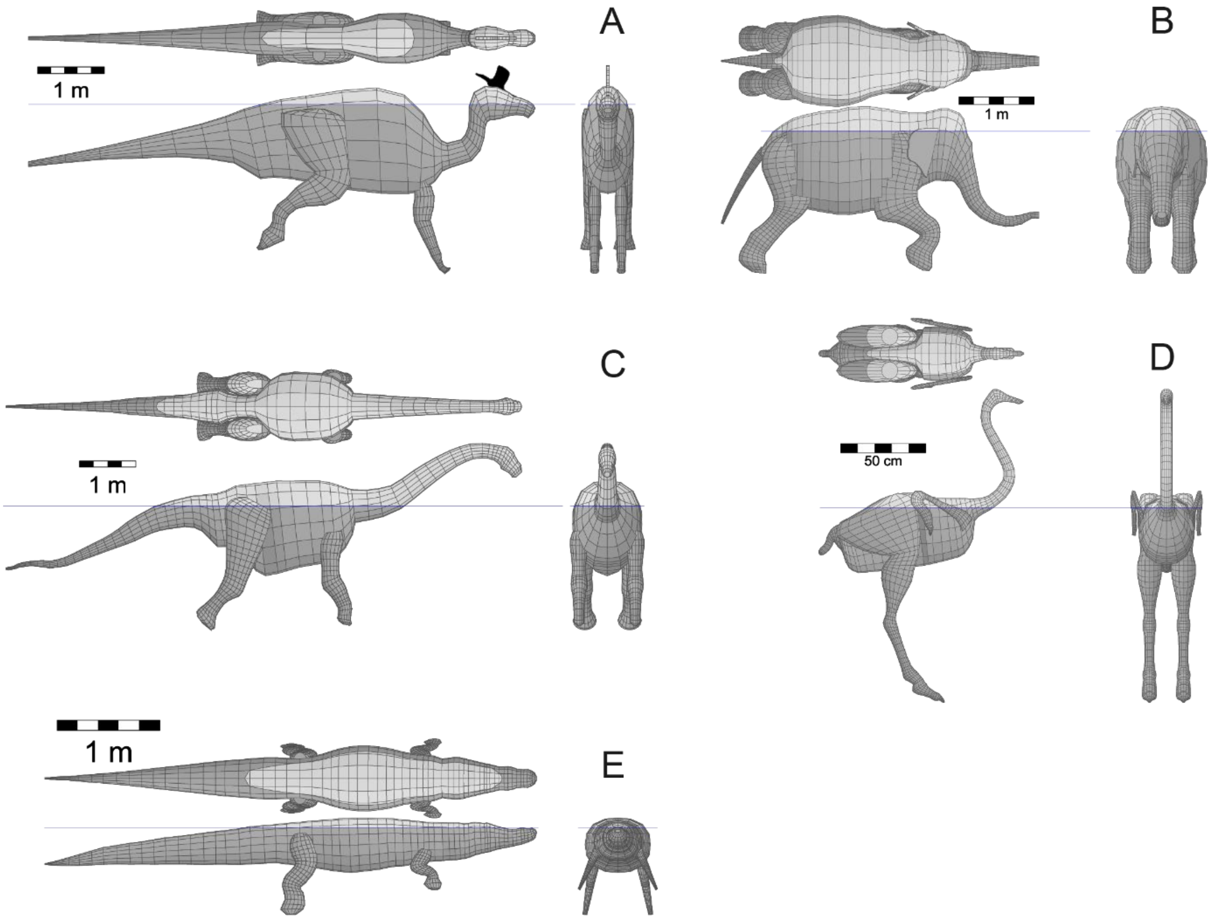

Figure S6.1. Results of buoyancy analyses by DMH. A: *Lambeosaurus lambei*; B: *Elephas maximus*; C: *Rapetosaurus krausei*; D: *Struthio camelus*; E: *Crocodilus porosus*. Blue lines show the water surface level.

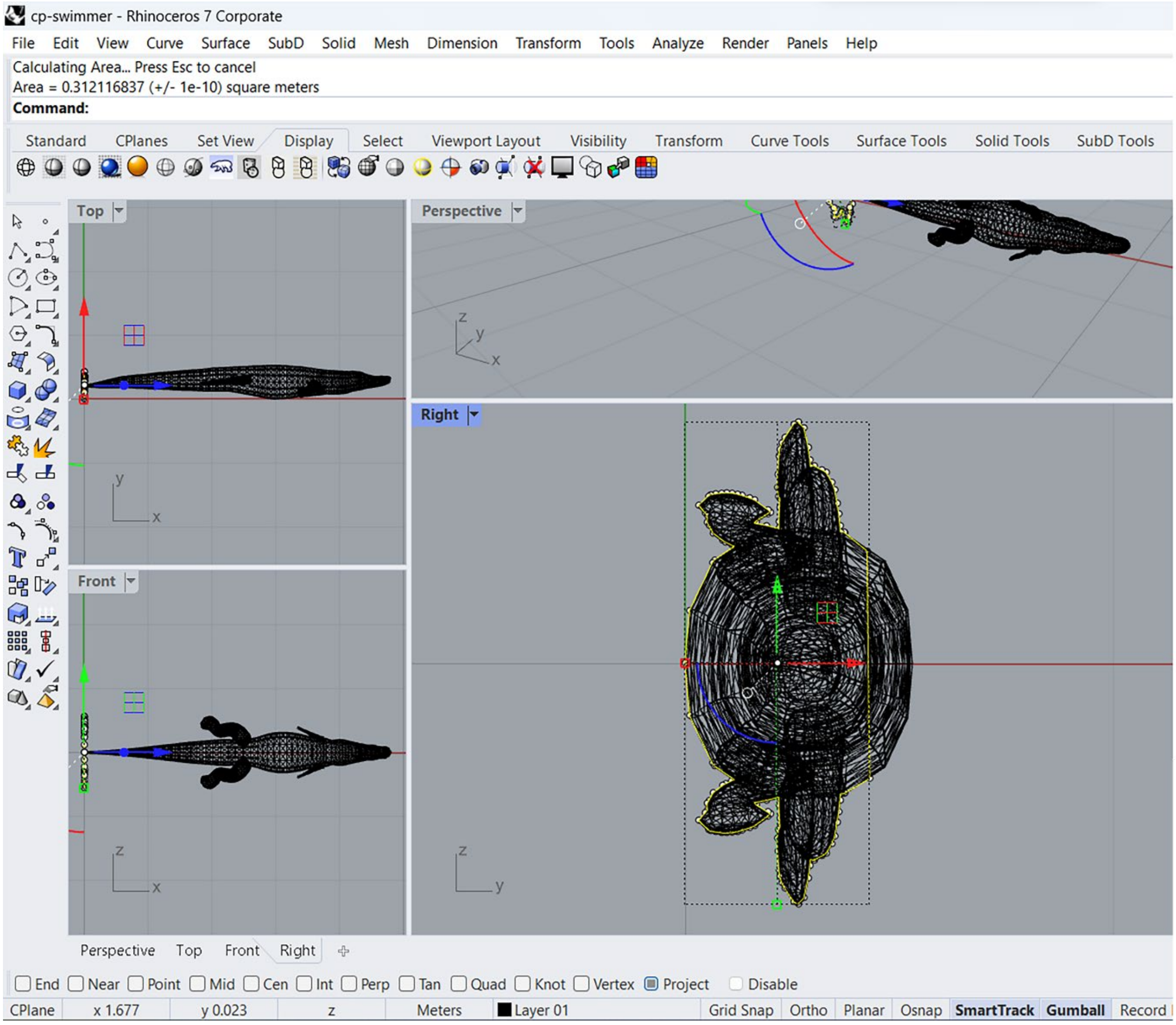

Figure S6.2. Measurement of frontal (cross sectional) area in *Rhinoceros 3D*. Forming a closed curve outline (in yellow color) around the submerged frontal area of the 3D mesh model (bottom right window), as depicted in the results of the buoyancy analyses (Figure S6.1).

S6.5. Geometric data for the polar bear and tortoise

For the polar bear and tortoise, no buoyancy analysis was performed. Instead, parameter values, such as wetted and frontal surface areas and waterline length, were estimated using simple geometric shapes and available data and formulae from the literature, which were deemed sufficient for reasonable precision.

The dimensions of the polar bear were chosen to be the same as those in Mathewson and Porter (2013) for an adult female (2 m length and 0.51 m width and 0.58 m axial body height). The waterline was assumed to be the whole length of the animal, following Meijaard's (2001) snout-vent=waterline length assumption. In this context, this is indeed a reasonable assumption because this is supported by evidence from online videos and in photographs (see also Pagano et al., 2019: fig. 2). During surface swimming, only part of the bear's head remains emerged, and its back is exposed very little (Figure S6.3A). In any case, the waterline itself is mostly relevant to the Meijaard method and its variants (Supplementary Files S1 and S5). In the ENHYDROSS model, the waterline is only useful for marking the boundaries between the

exposed and submerged body areas i.e. for calculating the wetted and frontal surface areas in order to calculate the corresponding drag coefficients. Thus, the snout-vent length for practical matters was taken to be isomorphic to the length of the polar bear in order to simplify the calculation of the wetted and submerged frontal surface areas. The entire body surface area was calculated using equation (3) from Griffen (2018 and references therein). The frontal surface area of the animal was assumed to be equal to the submerged part of the total frontal area. The wetted surface area is evidently less than the entire body area but, without a buoyancy analysis, the exact value is difficult to calculate accurately. Because, as stated previously, most of the body of the bear is submerged, instead of estimating the surface for the emerged 3D shape of the back and head (which would be very difficult), a reasonable approximation is to subtract a narrow 2D strip from the total surface area (S). This rectangular strip would be approximately the width of the head of the bear and would span the waterline length (i.e. the total length) of the animal. Thus, for a lack of a better measure, the wetted surface area in m^2 would be approximately:

$$S_{wetted} = 0.09M^{0.67} - head\ width \times waterline\ length \tag{S6.1}$$

Where M is the mass of the polar bear which was equated with the mass of the female polar bear that swam for 9 days (226 kg) described in Durner et al. (2011); head width was assumed to be approximately 20 cm based on the head width of females (Derocher and Stirling, 1998) and the waterline length equal to 2 m. For the frontal surface we calculated the combined area of the vertical cross section areas of the axial body and of the two front legs (Figure S6.3A) using the female bear values from Mathewson and Porter (2013: fig. S3). This area in m^2 is given by summing the area of an ellipse with the area of two equal rectangles respectively:

$$S_{frontal} = 0.51 \times 0.58 \times \pi + 2 \times 0.23 \times 0.46 \tag{S6.2}$$

The 0.51 m value is the ellipse minor-diameter (i.e., the width of the animal) and the 0.58 m value in the above equation is the ellipse major-diameter found by doubling the vertical thickness of the neck (=0.29 m) (Figure S6.3A), assuming the latter to be half the height (=cross sectional ellipse major axis) of the axial body.

For the tortoise we made a geometrical approximation of its shape in the form of a hemisphere of 0.77 m curved carapace measured length (Figure S6.3B), following the dimensions measured in Gerlach et al. (2006). This was used to estimate the radius of the hemisphere as R=0.77/π (m), where the diameter is what we will refer to as the linear body length (L) of the tortoise and is equal to 0.49 m. The waterline height was assumed to be at the mid-height of the carapace i.e. R/2=0.245 m from the top of the carapace to the waterline. The waterline length (L_w) was calculated to be 0.42 m using the formula:

$$L_w = \sqrt{\left(R^2 + \frac{R^2}{4}\right)} \tag{S6.3}$$

The submerged frontal surface area of a hemisphere for which the waterline is on R/2 height from the base was calculated using the formula:

$$S_{frontal} = \frac{\pi}{6}R^2 + \frac{R}{4}L_w \tag{S6.4}$$

Whereas the wetted surface area was estimated using the equation:

$$S_{wetted} = 2\pi R^2 \tag{S6.5}$$

The waterline height in relevance to the hemispheric shape was assumed based on the photograph of a swimming tortoise presented in Gerlach et al. (2006: fig. 3).

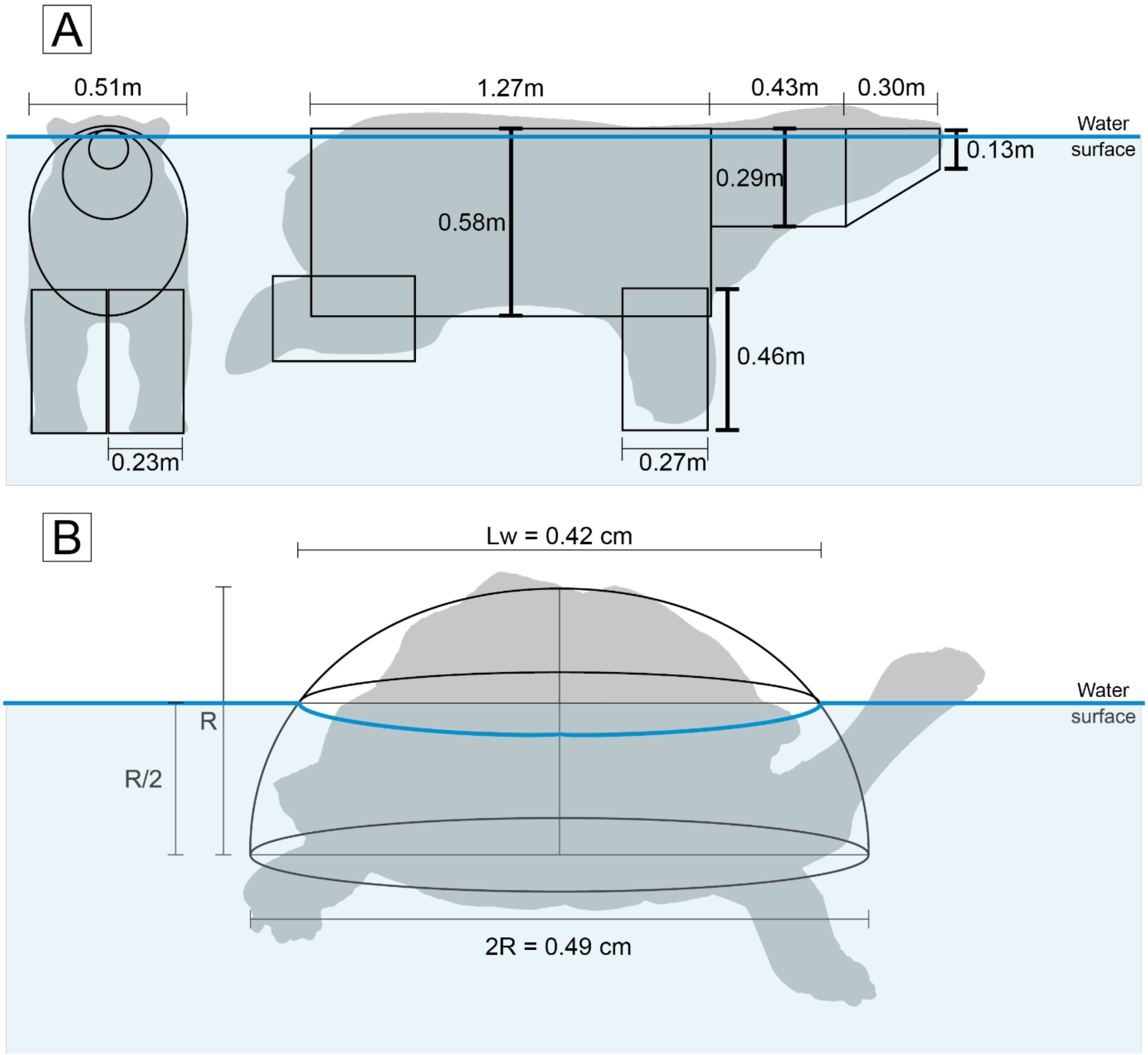

Figure S6.3. A: Geometry used to approximate the dimensions and shape of a polar bear swimming in water in front (left) and body profile (right) views. Dimensions correspond to a female individual based on the values used in Mathewson and Porter (2013: fig. S3). B: Hemispherical geometry constructed using dimensions of *Aldabrachelys gigantea* from Gerlach et al. (2006); immersion depth estimated from photographs presented in the same study. Waterline level was assumed to be at half the height between the base of the hemisphere and its top. Blue line indicates the water surface.

S6.6. Hydrostatic stability tests

In order to assess how well the two dinosaurs could have retain their stability while in water, DMH performed a hydrostatic stability test where the positions of the center of gravity (KG), center of buoyancy (KB) and metacenter (KM), as well as, the metacentric height were estimated (*sensu* Henderson, 2018). For precise definitions of KB, KG, KM and metacentric height see Section S6.1. The metacentric height in particular can serve as a stability index with

which to compare how well two animals can swim relative to each other, especially when it comes to long distances. The results (Figure S6.4) showed that: 1) with regards to the relative position of KG and KB, both our dinosaurs natural pose is in an unstable equilibrium; 2) both animals have negative metacentric heights; and 3) the negative metacentric height is greater for the titanosaur.

It is evident from the above that neither dinosaur would have been very stable in water without some effort from their limbs. However, here we are mainly interested in the relative stability, for comparative reasons. The action of waves hitting the animal from the side could be tolerated up to a point when the animal eventually capsizes (or more precisely a new stable equilibrium position is reached). Hence, we can assume that animals that can tolerate tilting to a greater degree (i.e. are more stable when floating motionless) would also have an advantage when actively swimming (or drifting passively) long distances in the ocean. Thus, we can conclude that the hadrosaur is more stable because: 1) the distance between KB and KG is smaller and closer to a neutral equilibrium situation compared to the titanosaur and 2) the smaller negative metacentric height of *Lambeosaurus* shows that it has greater lateral stability in water than *Rapetosaurus*.

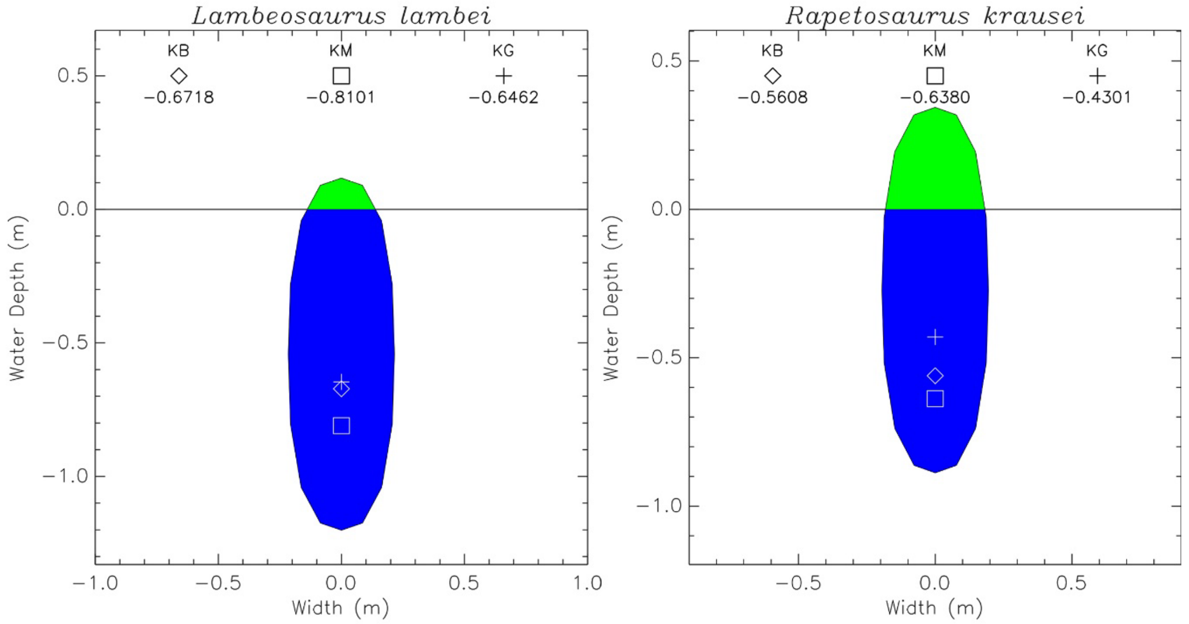

Figure S6.4. Graphical views of the metacenter (KM '□'), center of buoyancy (KB '◊'), and center of gravity (also known as center of mass in some cases) (KG '+') computed from the three-dimensional model of *Lambeosaurus lambei* and *Rapetosaurus krausei* by DMH. A center of gravity above the metacenter indicates an unstable situation. Stated measurements are relative to the water line. Green indicates the 'dry' area above the waterline, while the blue is the 'wet,' immersed portion. The body slice shown is taken from the sacral region.

References

Abbas, U., Khalid, S., Riaz, Z., Zubair, A., Khalid, H., 2021. Development of a Large Angle Stability Tool For The Ships and Boats, in: 2021 International Bhurban Conference on Applied Sciences and Technologies (IBCAST). Presented at the 2021 International Bhurban Conference on Applied

Sciences and Technologies (IBCAST), IEEE, Islamabad, Pakistan, pp. 873–880. https://doi.org/10.1109/IBCAST51254.2021.9393237
Atkinson, J.F., 2022. Pressure, in: Mehner, T., Tockner, K. (Eds.), Encyclopedia of Inland Waters. Academic Press, Oxford, pp. 155–165. https://doi.org/10.1016/B978-012370626-3.00009-0
Barrass, B., Derrett, D.R., 2006. Ship Stability for Masters and Mates, 6th ed. Butterworth-Heinemann.
Biran, A., López-Pulido, R., 2014. Ship hydrostatics and stability, 2nd edition. ed. Elsevier Butterworth-Heinemann, Amsterdam.
Chadwick, D.H., 1991. Elephants: out of time, out of space. National Geographic 179, 2–49.
Curry Rogers, K., Forster, C.A., 2001. The last of the dinosaur titans: a new sauropod from Madagascar. Nature 412, 530–534. https://doi.org/10.1038/35087566
Deeming, D.C., Sibly, R.M., Magole, I.L., 1996. Estimation of the weight and body condition of ostriches (*Struthio camelus*) from body measurements. Vet Rec 139, 210–213. https://doi.org/10.1136/vr.139.9.210
Derocher, A.E., Stirling, I., 1998. Geographic variation in growth of polar bears (*Ursus maritimus*). Journal of Zoology 245, 65–72. https://doi.org/10.1111/j.1469-7998.1998.tb00072.x
Durner, G.M., Whiteman, J.P., Harlow, H.J., Amstrup, S.C., Regehr, E.V., Ben-David, M., 2011. Consequences of long-distance swimming and travel over deep-water pack ice for a female polar bear during a year of extreme sea ice retreat. Polar Biol 34, 975–984. https://doi.org/10.1007/s00300-010-0953-2
Fish, F.E., 1993. Comparison of Swimming Kinematics between Terrestrial and Semiaquatic Opossums. Journal of Mammalogy 74, 275–284. https://doi.org/10.2307/1382382
Gerlach, J., Muir, C., Richmond, M.D., 2006. The first substantiated case of trans-oceanic tortoise dispersal. Journal of Natural History 40, 2403–2408. https://doi.org/10.1080/00222930601058290
Giles, R.V., Evett, J.B., Liu, C., 1995. Schaum's outline of theory and problems of fluid mechanics and hydraulics, 3. ed. ed, Schaum's outline series. McGraw-Hill, New York, NY.
Griffen, B.D., 2018. Modeling the metabolic costs of swimming in polar bears (*Ursus maritimus*). Polar Biol 41, 491–503. https://doi.org/10.1007/s00300-017-2209-x
Grigg, G.C., Kirshner, D., 2015. Biology and evolution of crocodylians. Comstock Publishing Associates a division of Cornell University Press, Ithaca.
Gutarra, S., Rahman, I.A., 2022. The locomotion of extinct secondarily aquatic tetrapods. Biological Reviews 97, 67–98. https://doi.org/10.1111/brv.12790
Henderson, D.M., 2018. A buoyancy, balance and stability challenge to the hypothesis of a semi-aquatic *Spinosaurus* Stromer, 1915 (Dinosauria: Theropoda). PeerJ 6, e5409. https://doi.org/10.7717/peerj.5409
Henderson, D.M., 2014. Duck Soup: The Floating Fates of Hadrosaurs and Ceratopsians at Dinosaur Provincial Park, in: Hadrosaurs, Life of the Past. Indiana University Press, Bloomington, pp. 459–466.
Henderson, D.M., 2004. Tipsy punters: sauropod dinosaur pneumaticity, buoyancy and aquatic habits. Biol. Lett. 271, S180–S183. https://doi.org/10.1098/rsbl.2003.0136
Henderson, D.M., 2003. Effects of stomach stones on the buoyancy and equilibrium of a floating crocodilian: a computational analysis. Can. J. Zool. 81, 1346–1357. https://doi.org/10.1139/z03-122
Henderson, D.M., 1999. Estimating the masses and centers of mass of extinct animals by 3-D mathematical slicing. Paleobiology 25, 88–106. https://doi.org/10.1666/0094-8373(1999)025<0088:ETMACO>2.3.CO;2
Henderson, D.M., Naish, D., 2010. Predicting the buoyancy, equilibrium and potential swimming ability of giraffes by computational analysis. Journal of Theoretical Biology 265, 151–159. https://doi.org/10.1016/j.jtbi.2010.04.007

Mallon, J.C., Henderson, D.M., McDonough, C.M., Loughry, W.J., 2018. A "bloat-and-float" taphonomic model best explains the upside-down preservation of ankylosaurs. Palaeogeography, Palaeoclimatology, Palaeoecology 497, 117–127. https://doi.org/10.1016/j.palaeo.2018.02.010

Mathewson, P.D., Porter, W.P., 2013. Simulating Polar Bear Energetics during a Seasonal Fast Using a Mechanistic Model. PLoS ONE 8, e72863. https://doi.org/10.1371/journal.pone.0072863

McNeel, R., 2020. Rhinoceros (Version 7).

Meijaard, E., 2001. Successful sea-crossings by land mammals; a matter of luck, and a big body. A preliminary and simplified model. Geol. Res. Dev. Centre, Spec. Publ 87–92.

Muybridge, E., 1887. Animal locomotion : An electro-photographic investigation of consecutive phases of animal movements. Lippincott, Philadelphia.

Pagano, A.M., Cutting, A., Nicassio-Hiskey, N., Hash, A., Williams, T.M., 2019. Energetic costs of aquatic locomotion in a subadult polar bear. Mar. Mam. Sci. 35, 649–659. https://doi.org/10.1111/mms.12556

Paul, G.S., 1987. The science and art of restor-ing the life appearance of dinosaurs and their relatives: a rigorous how-to guide, in: Czerkas, S.M., Olson, E.C. (Eds.), Dinosaurs Past and Present. Natural History Museum of Los Angeles County/University of Washington Press, Seattle and Washington, pp. 5–49.

Schmidt-Nielsen, K., 1984. Scaling: Why is Animal Size So Important? Cambridge University Press.
