# Supplementary material for: ENHYDROSS: A New Mechanistic Model Supports the Trans‐Oceanic Dispersal Capability of Terrestrial Vertebrates
Source: Ecol Evol. 2026 Mar 30;16(4):e73280. doi: 10.1002/ece3.73280 (PMC13107292; doi:10.1002/ece3.73280)
Supplement: Supplementary file 7 — Data S7: ece373280‐sup‐0007‐SupplefileS7.pdf. [file ECE3-16-e73280-s007.pdf]

S7 Dinosaur Paleobiogeography case study: Additional Information and results

S7.1. Dinosaur metabolism

Because the ENHYDROSS model assumes that M_T=0, knowing whether dinosaurs were endotherms or ectotherms is not in itself vital for our analyses because the thermogenesis factor will be null either way. On the other hand, metabolic rate estimation is clearly central to the application of ENHYDROSS to dinosaurs. There are two major areas of uncertainty when estimating dinosaurian metabolic rates based on allometric scaling equations: 1) what were the metabolic regimes of various dinosaurian clades? and 2) which equations should we select from the range available for extant groups? First, there is conflicting evidence regarding the thermoregulatory status of hadrosaurs and especially sauropods. For dinosaurs in general, researchers have argued for: 1) ectothermic metabolic rates, that in the case of large dinosaurs resulted in inertial homeothermy ('gigantothermy') (e.g. Gillooly et al., 2006; McNab, 2009b; Paladino et al., 1990; Ruben et al., 2003, 1998, 1996; Seebacher et al., 1999; Spotila et al., 1973; Weaver, 1983); 2) endothermic rates (e.g. Dawson et al., 2020; Eagle et al., 2011; Fisher et al., 2000; Grigg et al., 2022; Köhler et al., 2012; Paul, 2017; Pontzer et al., 2009; Seymour, 2016, 2013; Seymour et al., 2023, 2012) ; or 3) intermediate 'mesothermic' rates (e.g. Grady et al., 2014; Lee, 2015). In addition, different clades, age-groups, and/or size-groups of non-avian dinosaurs might have followed different thermoregulatory strategies and thus some researchers have argued against a single metabolic regime common to all groups (e.g. Chiarenza et al., 2024; Clarke, 2013; Eagle et al., 2015; Herculano-Houzel, 2023; Laskar et al., 2020; Seebacher, 2003; Weaver, 1983; Wiemann et al., 2022). Second, studies of metabolic allometric scaling are themselves a field of intense debate (see reviews and discussion in: Capellini et al., 2010; Hoppeler and Weibel, 2005; Hulbert and Else, 2000; Kleiber, 1961; Kozłowski et al., 2020; Prothero, 2015; Schmidt-Nielsen, 1984; West et al., 1997; West and Brown, 2005; White et al., 2007; White and Marshall, 2023; White and Seymour, 2005), with some studies supporting a power law with an exponent of approximately 2/3 or close to it (e.g. White and Seymour, 2005), or an exponent of (or closer to) 3/4 (Kleiber, 1961; West et al., 1997; West and Brown, 2005), and still others arguing against a meaningfully universal exponent altogether (e.g. Capellini et al., 2010; Kozłowski et al., 2020; White et al., 2007).

Plotted BMRs obtained from the allometric equations for all animals included in our study are shown in figure S7.1. The span of possible non-avian dinosaurian BMRs is 177.8–2551.8 Watts for a 2643 kg *Lambeosaurus* and 203.5–2876.0 Watts for a 3116 kg *Rapetosaurus*. Generally, higher values correspond to endothermic (mammalian or avian) BMRs, whereas lower values correspond to ectothermic (reptilian) BMRs, although there is significant overlap amongst them (Figure S7.1). This reflects the fact that the distinction between endothermic and ectothermic levels of metabolism is not as clear-cut as sometimes generalised.

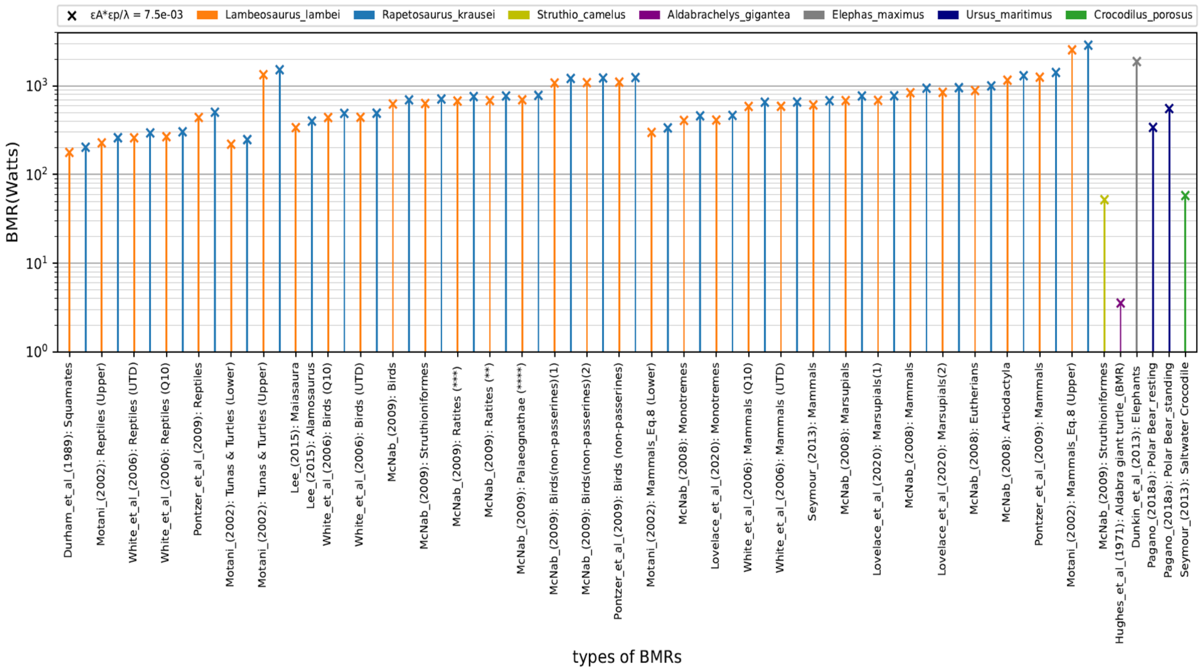

Figure S7.1. Plot showing the BMR (or equivalent SMR for respective ectothermic groups) in Watts for each animal used in this study. For the dinosaurs *Lambeosaurus* and *Rapetosaurus*, an array of BMR formulae from the literature was used to capture the range of possible values (see main text for details). Note the logarithmic scale.

S7.2. Water deprivation limits

Below (Table S7.1), we present the compiled data on water deprivation across a variety of taxa, gathered from the literature. In several cases, it was not possible to determine whether the animals went entirely without water intake or received some water through their food. Nevertheless, due to the scarcity of more precise data, we considered it valuable to include these records.

**Taxon**	**Water deprivation time reported (days)**	**References**
Mouse	6–9	Mazza et al., (2019) and references therein
Darwin's leaf-eared mouse (*Phyllotis darwini*)	mean 41.4	Mazza et al., (2019) and references therein
Olive grass mouse (*Abrothrix olivaceus*)	mean 30.8 (autumn)	Mazza et al., (2019) and references therein
	mean 23.8 (spring)	
Oldfield mouse (*Peromyscus polionotus*)	2.7 (24.5–28 °C)	Prothero (2015) and references therein

Ground squirrel (*Citellus leucurus*)	36 (19–25 °C)	Prothero (2015) and references therein
Dog	11–20	Mazza et al., (2019) and references therein
Merino sheep	5	Mazza et al., (2019) and references therein
Sheep	6–10 (in dry tropical summer)	Mazza et al., (2019) and references therein
	6–8 (in 40 °C daily max temperature)	Prothero (2015) and references therein
Black Bedouin Goat (*Capra hircus*)	4 (without free water; with food access)	(Robertshaw and Dmi'el, 1983)
Human	1–2 (in dry tropical summer)	Mazza et al., (2019) and references therein
	2 (predicted) (in 36 °C daily mean temperature)	Prothero (2015) and references therein
Kangaroo (*Macropus giganteus*)	10–12 (without free water; with food access) (25 °C and 45 °C)	(Dawson et al., 2007)
Kangaroo (*Macropus rufus*)		
Reindeer	2	Mazza et al., (2019) and references therein
Camel	12–15 (in 40 °C daily max temperature)	Prothero (2015) and references therein
	17	Schmidt-Nielsen, (1959)
Cow	3–4 (in 40 °C daily max temperature)	Prothero (2015) and references therein
Black Rhinoceros (*Dicernos bicornis*)	5	Mazza et al., (2019) and references therein
Hartmann's mountain zebras (*Equus zebra hartmannae*)	4	Ward, (2016) page 98

African Elephant	2–4 (with food access?)	Hadjisterkotis, (2012) and references therein
Giraffe	months (without free water; with food access)	Foster and Dagg, (1972) and references therein
Ostrich (*Struthio camelus*)	7 (without free water; with food access) (in 20-29°C)	(Withers, 1983)
	9 (half-grown individuals, without free water; with food access) (in 20-29°C)	(Cloudsley-Thompson and Mohamed, 1967)
Emu (*Dromaius novaehollandiae*)	14-21 (without free water; with food access; drunk dilute sea water) (in 25-45°C)	(Maloney and Dawson, 1998)

Table S7.1. Water privation limits for various mammals and ratite birds. Data for mammals were primarily compiled from Mazza et al. (2019) and Prothero (2015, page 238; table 18.5).

S7.3. Thermoneutrality assumption justified by the Cretaceous 'hothouse' conditions

The optimization of speed in our model depends on the assumption of null thermogenesis (M_T=0). For endothermic animals, this presupposes either thermoneutral conditions in water or complete thermal substitution during swimming. Thus, in our case study of non-avian dinosaurs, whether this assumption holds will largely depend on the sea surface water temperature and to a lesser degree on the capacity of dinosaurs to retain activity-generated heat within the body (i.e. thermal substitution). Whereas we cannot draw on any evidence to support the latter, we can refer to the hothouse conditions that characterized much of the Cretaceous for some limited support of the former argument. For example, for the Late Cretaceous, Pearson et al. (2001) estimated a range of 28–32°C for tropical sea surface temperatures (SST) and O'Connor et al. (2019) estimated 27–37°C for mid to high southern latitude SST. The Cenomanian–Turonian Thermal Maximum (~94.5 Ma) saw average global temperatures of ~28°C (Scotese et al., 2021) and mean SST typically of 35°C and up to a maximum of 38°C (Heimhofer et al., 2018), with occasional SST drops to 32°C. Under such hothouse conditions, ENHYDROSS assumption of null thermogenesis is well justified via near-thermoneutrality, assuming that the body temperatures of our modelled dinosaurs were around 33–38°C, as calculated for some sauropods at least (e.g. Eagle et al. 2011, 2015). If dinosaurs were indeed endothermic, this estimated range is further supported by the body temperature of extant endothermic species like elephants (~36°C) (Benedict and Lee, 1936) and ostriches (~38–39°C) (Withers, 1983). Furthermore, using equation 15 in Kwak et al. (2016), we can calculate the body temperature (T_b) in Celsius of our non-avian dinosaur models using the formula T_b=35.8+0.21*Log_{10}(m), where m is the mass of the animal. The result is an estimated body temperature of 36.5°C for both of our model dinosaurs.

S7.4. Paleogeographic distances

S7.4.1. Estimating Minimum Swimming Distances Along the Albora Route

Figure S7.2 shows the region where time slices were used to measure oceanic gap distances along the Albora route and the intervening islands. For the dataset that includes islands, distances between each island and the two continents were measured, and the longer of these two distances was selected as the minimum swimming distance required for an animal. If more than one island was present along the route, distances between the islands were also measured. If any of these inter-island distances exceeded the distances between the islands and the continents, the longest inter-island distance was instead selected as the minimum swimming distance.

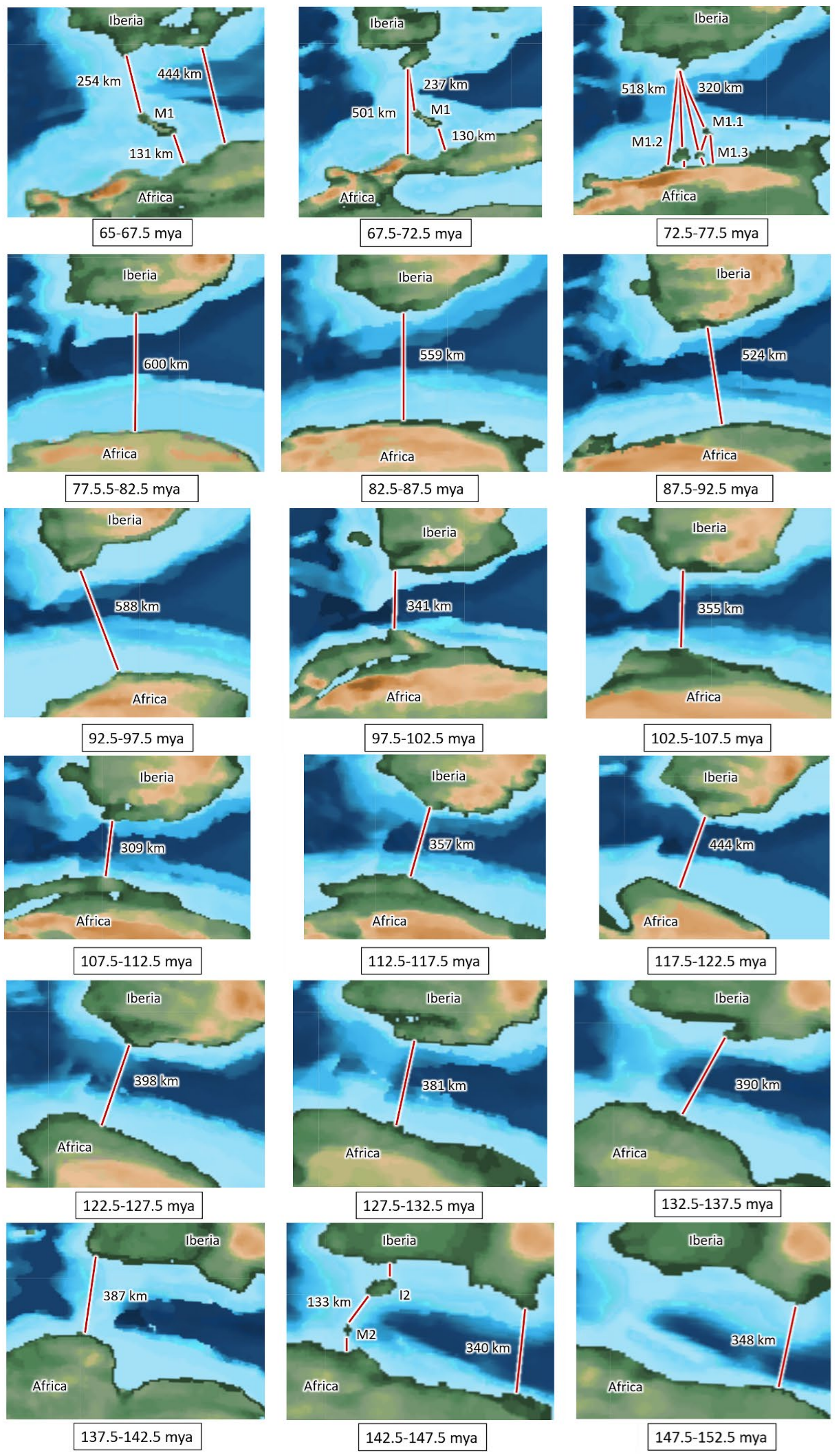

Iberia
254 km
444 km
M1
131 km
Africa
65-67.5 mya
Iberia
237 km
501 km
M1
130 km
Africa
67.5-72.5 mya
Iberia
518 km
320 km
M1.1
M1.2
M1.3
Africa
72.5-77.5 mya
Iberia
600 km
Africa
77.5.5-82.5 mya
Iberia
559 km
Africa
82.5-87.5 mya
Iberia
524 km
Africa
87.5-92.5 mya
Iberia
588 km
Africa
92.5-97.5 mya
Iberia
341 km
Africa
97.5-102.5 mya
Iberia
355 km
Africa
102.5-107.5 mya
Iberia
309 km
Africa
107.5-112.5 mya
Iberia
357 km
Africa
112.5-117.5 mya
Iberia
444 km
Africa
117.5-122.5 mya
Iberia
398 km
Africa
122.5-127.5 mya
Iberia
381 km
Africa
127.5-132.5 mya
Iberia
390 km
Africa
132.5-137.5 mya
Iberia
387 km
Africa
137.5-142.5 mya
Iberia
133 km
I2
M2
340 km
Africa
142.5-147.5 mya
Iberia
348 km
Africa
147.5-152.5 mya

Figure S7.2. Paleomaps showing the shortest distances between Africa and Iberia during the Cretaceous, with and without islands; obtained from GPlates using the Scotese et al. (2024) paleogeography model.

S7.4.2. Alternative paleogeographies

As discussed in the main text, paleogeographic reconstructions can vary substantially between different models and even between versions of the same model (e.g. Scotese, 2016, 2021 vs Scotese et al., 2024). This variation can lead to significant differences in estimated intercontinental distances, which in turn affects paleobiogeographic interpretations (Buffan et al., 2023). To evaluate how sensitive our distance-based conclusions are to model choice, we compared the shortest shore-to-shore distances derived from two different paleogeographic reconstructions: Scotese et al. (2024), which forms the basis of our main analysis, and Cao et al. (2017), an alternative open-access model. The purpose of measuring distances from two different paleogeographies is to illustrate how model choice influences the shortest distances between two shores, and to highlight the model-dependent uncertainty involved when making paleobiogeographic inferences about potential dispersal routes and barriers.

The two models have different temporal resolutions, so for Scotese et al. (2024) we measured distances at intervals of 5 million years, whereas for Cao et al. (2017) we measured distances every 2 Ma, despite the latter model offering a much higher native temporal resolution (less than 1 million years) (<1 Ma). Figure S7.3 shows intercontinental distances drawn at a temporal resolution of 0.5 million years to provide a more accurate representation of key transitional periods. For the case of Cao et al. (2017), intermediate time slices were assumed to retain the values of the previous measured slice, unless a significant qualitative change in landmass configuration occurred, in which case the value was updated to match that of the following measured slice. According to Scotese et al. (2024), islands existed en route during the 66–77.5 Ma and during 142.5–147.5 Ma, whereas for the Cao et al. (2017) model we could identify islands in the region of interest between the interval 94.5–117 Ma and 146.5-147.5 Ma.

The intercontinental distances of Cao et al. (2017) remain substantially higher across most of the Cretaceous, at least when no islands are taken into account. When islands are accounted for, the pattern reverses during the period 94.5–117.5 Ma, with distances measured from Scotese et al. (2024) being 100 km or more longer than those from Cao et al. (2017). Discrepancies between Cao et al. (2017) and Scotese et al. (2024) models are especially pronounced during the 85–95 Ma and 137–148 Ma intervals. By contrast, the 66–77.5 Ma and 98–117.5 Ma intervals show more similarity between models, although Cao et al. (2017) still estimates continental distances to be around 100 km higher. The highest levels of agreement across the two models occur during the 117–137 Ma and 77.5–82.5 Ma intervals.

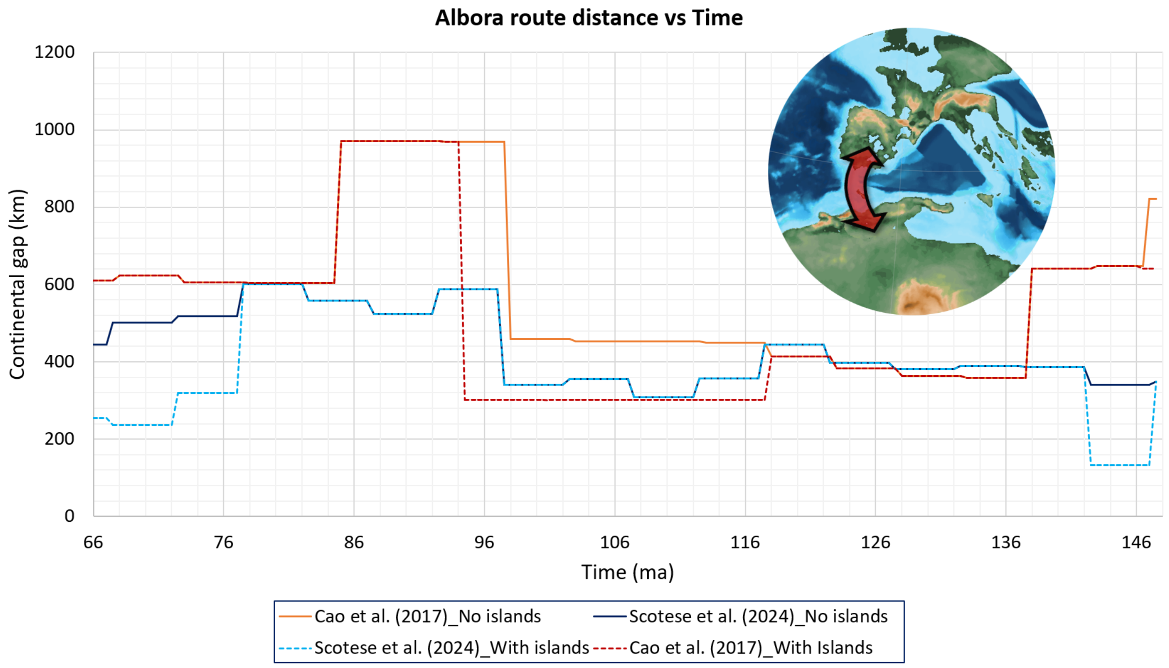

Figure S7.3. Continental gap between Iberia and Africa ("Albora route") for the entire Cretaceous based on two paleogeographic models.

S7.5. Quantifying the uncertainty of dinosaurian BMR

As noted above (Section S7.1), currently we have no way of ascertaining which BMR most accurately reflects the metabolic regimes of our modelled dinosaurs. This is problematic because whether a given distance of ocean could be crossed will ultimately depend on metabolic regime (BMR). Consequently, whether a given dinosaur was, or was not, capable of crossing a given oceanic distance cannot be determined objectively in a simple binary fashion of 'crossing feasible versus crossing not feasible'. Instead of considering this uncertainty as an obstacle, we have converted it into a new type of metric with an accompanying support value, as explained below.

S7.5.1. Plotting BMR as a continuous variable against swimming distance

First, to bypass the problem of having only highly disparate and discontinuous BMR values for our dinosaurs with which to calculate swimming distances, we instead estimated swimming distances corresponding to a large number of BMR increments (here we chose 10 Watts increments), creating a new set of datapoints. We then made a plot of swimming distance against BMR using these data points in order to obtain a pseudo-continuous (dotted/intermittent) curve of the data—for practical purposes a rather smooth curve that will visually reveal the underlying patterns more clearly. While one may argue that this is an unsupported assumption with regards to the plausibility of the existence of intermediate BMRs between the discrete results obtained from the allometric equations (given a constant mass), the justification comes from the existing debate with regards to dinosaur metabolism. It is not our role in this study to give preference to certain BMR values, but rather to accommodate uncertainty. We plotted data in this way for both swimming distances with and without a time limit. The curve formed from the latter data plotting has the shape of an

exponential decay, whereas the curve generated by the former forms an asymmetric wave crest with a right-skewed peak, similar to the one shown in figure S7.4. The curvature depends on the parameters used to estimate the swimming distances (i.e. the sensitivity tests).

Here we are primarily interested in the behaviour of the 'time-limit' curve. The most prominent feature of this curve is the point of inflection (the peak of the curve). The unique BMR value corresponding to the peak of the plot of figure S7.4 is the one that results in the greatest maximum swimming distance (Y_{max*}). Note that the asterisk in Y_{max*} denotes that this only applies to the time-limit case, since greater distances exist for the no time-limit curve. Because Y_{max*} signifies the greatest of all possible maximum swimming distances (Y), resulting from a specific set of parameters (including the set time limit), this amounts to a type of 'optimality' and hence we call this the 'optimal' BMR. This is not to be confused with the concept of optimal speed (U_{opt}) and quantities derived from it such as COT_{min}, maximum distance, or duration. Unlike the latter set of quantities, the adjective 'optimal' does not have any biological meaning, hence the quotation marks. Here, we are operating at a meta-optimization level, spanning a range of possible BMRs, U_{opt}, COT_{min}, etc., parameters. The 'optimal' BMR is the lowest BMR for which the curve with a time limit and the curve without a time limit are identical. Likewise, the Y_{max*} is the greatest swimming distance for which the two curves are identical. For the rest of the data points on the 'time-limit' curve, every given distance value on the y-axis corresponds to a pair of x-axis values, i.e. BMRs (see Figure S7.4 and sections 7.5.2 and 7.5.3).

Lastly, what we defined as BMR (and throughout section S7.5 in general) is not strictly the same as the standard definition of Basal Metabolic Rate, but rather $BMR=M_b+M_T$. This is because, unlike the allometric equations which give an immutable constant metabolic rate for the basal functions of an animal, in plotting such a curve we cannot know whether the x-axis metabolic rate values strictly correspond to M_b or M_b+M_T (i.e. $M_T \neq 0$). This is a direct outcome of equation (19) (or equation 6 if one prefers that version).

Therefore, it is useful to plot the data in this way for visualizing the various trends resulting from the sensitivity tests, including the effects of different time limits, as well as understanding several concepts. However, there is a range of applicability with regards to such a plot. In other words, the plot is only meaningful within a certain range of values (see below).

S7.5.2. Calculating BMR ranges allowing for a successful dispersal

In the previous section, we explained that any swimming distance lower than Y_{max*} always corresponds to a pair of BMR values. If we now equate the swimming distance with the *gap distance* that we want our animal to cross successfully, we can see that each continental gap distance corresponds to a range of possible BMR values. In other words, for a given gap distance Y, there is a minimum and a maximum BMR value that, when inputted into our model, will result in a swimming distance equal to the width of the putative marine area between the continents. Consequently, any BMR value between these extrema will result in higher distances than this gap and any value outside this range will result in lower distances, preventing the animal from crossing the gap distance. With the above reasoning, we have just reformulated the original problem of "*what is the swimming distance given a certain metabolic rate?*" to "*what is the range of metabolic rate that allows a successful crossing of a*

given distance?". Our goal now is to estimate the BMR range values that allow for a successful dispersal for a given distance, herein abbreviated to BRSD.

In order to calculate the pair of BMRs that correspond to the lower and upper limits for a given BRSD, we must rework equations (1), (2), (19) and (21). Depending on whether we are calculating the upper or lower limit of a BRSD, a different approach must be used. The upper limit will correspond to a value that is beyond the 'optimal' BMR and hence would belong to the part of the plot that is not affected by the set time limit (Figure S7.4). This segment of the plot follows the exact same equation as the curve with no time limit (Figure S7.4). Thus, to estimate the BMR that corresponds to the upper limit, we first need to find the equation that connects swimming distance with BMR. To do so, we just need to substitute equations (19) and (21) into equation (1), where we define gap distance to be equal to the maximum swimming distance. We then solve for BMR (=M_b+M_T) to obtain the following equation, valid for cases where the BRSD limit is greater than or equal to the 'optimal' BMR:

$$BMR_{max} = \left(\frac{9}{14\times1000}\frac{Available\ Energy}{Gap\ Distance}\right)^{14/9} \times \left(\frac{10}{9}\frac{\varepsilon_A\varepsilon_p}{\rho\lambda S\widehat{C_d}}\right)^{5/9} \tag{24}$$

Where BMR_{max} is the BRSD upper limit, gap distance is in kilometers (hence the inclusion of the conversion factors in the equation for convenience), and everything else is as stated in the main text.

Similarly, because the lower limit of a BRSD corresponds to a value below the 'optimal' BMR, we must derive the equation for the 'time limit' curve, thereby explicitly incorporating the time constraint into our calculations. To do this we can take the simple definition of speed being equal to distance over time. We set speed=U_{opt}, distance=max swimming distance under a time limit (=gap distance), and time=time limit. Hence, taking the latter relationship, equating with equation (19) and solving for BMR(=M_b+M_T), we get the following equation, valid for cases where the BRSD limit is smaller than or equal to the 'optimal' BMR:

$$BMR_{min} = \left(\frac{1000}{24\times3600}\frac{Gap\ Distance}{Time\ Limit}\right)^{14/5} \times \left(\frac{9}{10}\frac{\rho\lambda S\widehat{C_d}}{\varepsilon_A\varepsilon_p}\right) \tag{25}$$

Where BMR_{min} is the BRSD lower limit and once again we included the conversion units so that gap distance is in kilometers and the time limit is in days. Equation (25) applies for BMR values up to and including the 'optimal' BMR and thus it can be used to estimate the lower limit (BMR_{min}) of a BRSD.

For a given distance, subtracting the minimum from the maximum BMR value, we obtain the number that represents the respective BRSD and whose unit is the same as that of BMR (i.e. Watts). If we want to estimate the exact value of Y_{max*}, we can then equate equations (24) and (25) and solve for distance. The resulting equation (with the conversion units once again included) is then:

$$Max\ Swimming\ Distance_{time\ limit} = Max\ Gap\ Distance\ (= Y_{max}\ *) =$$
$$\left(\frac{Available\ Energy}{14\times100}\frac{\varepsilon_A\varepsilon_p}{\rho\lambda S\widehat{C_d}}\right)^{5/14} \times \left(\frac{24*3600}{1000}\times Time\ Limit\right)^{9/14} \tag{26}$$

For the full derivation of equations (24–26) see Supplementary File S2. Finally, if we substitute Y_{max*} in either of equations (24) or (25), we then obtain the 'optimal' BMR.

S7.5.3. Setting sensible boundaries on the BRSDs

So far, we have been operating in a strictly mathematical realm with regards to equations (24–26). However, living organisms are not mathematical objects and certain realistic boundaries must accompany these equations. For example, the lowest BMR possible is not just any positive infinitesimal number above zero Watts. Instead, the lowermost BMR value comes directly from the smallest value obtained from the plausible allometric equations and we call it SAL for short. In this case, SAL equals 177.8 W for *Lambeosaurus* and 203.5 Watts for *Rapetosaurus*. The distance corresponding to this BMR is herein termed Y_{SAL}. Y_{SAL} is approximately equal to 276 km for *Lambeosaurus* and 274 km for *Rapetosaurus*. Respectively, the largest value from the allometric equations is called LAL and its corresponding swimming distance is called Y_{LAL} (2551.8 W and ~183 km for *Lambeosaurus* and 2876.0 W and ~189 km for *Rapetosaurus*). Together, the range of BMR values obtained from the allometric equations is termed BRAL for short.

If for a distance Y we want to obtain a pair of BMRs corresponding to the limits of a BRSD, we follow the protocol: if for a given distance Y, $Y < Y_{max*}$ and $BMR_Y \geq$ 'Optimal' BMR, the BMR_Y is given by equation (24). This corresponds to the higher end of the BRSD (i.e. BMR_{max}). Otherwise, for a given distance Y, $Y_{SAL} \leq Y < Y_{max*}$ and SAL $\leq BMR_Y \leq$ 'Optimal' BMR, the BMR_Y is given by equation (25) and it represents the lower end of the BRSD. If, on the other hand, we have a distance $Y < Y_{SAL}$ then we conventionally set BMR_Y =SAL for the lower end of the BRSD, instead of using equation (25). In this way, we avoid unrealistically low BMRs that are not included in BRAL. The higher end of the BRSD does not constitute a problem in this case because BMR can be elevated to an arbitrary high level and does not have a known upper limit for our purposes. This is because, as we have already mentioned in section S7.5.1, our BMR is conveniently defined as the sum $M_b + M_T$. Although we conventionally take M_T=0 at thermoneutral conditions (BMR=M_b), this need not always be the case (see also section S7.5.5). Hence, a change in the magnitude of BMR here is justified by accommodating a change in M_T. Therefore, to obtain the upper limit of a BRSD we always use equation (24), regardless of distance Y.

In order to obtain the uppermost metabolic rate (=M_b+M_T) for a given set of distances, we simply substitute the lowest distance (Y_{min}) in equation (24). The resulting BMR is referred to as UPMR for short. This value sets the upper limit for how far the x-axis can be meaningfully extended when plotting a figure like figure S7.4. Generally speaking, UPMRs can be arbitrarily high but the concept of BRSD is meaningless below a certain Y_{min} (and hence above a certain metabolic rate) because: 1) animals have physiological and biomechanical limits and hence their metabolism can be raised up to some limit (which unfortunately we seldomly have the luxury of knowing); 2) the swimming distance becomes so small that it is biogeographically irrelevant; and 3) even if one wants to find the UPMR that corresponds to a very small Y_{min}, there is the risk that the resulting metabolic rate will become unrealistically large (up to infinite), as distance approaches zero due to the distance parameter being positioned in the denominator of equation (24). Thus, the Y_{min} used must have some sensible value (e.g. $Y_{min} \geq$ 50 km) beyond which values are irrelevant for all practical purposes (and to clarify: this depends on the animal we are talking about; for example, 50 km might be a small distance for a non-avian dinosaur, but it might be very large for a small rodent). Here, we are not aware of the upper biological limits of our modelled non-avian dinosaurs, so we cannot set a UPMR based on them. Therefore, the UPMR for each dinosaur was dictated by the smallest Africa–

Iberia gap distance measured (i.e. 133 km), which was sufficiently large to avoid the aforementioned problems.

S7.5.4. Converting BRSDs to percentage support values

Having obtained the BRSDs for every intercontinental distance, we then proceeded in creating a new index ratio. For every intercontinental distance, we divided the overlap between the corresponding BRSD and BRAL by BRAL and then multiplied that ratio by 100%. The resulting percentage is interpreted as a standardized (with respect to BRAL) BMR range corresponding to feasible crossings/successful dispersals and is herein called RBRSD. In equation form this is simply:

$$\mathrm{RBRSD} = \frac{\mathrm{BMR_{max}} - \max(\mathrm{BMR_{min}}, \mathrm{SAL})}{\mathrm{BRAL}} \times 100\%, \ \ \text{if LAL} > \mathrm{BMR_{max}} \quad (27)$$

$$\mathrm{RBRSD} = \frac{\mathrm{LAL} - \max(\mathrm{BMR_{min}}, \mathrm{SAL})}{\mathrm{BRAL}} \times 100\%, \ \ \text{if LAL} \leq \mathrm{BMR_{max}} \quad (28)$$

Where, in the numerator, the value subtracted is the greater of BMR_{min} and SAL. This adjustment ensures that only biologically plausible BMR values are used in the calculation of RBRSDs, and avoids artificially inflated support values due to unrealistically low metabolic rates.

In this way, we can convert the BRSD to something analogous to a support value for a successful crossing, for a given intercontinental gap. The reasoning behind this is the following: given a large span of BMRs (M_b) that allow for a successful crossing for a given intercontinental distance (i.e. a large BRSD), it is more likely that the real BMR (M_b) of a given dinosaur will fall within those limits and hence it will increase the chance that our hypothesis of sea crossing is feasible. The opposite would be true if a crossing is feasible for only a narrow range of BMR values (i.e. small BRSD). The last two statements can be restated as follows: the larger the uncertainty with regards to the metabolism of an animal (BRAL), the broader the range of plausible metabolic rates that may overlap with those permitting a successful crossing for a given distance (BRSD). As we gain more information on the metabolism of an animal, the uncertainty (i.e. the BRAL) shrinks and the overlap between BRAL and BRSD narrows in terms of absolute magnitude. This in turn allows us to be more confident in assessing whether or not the animal can make the crossing by the index of overlap (RBRSD). A detailed example of how the RBRSD values are calculated is provided below (section S7.5.6).

Finally, note that equations (27) and (28) only work if there is an actual overlap between BRSDs and BRAL and this will be visible in the plotted data (as in Figure S7.4). The only case where the overlap can be zero is if the BRAL is restricted below the 'optimal' BMR and the lower end of a BRSD is higher than the respective LAL (i.e. within the region above LAL and 'optimal' BMR, including the latter). In these cases, the RBRSD index is, by convention, set to zero.

S7.5.5. Interpreting BRSD values beyond LAL

BRAL values span what is biologically feasible in terms of actual BMR (M_b); any overlap between BRSDs and BRAL shows the range of values of M_b that the animal can have in order

to make a sea crossing. However, the BRSD is also composed of an unknown contribution of M_T. In our RBRSD index we assume this contribution to be null ($M_T=0$) in order for a meaningful ratio to make sense, although within the overlap region this is not a necessary condition. On the other hand, outside the higher end of BRAL (LAL), this condition ($M_T>0$) is indeed necessary. Therefore, when the upper limit of BRSD exceeds LAL, it indicates that the animal can afford additional cost beyond the standard BMR (i.e. $M_b+M_T > M_b$, hence $M_T>0$) and still be able to make a successful crossing via swimming. In other words, just like the 2.3×BMR enhancement test, values exceeding LAL always correspond to elevated metabolisms due to strenuous activity or thermogenesis (M_T). This is a useful piece of additional information because, currently, our model cannot estimate M_T directly, which is why it relies on the assumption $M_T=0$. Nonetheless, when the upper limit of a BRSD is very high (≫LAL), knowing how much M_T is necessary, due to low temperatures involved in a trans-oceanic journey, becomes less important or potentially even irrelevant. A substantially high upper limit for BRSD indicates that the animal would probably still have the capacity to spend additional metabolic cost and still disperse successfully to the opposite shore for the given distance, even at temperatures below thermoneutrality.

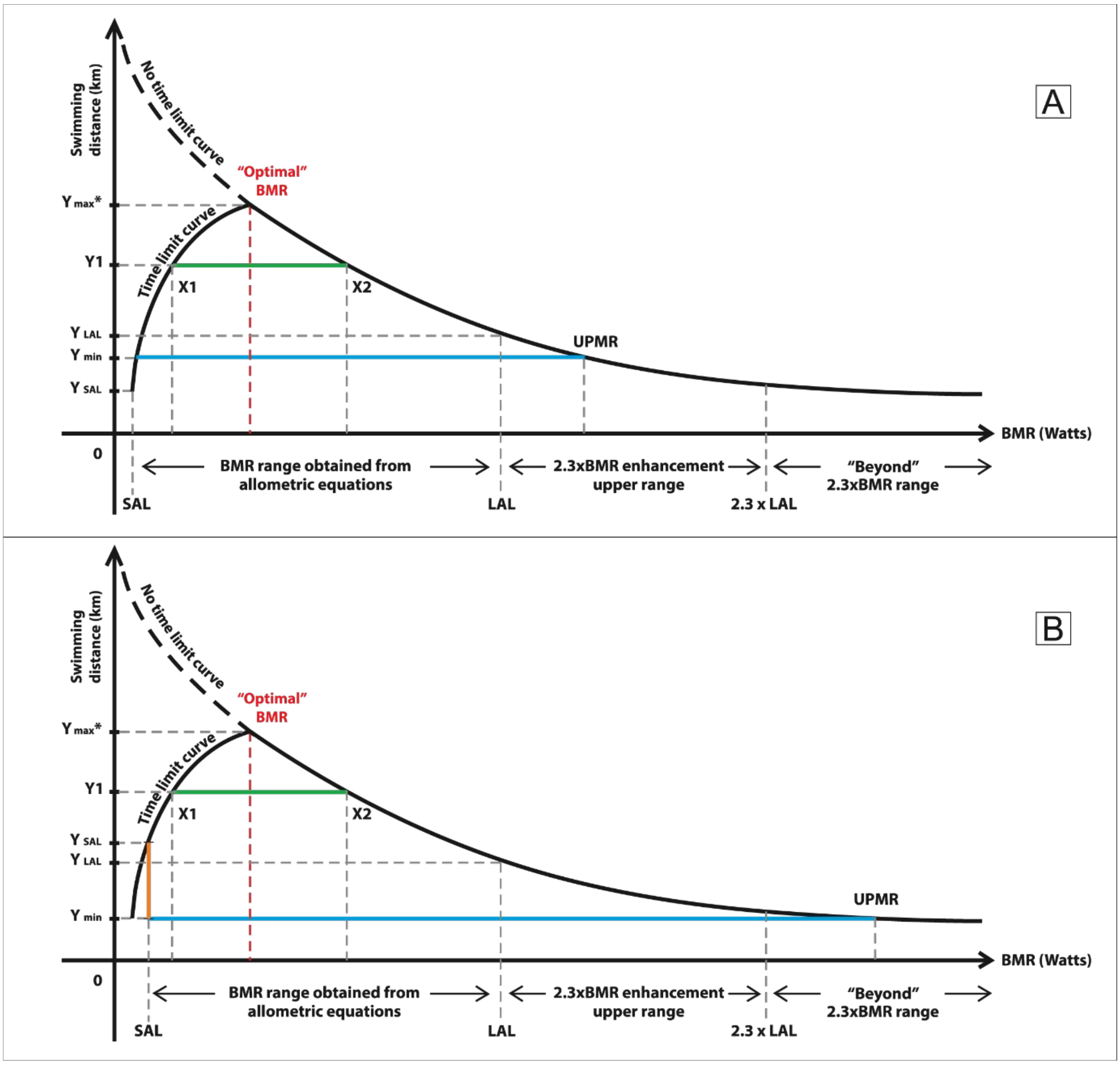

Figure S7.4. Schematic diagrams of the type of plot that results from plotting a continuum of BMR values against swimming distance with and without a time limit. Y_{max}* is the swimming distance that corresponds to the 'optimal' BMR marked by the dashed red line. The asterisk denotes that this applies when a specific time limit is applied only, since without a time limit even largest swimming distances are possible as can be seen from the dashed line of the curve without time limits. SAL and LAL: smallest and largest BMRs from BRAL respectively; Y_{SAL} and Y_{LAL}: distances corresponding to SAL and LAL respectively; Y_{min}: smallest measurable swimming distance to be tested; UPMR: the uppermost metabolic rate (=M_b+M_T) value that corresponds to Y_{min}, beyond which higher metabolic rates are irrelevant (see text for explanation). For a random distance Y1, we observe that the curve with the time limit has two points (X1 and X2) corresponding to two different BMRs. Subtracting X1 from X2 results in a specific BRSD for the distance Y1 represented by the green line. The largest possible BRSD measurable in this way is the one shown by the blue line where one subtracts the BMR_{min} (the smallest BMR that corresponds to Y_{min}) from UPMR. A: In the case where Y_{min} is higher than Y_{SAL}, the lower end of the BRSD can always be estimated using equation 25. B: For a case where Y_{min} is lower than Y_{SAL}, for any distance between and including Y_{min} and Y_{SAL} (orange vertical line) the corresponding BMR=SAL (see text for explanation).

S7.5.6. Worked example of how to calculate BRSDs and RBRSDs

To give an illustrative example, consider our *Lambeosaurus* attempting to swim across a distance of 300 km within a time limit of 14 days. We first find that BRAL = LAL – SAL = 2551.8 W – 177.8 W = 2374 W. Then, using equations (24) and (25), we calculate BMR_{max} = 1183.3 W and BMR_{min} = 223.6 W, respectively. BMR_{min} is greater than SAL this time so subtracting the former from LAL gives us the BRSD = 959.7 W. Since LAL > BMR_{max} in this case, we compute the RBRSD using equation (27):

RBRSD = (959.7 W / 2374 W) × 100% = 40.4%.

Now consider that instead of 300 km, our dinosaur attempts to cross a 150 km barrier. The BRAL remains the same, and using equations (24) and (25), we find BMR_{max} = 3478.4 W and BMR_{min} = 32.1 W. However, the latter yields an unrealistically low metabolic rate for such a large animal, and so we must intervene by setting BMR_{min} = SAL = 177.8 W. We then find BRSD = BMR_{max} – SAL = 3300.6 W. This time, since LAL < BMR_{max}, the RBRSD is given by equation (28):

RBRSD = (2551.8 W – 177.8 W) / 2374 W × 100% = 100%.

S7.6. ENHYDROSS additional results

S7.6.1. Results of the 7-day water deprivation limit

The results of the analysis of the discrete dataset for the 7-day water-privation limit are shown in figure S7.5. Note that the 7 days cut-off for water privation tolerance applies only to the dinosaurs, the elephant and the ostrich. The polar bear, the crocodile and the tortoise can last longer without water so this cut-off does not represent water-privation tolerance for

them but merely a time limit. It is clear that very few periods and very few metabolic regimes allow for a crossing of the Albora route if this time limit represents reality. Notably, the Late Cretaceous period of 72.5-66 ma, is not crossable by any of the ectothermic BMRs bar the upper tuna and turtles one, even with islands present. Moreover, even with the endothermic BMRs (avian and mammalian) the preferred parameters do not allow for a crossing during this time except a subset of the highest BMRs. Instead, crossing during this period is favored by sensitivity tests like the 2.3xBMR and the 'wetted surface area' ones which support higher speeds. By contrast, for the earliest Cretaceous (142.5-147.5 ma) most of the metabolic regimes allow for a successful crossing of the distance when islands are present, as is the case with the 14-day limit scenario.

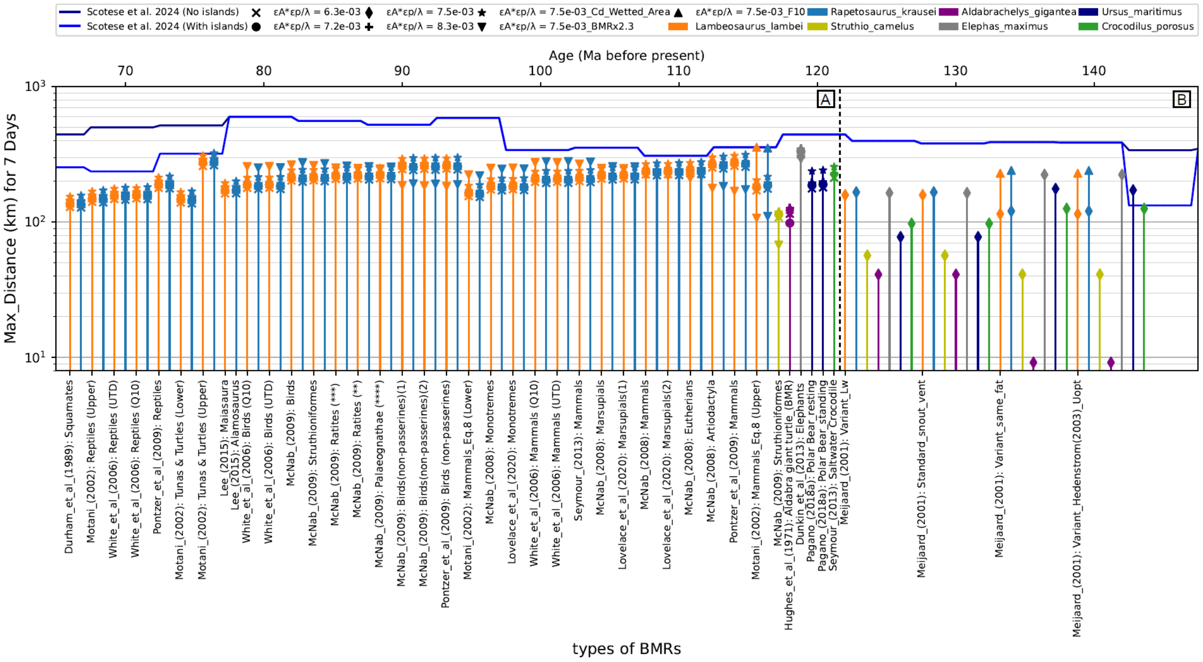

Figure S7.5. ENHYDROSS model's estimated maximum swimming distance (km) per taxon per BMR type corresponding to a duration of 7 days (shown to the left) and results from the Meijaard models for comparison (shown to the right). Also shown are the Albora route distances measured using Scotese et al. (2024) paleogeographies. Note the logarithmic scale. The description of Meijaard's model variants follows that of figure 3 of the main paper.

S7.6.2. Swimming-related differences between hadrosaurs and titanosaurs

Biomechanical arguments, as well as the anatomy of their limbs, suggest that hadrosaurs were probably more efficient swimmers than titanosaurs. For one, we have calculated the negative metacentric height in the *Lambeosaurus* model and its absolute value is smaller than that of *Rapetosaurus,* indicating that the former has greater lateral stability in water (Supplementary File S6: Figure S6.4). In addition, hadrosaurs could have increased their swimming efficiency by using their hind limbs alone while keeping their forelimbs folded underneath their sternum. This might have reduced a hadrosaur's ability to stabilize while in water to some degree, but this would be outweighed by the fact that bipedal swimming is more efficient than quadrupedal swimming because of the interference drag involved in the latter (see Supplementary File S3). Thus, hadrosaurs could potentially have achieved greater

propulsive efficiency and probably maintained lateral stability better than the obligate quadrupedal titanosaurs.

Good paddlers tend to have large and poorly streamlined triangular-shaped feet with interdigital webbing or fringed hair to increase area for thrust, and their limbs have relatively narrow connections to the body in order to allow the propulsor to oscillate at high velocities to increase the momentum to be transferred from the appendage to the environment (Fish, 1994; Webb, 1988). As evidenced by body fossils and footprints, the hadrosaurian hind limb plantar surface was more triangular than the rounded pes with an enlarged 'heel' pad of sauropods (Currie et al., 2003; Jannel et al., 2022; Vila et al., 2013); although the propulsive effect of this difference alone may not be that great overall. In addition, the shape of the hadrosaur's hindlimb is closer to a drum-stick compared to the elephantine pillar-like limbs of titanosaurs and large sauropods in general (e.g. see discussion and restorations in Paul, 1987), further increasing their propulsive difference by allowing the hadrosaur to be more swift and agile in its movements. In other words, the stroke frequency of sauropods is expected to have been lower compared to that of hadrosaurs. This is consistent with the lower terrestrial locomotory speeds of sauropods (see Paul, 1987), although this alone would not necessarily be reflected in the relationship of the swimming speeds of titanosaurs and hadrosaurs. This is because displacing a large amount of water at slow speeds is considered more efficient than accelerating a small amount of water faster (Alexander, 2006, page 250); the reason why elephants are adept swimmers might be due to their relatively large plantar surfaces. For similar reasons, the larger plantar surface area of a sauropod may have been a more effective propulsor than that of the hadrosaur. It could thus be postulated that during swimming, it is likely that the columnar hindlimbs of sauropods would have behaved functionally and kinematically in a manner similar to those of elephants.

On the other hand, we have to account for the possible higher drag force during certain paddling phases expected in graviportal animals. Specifically, during the recovery phase, the foot is repositioned forwardly while minimizing reverse thrust (i.e. drag) (Fish, 1994). To do so efficiently, a good paddler will reduce the effective paddle area by adduction, plantarflexion, or by feathering of the distal limb elements (e.g. hands, feet, or digits) (Fish, 1994). Because of the rigidity of the ankle articulation in sauropods (e.g. see the non-flexibility of the sauropodan autopodium Jannel et al., 2022), a feature shared with elephants but not hadrosaurs (Paul, 1987), a small hydrodynamic advantage during the recovery stroke should be expected for hadrosaurs.

A related issue concerns whether hadrosaurs or titanosaurs used a 'hybrid' locomotion style, i.e. swimming via paddling as well as undulation of the tail, either under certain conditions or habitually whenever they found themselves in water. For example, crocodylians and lizards use foot paddling as well as axial undulations during surface and underwater swimming, whereas they switch to fully axial undulations when moving from moderate to maximal speeds (Sereno et al., 2022 and references therein). Muskrats are an example of a mammalian semi-aquatic paddler that also undulates its laterally compressed and keeled tail when moving in water: however, the thrust produced by the tail is only 1.4% of the total thrust (Fish, 1982; Fish et al., 2021). The tails of swimming tetrapods are either tapered, keeled, paddle-like, or lunate (Fish et al., 2021). Similar to limb paddles, an effective propulsive tail would generate thrust by accelerating a large mass of water adjacent to the body at low speeds, but tapered tails do not optimize this process (Fish et al., 2021). With the exception of *Spinosaurus* (Ibrahim et al., 2020; Sereno et al., 2022), most non-avian dinosaurs

had thick, tapering tails with rounded cross-sections (Fish et al., 2021; Naish, 2024), leading to a progressive reduction in momentum transfer towards the tail's distal end when undulating, diminishing propulsive efficiency (Fish, 1994; Fish et al., 2021). For this reason, the long, whip-like distal ends of sauropodan tails were almost certainly inefficient at producing thrust relative to their overall body size. Even if the proximal ends of the tails of sauropods were deep and narrow, the short transverse processes of that region indicate weaker caudal muscles than crocodylians and other aquatic reptiles: these features are unlike the long transverse processes expected from tails adapted for caudal undulations during swimming (Bakker, 1971; Coombs, 1975), further indicating their inefficiency as propulsion organs. Hadrosaurian tails, by contrast, were laterally compressed (Ostrom, 1964), which at first glance suggest a potentially powerful propulsive organ. However, these tails were stiffened by ossified tendons, albeit primarily in the dorsoventral direction, while lateral movement was allowed to some degree (Weishampel and Horner, 1990). This would render their tails as inefficient in producing thrust from lateral undulations or at least not giving them any hydrodynamic advantage. Nonetheless, extant taxa demonstrate that caution is warranted when inferring the swimming abilities of extinct forms. For example, kangaroos can swim well with a hybrid swimming mode, using their front and hind-limbs ipsilaterally together with undulations of their tails (Wilson, 1974), although we do not know how much their tail contributes to the overall thrust. This is counter-intuitive and surprising when one considers that they are cursorial animals with tails that move primarily in the vertical plane when locomoting on land via jumping, and they display no clear morphological adaptations for swimming. The same point can be made for other cursorial terrestrial animals such as cervids (e.g. Krefting, 1974; Leblond et al., 2016; Quigley and Moffatt, 2014) which have very thin limbs with relatively small plantar surfaces but are adept swimmers. This demonstrates that swimming gaits and overall anatomy do not necessarily correlate in a predictable way. For the moment we can only speculate whether dinosaurs (at least as far as hadrosaurs and titanosaurs are concerned) were using a form of hybrid swimming locomotion, and how much it would have affected their swimming capabilities. In all likelihood, they did oscillate their tails while swimming, although the effect would probably have been minor compared to their main propulsive thrust force coming from limb paddling.

Some have proposed that some species of hadrosaurs were semi-aquatic or at least spent time near-water environments (Ostrom, 1964; Weishampel and Horner, 1990). Although these ideas were largely abandoned after dinosaurs were re-envisioned as fully terrestrial animals (Naish, 2024 and references therein), some degree of aquaticism among hadrosaurs has been resurrected more recently as a result of other lines of evidence. Butler and Barrett (2008) found that hadrosaurs were positively associated with marine sediments which may be related to a proclivity to inhabit coastal regions. This is further supported by the inferred habitat association of hadrosaurids (*Edmontosaurus* sp.) with lowland deltaic environments, at least in the Prince Creek Formation of northern Alaska, as indicated by Fiorillo et al. (2016). Furthermore, Kobayashi et al. (2019) inferred that the ancestral habitat environments of Hadrosaurinae, Lambeosaurinae (as defined therein), as well as early-diverging hadrosaurids, were marginal marine (as opposed to inland) in four out of five models tested. This could indicate that groups such as lambeosaurines preferred near-water environments, which could mean that swimming was part of their ecology. That said, caution is warranted, as not all studies agree on the habitat preferences of hadrosaurid clades. In contrast to the above, the findings of Vázquez López et al. (2025) concerning Ibero-Armorican hadrosauroids showed a clear preference for inland environments. As for titanosaurs, Mannion and Upchurch (2010)

found that they display a strong association with inland habitats, a finding that is also supported by the more recent study of Vázquez López et al. (2025), which focused exclusively on Ibero-Armorican dinosaurs. This indirectly suggests that swimming did not play a regular role in titanosaur ecology. Although our study does not provide direct evidence for aquatic habits in either species, it indirectly supports the notion that hadrosaurs were likely more capable and efficient swimmers than titanosaurs, although both may have been able to locomote in water to some extent.

S7.6.3. Mass effect on the swimming differences of the two dinosaurs

The reason why the hadrosaur outperforms the titanosaur in terms of U_{opt} in all but the wetted-surface area test is related to their mass. Likewise, the reason why the various tests when a time limit is imposed, do not show one animal or the other to be performing better in terms of swimming distance, is also heavily dependent on their mass. Aside from the effect on the different fat mass which is derived from the body mass, their body masses determine their BMR and thus their BMR is not equal for a given allometric equation.

In figure S7.6, the U_{opt} and COT_{min} of the two dinosaurs against BMR is shown for the preferred parameters and the wetted surface area test. Although the trendlines show that the hadrosaur is always faster and more economical (less COT_{min}) than the titanosaur, when inspecting the points of intersection of every pair of vertical lines (corresponding to each animal's BMR for the same allometric equation) with the trendlines (representing equivalent sensitivity tests) we can see that this is no longer the case with regards to U_{opt}. The titanosaur is faster under the wetted surface area test precisely because of the difference in BMR between the two animals, which is a consequence of their different mass. In figure S7.7, we see that by removing the difference in their mass (now instead of a pair of vertical lines for each allometric equation we have only one vertical line corresponding to a single BMR and mass), the hadrosaur is always more economical as well as faster than the titanosaur. This is the case because now for each BMR, the points of intersection of the corresponding vertical line with the trendlines, will be higher for the hadrosaur since the trendlines of the latter are always higher than their equivalent for the titanosaur.

The same applies when comparing the results for the swimming distances of the two dinosaurs. First, by looking at figure S7.8, one can see that under no time limit, the trendlines representing sensitivity tests for the titanosaur are always above their equivalent trendlines for the hadrosaur. For this case, even with the mass difference between the two animals, the titanosaur always swims longer distances for each BMR equation. On the other hand, under a time limit, no animal seems to be outperforming the other under all cases (Figure S7.8). However, once the masses of the two animals are equalized (Figure S7.9), the hadrosaur always outperforms the titanosaur.

When removing the effect of different masses, swimming durations become identical for the two animals because, as we have mentioned in the main text (Section 2.1.7), swimming durations are determined solely by the available energy (fat mass) and BMR. Since by equating masses, we equalize BMRs as well as fat masses, the swimming durations for each dinosaur also become equal.

By eliminating the effect of different masses, a more accurate comparison between the two non-avian dinosaurs becomes possible. This comparison reveals why the hadrosaur is a superior swimmer in terms of speed, efficiency, and traveling distance capacity. However, such comparison is usually reserved for studying aquatic animals where hydrodynamic efficiency is affected by far smaller anatomical details compared to two terrestrial animals that are vastly different in terms of bauplan and mass, like our non-avian dinosaurs. In our case, having different mass is ‘part of the equation’ to begin with, so we can justify our comparison (see above section S7.6.2 and main text section 4.2.1) to some degree without being obliged to resort to presenting the isolated effect of hydrodynamic parameters alone.

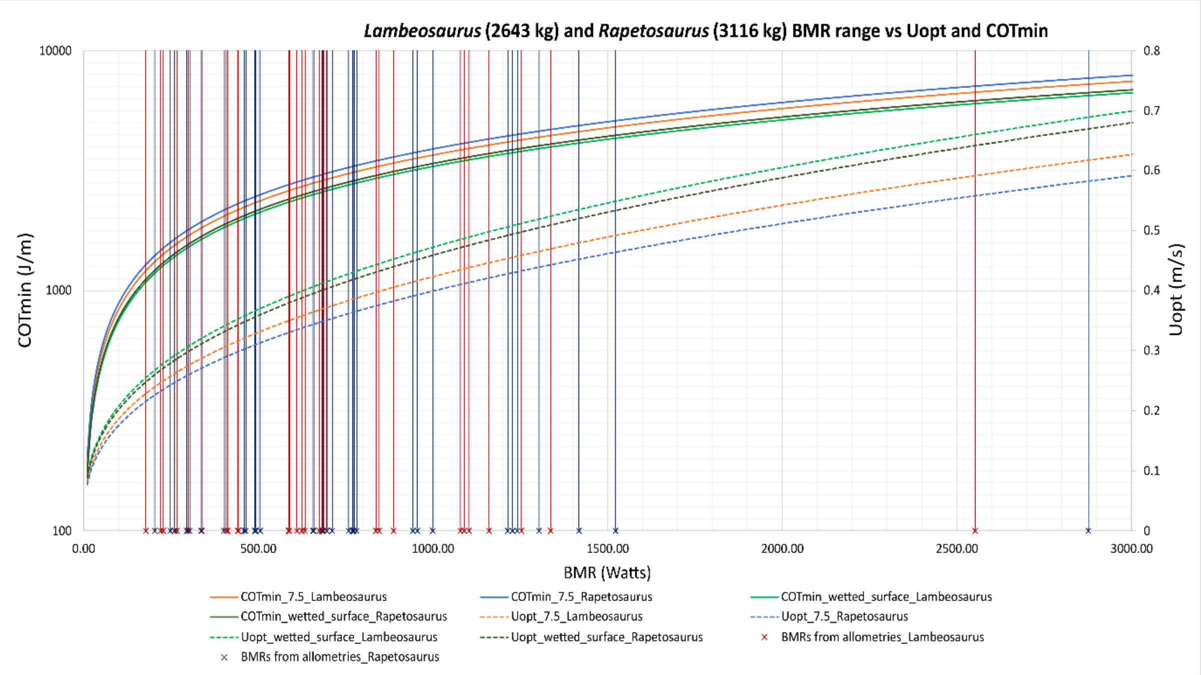

Figure S7.6. Trend curves showing how U_{opt} and COT_{min} vary with BMR for *Lambeosaurus lambei* and *Rapetosaurus krausei* if the effect of different masses is eliminated. Only two of the sensitivity tests are presented per variable, for visual clarity. Thin red vertical lines corresponding to BMRs from allometric equations (for both *Lambeosaurus* and for *Rapetosaurus* weighing 2643 kg), are drawn to enable visual inspection of the points of intersection with the trend curves.

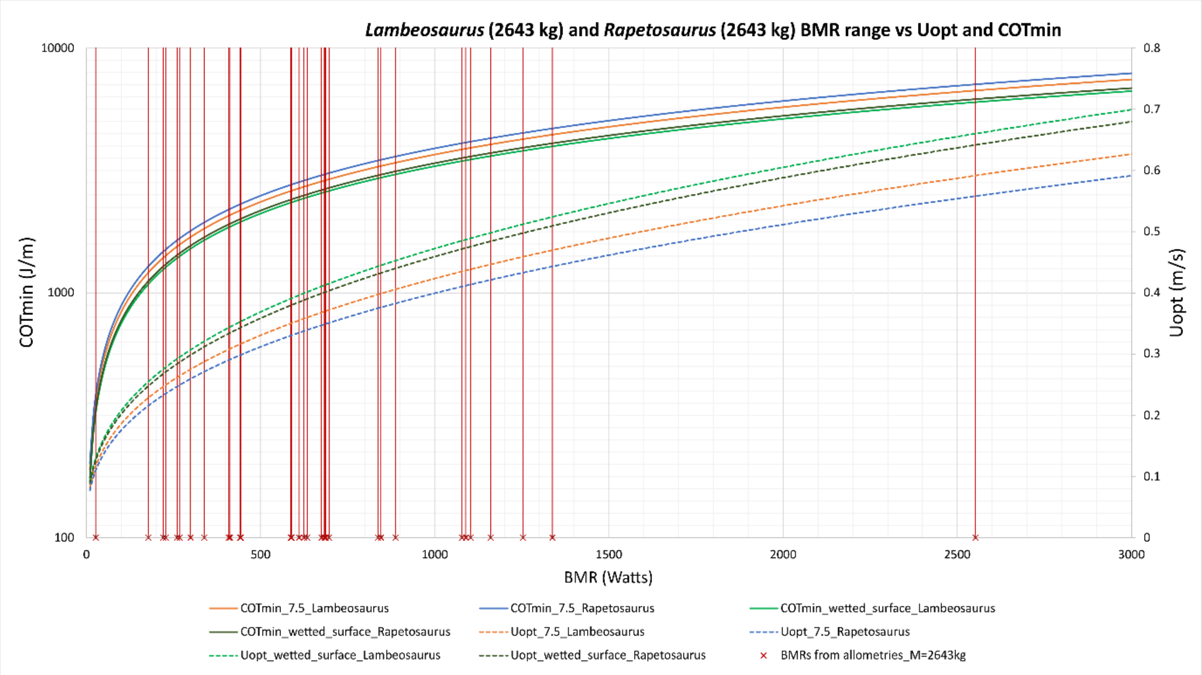

Figure S7.7. Trend curves showing how U_{opt} and COT_{min} vary with BMR for *Lambeosaurus lambei* and *Rapetosaurus krausei* if the effect of different masses is eliminated. Only a subset of the sensitivity tests are presented, for visual clarity. Thin red vertical lines corresponding to BMRs from allometric equations (for both *Lambeosaurus* and for *Rapetosaurus* weighing 2643 kg), are drawn to enable visual inspection of the points of intersection with the trend curves.

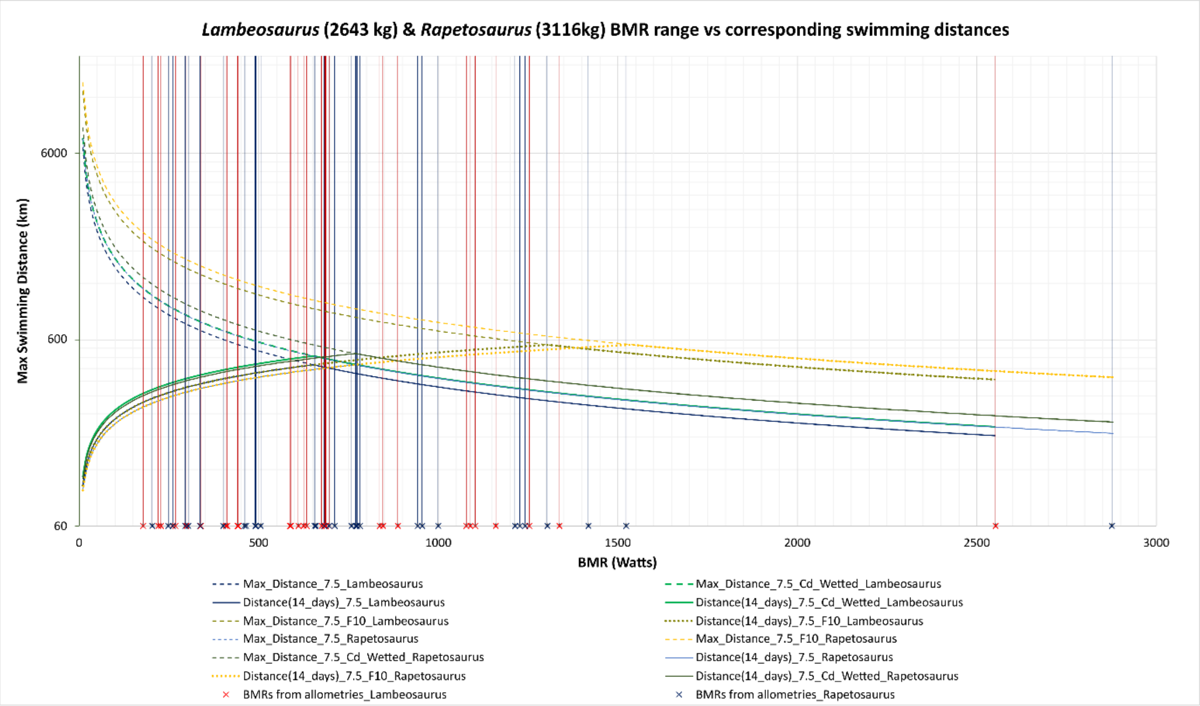

Figure S7.8. Trend curves showing how swimming distance varies with BMR for *Lambeosaurus lambei* and *Rapetosaurus krausei*. Only three of the sensitivity tests are presented, for visual clarity. Thin vertical lines corresponding to BMRs from allometric equations (red for

Lambeosaurus and dark blue for *Rapetosaurus*), are drawn to enable visual inspection of the points of intersection with the trend curves.

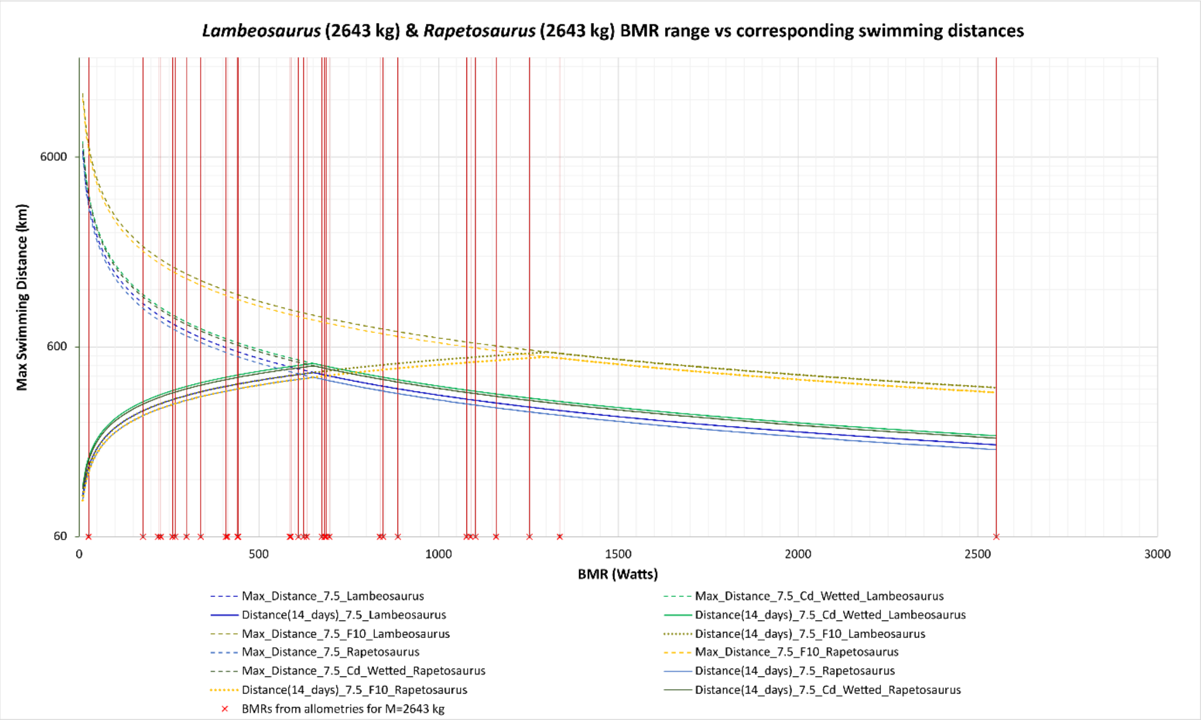

Figure S7.9. Trend curves showing how swimming distance varies with BMR for *Lambeosaurus lambei* and *Rapetosaurus krausei* if the effect of different masses is eliminated. Only three of the sensitivity tests are presented, for visual clarity. Thin red vertical lines corresponding to BMRs from allometric equations (for both *Lambeosaurus* and for *Rapetosaurus* weighing 2643 kg), are drawn to enable visual inspection of the points of intersection with the trend curves.

References

Alexander, R.M., 2006. Principles of animal locomotion, Princeton Paperbacks. Princeton University Press, Princeton.

Bakker, R.T., 1971. Ecology of the Brontosaurs. Nature 229, 172–174. https://doi.org/10.1038/229172a0

Benedict, F.G., Lee, R.C., 1936. Studies on the Body Temperatures of Elephants. National Academy of Sciences 22, 405–408.

Buffan, L., Jones, L.A., Domeier, M., Scotese, C.R., Zahirovic, S., Varela, S., 2023. Mind the uncertainty: Global plate model choice impacts deep-time palaeobiological studies. Methods in Ecology and Evolution 00, 1–13. https://doi.org/10.1111/2041-210X.14204

Butler, R.J., Barrett, P.M., 2008. Palaeoenvironmental controls on the distribution of Cretaceous herbivorous dinosaurs. Naturwissenschaften 95, 1027–1032. https://doi.org/10.1007/s00114-008-0417-5

Cao, W., Zahirovic, S., Flament, N., Williams, S., Golonka, J., Müller, R.D., 2017. Improving global paleogeography since the late Paleozoic using paleobiology. Biogeosciences 14, 5425–5439. https://doi.org/10.5194/bg-14-5425-2017

Capellini, I., Venditti, C., Barton, R.A., 2010. Phylogeny and metabolic scaling in mammals. Ecology 91, 2783–2793. https://doi.org/doi:10.1890/09-0817.1

Chiarenza, A.A., Cantalapiedra, J.L., Jones, L.A., Gamboa, S., Galván, S., Farnsworth, A.J., Valdes, P.J., Sotelo, G., Varela, S., 2024. Early Jurassic origin of avian endothermy and thermophysiological diversity in dinosaurs. Current Biology 34, 2517-2527.e4. https://doi.org/10.1016/j.cub.2024.04.051

Clarke, A., 2013. Dinosaur Energetics: Setting the Bounds on Feasible Physiologies and Ecologies. The American Naturalist 182, 283–297. https://doi.org/10.1086/671259

Cloudsley-Thompson, J.L., Mohamed, E.R.M., 1967. Water Economy of the Ostrich. Nature 216, 1040–1040. https://doi.org/10.1038/2161040a0

Coombs, W.P., 1975. Sauropod habits and habitats. Palaeogeography, Palaeoclimatology, Palaeoecology 17, 1–33. https://doi.org/10.1016/0031-0182(75)90027-9

Currie, P., Badamgarav, D., Koppelhus, E., 2003. The First Late Cretaceous Footprints from the Nemegt Locality in the Gobi of Mongolia. Ichnos 10, 1–13. https://doi.org/10.1080/10420940390235071

Dawson, R.R., Field, D.J., Hull, P.M., Zelenitsky, D.K., Therrien, F., Affek, H.P., 2020. Eggshell geochemistry reveals ancestral metabolic thermoregulation in Dinosauria. Sci. Adv. 6, eaax9361. https://doi.org/10.1126/sciadv.aax9361

Dawson, T.J., Blaney, C.E., McCarron, H.C.K., Maloney, S.K., 2007. Dehydration, with and without heat, in kangaroos from mesic and arid habitats: different thermal responses including varying patterns in heterothermy in the field and laboratory. J Comp Physiol B 177, 797–807. https://doi.org/10.1007/s00360-007-0176-1

Eagle, R.A., Enriquez, M., Grellet-Tinner, G., Pérez-Huerta, A., Hu, D., Tütken, T., Montanari, S., Loyd, S.J., Ramirez, P., Tripati, A.K., Kohn, M.J., Cerling, T.E., Chiappe, L.M., Eiler, J.M., 2015. Isotopic ordering in eggshells reflects body temperatures and suggests differing thermophysiology in two Cretaceous dinosaurs. Nat Commun 6, 8296. https://doi.org/10.1038/ncomms9296

Eagle, R.A., Tütken, T., Martin, T.S., Tripati, A.K., Fricke, H.C., Connely, M., Cifelli, R.L., Eiler, J.M., 2011. Dinosaur Body Temperatures Determined from Isotopic (^{13}C- ^{18}O) Ordering in Fossil Biominerals. Science 333, 443–445. https://doi.org/10.1126/science.1206196

Fiorillo, A.R., McCarthy, P.J., Flaig, P.P., 2016. A multi-disciplinary perspective on habitat preferences among dinosaurs in a Cretaceous Arctic greenhouse world, North Slope, Alaska (Prince Creek Formation: lower Maastrichtian). Palaeogeography, Palaeoclimatology, Palaeoecology, Selected papers based on Geological Society of America, Annual Meeting, Theme Session 241, Ancient Polar Ecosystems and Climate History in Deep Time, Denver, Colorado, USA, 30 October 2013. 441, 377–389. https://doi.org/10.1016/j.palaeo.2015.07.024

Fish, F.E., 1994. Influence of Hydrodynamic-Design and Propulsive Mode on Mammalian Swimming Energetics. Aust. J. Zool. 42, 79. https://doi.org/10.1071/ZO9940079

Fish, F.E., 1982. Function of the Compressed Tail of Surface Swimming Muskrats (*Ondatra zibethicus*). Journal of Mammalogy 63, 591–597. https://doi.org/10.2307/1380263

Fish, F.E., Rybczynski, N., Lauder, G.V., Duff, C.M., 2021. The Role of the Tail or Lack Thereof in the Evolution of Tetrapod Aquatic Propulsion. Integrative and Comparative Biology 61, 398–413. https://doi.org/10.1093/icb/icab021

Fisher, P.E., Russell, D.A., Stoskopf, M.K., Barrick, R.E., Hammer, M., Kuzmitz, A.A., 2000. Cardiovascular Evidence for an Intermediate or Higher Metabolic Rate in an Ornithischian Dinosaur. Science 288, 503–505. https://doi.org/10.1126/science.288.5465.503

Foster, J.B., Dagg, A.I., 1972. Notes on the biology of the giraffe. African J Ecol 10, 1–16. https://doi.org/10.1111/j.1365-2028.1972.tb00855.x

Gillooly, J.F., Allen, A.P., Charnov, E.L., 2006. Dinosaur Fossils Predict Body Temperatures. PLoS Biol 4, e248. https://doi.org/10.1371/journal.pbio.0040248

Grady, J.M., Enquist, B.J., Dettweiler-Robinson, E., Wright, N.A., Smith, F.A., 2014. Evidence for mesothermy in dinosaurs. Science 344, 1268–1272. https://doi.org/10.1126/science.1253143

Grigg, G., Nowack, J., Bicudo, J.E.P.W., Bal, N.C., Woodward, H.N., Seymour, R.S., 2022. Whole-body endothermy: ancient, homologous and widespread among the ancestors of mammals, birds and crocodylians. Biological Reviews 97, 766–801. https://doi.org/10.1111/brv.12822

Hadjisterkotis, E., 2012. The arrival of elephants on the island of Cyprus and their subsequent accumulation in fossil sites, in: Aranovich, M., Dufresne, O. (Eds.), Elephants: Ecology, Behavior and Conservation. Nova Science Publishers, Inc.

Heimhofer, U., Wucherpfennig, N., Adatte, T., Schouten, S., Schneebeli-Hermann, E., Gardin, S., Keller, G., Kentsch, S., Kujau, A., 2018. Vegetation response to exceptional global warmth during Oceanic Anoxic Event 2. Nat Commun 9, 3832. https://doi.org/10.1038/s41467-018-06319-6

Herculano-Houzel, S., 2023. Theropod dinosaurs had primate-like numbers of telencephalic neurons. J Comp Neurol 531, 962–974. https://doi.org/10.1002/cne.25453

Hoppeler, H., Weibel, E.R., 2005. Scaling functions to body size: theories and facts. Journal of Experimental Biology 208, 1573–1574. https://doi.org/10.1242/jeb.01630

Hulbert, A.J., Else, P.L., 2000. Mechanisms Underlying the Cost of Living in Animals. Annu. Rev. Physiol. 62, 207–235. https://doi.org/10.1146/annurev.physiol.62.1.207

Ibrahim, N., Maganuco, S., Dal Sasso, C., Fabbri, M., Auditore, M., Bindellini, G., Martill, D.M., Zouhri, S., Mattarelli, D.A., Unwin, D.M., Wiemann, J., Bonadonna, D., Amane, A., Jakubczak, J., Joger, U., Lauder, G.V., Pierce, S.E., 2020. Tail-propelled aquatic locomotion in a theropod dinosaur. Nature 581, 67–70. https://doi.org/10.1038/s41586-020-2190-3

Jannel, A., Salisbury, S.W., Panagiotopoulou, O., 2022. Softening the steps to gigantism in sauropod dinosaurs through the evolution of a pedal pad. Sci. Adv. 8, eabm8280. https://doi.org/10.1126/sciadv.abm8280

Kleiber, M., 1961. The Fire of Life; an Introduction to Animal Energetics, First Edition. ed. John Wiley & Sons, Inc.

Kobayashi, Y., Nishimura, T., Takasaki, R., Chiba, K., Fiorillo, A.R., Tanaka, K., Chinzorig, T., Sato, T., Sakurai, K., 2019. A New Hadrosaurine (Dinosauria: Hadrosauridae) from the Marine Deposits of the Late Cretaceous Hakobuchi Formation, Yezo Group, Japan. Sci Rep 9, 12389. https://doi.org/10.1038/s41598-019-48607-1

Köhler, M., Marín-Moratalla, N., Jordana, X., Aanes, R., 2012. Seasonal bone growth and physiology in endotherms shed light on dinosaur physiology. Nature 487, 358–361. https://doi.org/10.1038/nature11264

Kozłowski, J., Konarzewski, M., Czarnoleski, M., 2020. Coevolution of body size and metabolic rate in vertebrates: a life-history perspective. Biol Rev 95, 1393–1417. https://doi.org/10.1111/brv.12615

Krefting, L.W., 1974. The Ecology of the Isle Royale Moose with Special Reference to the Habitat.

Kwak, H.S., Im, H.G., Shim, E.B., 2016. A model for allometric scaling of mammalian metabolism with ambient heat loss. Integrative Medicine Research 5, 30–36. https://doi.org/10.1016/j.imr.2016.01.002

Laskar, A.H., Mohabey, D., Bhattacharya, S.K., Liang, M.-C., 2020. Variable thermoregulation of Late Cretaceous dinosaurs inferred by clumped isotope analysis of fossilized eggshell carbonates. Heliyon 6, e05265. https://doi.org/10.1016/j.heliyon.2020.e05265

Leblond, M., St-Laurent, M.-H., Côté, S.D., 2016. Caribou, water, and ice – fine-scale movements of a migratory arctic ungulate in the context of climate change. Mov Ecol 4, 14. https://doi.org/10.1186/s40462-016-0079-4

Lee, S.A., 2015. Metabolism of dinosaurs as determined from their growth. Phys. Rev. E 92, 032706. https://doi.org/10.1103/PhysRevE.92.032706

Maloney, S.K., Dawson, T.J., 1998. Changes in Pattern of Heat Loss at High Ambient Temperature Caused by Water Deprivation in a Large Flightless Bird, the Emu. Physiological Zoology 71, 712–719. https://doi.org/10.1086/515997

Mannion, P.D., Upchurch, P., 2010. A quantitative analysis of environmental associations in sauropod dinosaurs. Paleobiology 36, 253–282. https://doi.org/10.1666/08085.1

Mazza, P.P.A., Buccianti, A., Savorelli, A., 2019. Grasping at straws: a re-evaluation of sweepstakes colonisation of islands by mammals: Natural rafting of land mammals. Biol Rev 94, 1364–1380. https://doi.org/10.1111/brv.12506

McNab, B.K., 2009. Resources and energetics determined dinosaur maximal size. Proc. Natl. Acad. Sci. U.S.A. 106, 12184–12188. https://doi.org/10.1073/pnas.0904000106

Naish, D., 2024. The response to and rejection of Brian Ford's *Too Big to Walk* , a 21st century effort to reinstate the aquatic dinosaur hypothesis. Historical Biology 1–10. https://doi.org/10.1080/08912963.2024.2421268

O'Connor, L.K., Robinson, S.A., Naafs, B.D.A., Jenkyns, H.C., Henson, S., Clarke, M., Pancost, R.D., 2019. Late Cretaceous Temperature Evolution of the Southern High Latitudes: A TEX_{86} Perspective. Paleoceanography and Paleoclimatology 34, 436–454. https://doi.org/10.1029/2018PA003546

Ostrom, J.H., 1964. A reconsideration of the paleoecology of hadrosaurian dinosaurs. American Journal of Science 262, 975–997. https://doi.org/10.2475/ajs.262.8.975

Paladino, F.V., O'Connor, M.P., Spotila, J.R., 1990. Metabolism of leatherback turtles, gigantothermy, and thermoregulation of dinosaurs. Nature 344, 858–860. https://doi.org/10.1038/344858a0

Paul, G., 2017. Polar and K/Pg nonavian dinosaurs were low-metabolic rate reptiles vulnerable to cold-induced extinction, rather than more survivable tachyenergetic bird relatives: comment on an obsolete hypothesis. Int J Earth Sci (Geol Rundsch) 106, 2991–2998. https://doi.org/10.1007/s00531-017-1509-2

Paul, G.S., 1987. The science and art of restor-ing the life appearance of dinosaurs and their relatives: a rigorous how-to guide, in: Czerkas, S.M., Olson, E.C. (Eds.), Dinosaurs Past and Present. Natural History Museum of Los Angeles County/University of Washington Press, Seattle and Washington, pp. 5–49.

Pearson, P.N., Ditchfield, P.W., Singano, J., Harcourt-Brown, K.G., Nicholas, C.J., Olsson, R.K., Shackleton, N.J., Hall, M.A., 2001. Warm tropical sea surface temperatures in the Late Cretaceous and Eocene epochs. Nature 413, 481–487. https://doi.org/10.1038/35097000

Pontzer, H., Allen, V., Hutchinson, J.R., 2009. Biomechanics of Running Indicates Endothermy in Bipedal Dinosaurs. PLoS ONE 4, e7783. https://doi.org/10.1371/journal.pone.0007783

Prothero, J.W., 2015. The Design of Mammals: A Scaling Approach. Cambridge University Press.

Quigley, D.T.G., Moffatt, S., 2014. SIKA-LIKE DEER *CERVUS NIPPON* TEMMINCK, 1838 OBSERVED SWIMMING OUT TO SEA AT GREYSTONES, CO. WICKLOW: INCREASING DEER POPULATION PRESSURE?

Robertshaw, D., Dmi'el, R., 1983. The Effect of Dehydration on the Control of Panting and Sweating in the Black Bedouin Goat. Physiological Zoology 56, 412–418.

Ruben, J.A., Hillenius, W.J., Geist, N.R., Leitch, A., Jones, T.D., Currie, P.J., Horner, J.R., Espe, G., 1996. The Metabolic Status of Some Late Cretaceous Dinosaurs. Science 273, 1204–1207. https://doi.org/10.1126/science.273.5279.1204

Ruben, J.A., Jones, T.D., Geist, N.R., 2003. Respiratory and Reproductive Paleophysiology of Dinosaurs and Early Birds. Physiological and Biochemical Zoology 76, 141–164. https://doi.org/10.1086/375425

Ruben, J.A., Jones, T.D., Geist, N.R., 1998. Respiratory physiology of the dinosaurs. Bioessays 20, 852–859. https://doi.org/10.1002/(SICI)1521-1878(199810)20:10<852::AID-BIES11>3.0.CO;2-Q

Schmidt-Nielsen, K., 1984. Scaling: Why is Animal Size So Important? Cambridge University Press.

Schmidt-Nielsen, K., 1959. The Physiology of the Camel. Sci Am 201, 140–151. https://doi.org/10.1038/scientificamerican1259-140

Scotese, C., 2016. PALEOMAP PaleoAtlas for GPlates and the PaleoData Plotter Program (Technical Report No. http://www.earthbyte.org/paleomap-paleoatlas-for-gplates/), PALEOMAP Project. Evanston, IL. https://doi.org/10.13140/RG.2.2.34367.00166

Scotese, C.R., 2021. An Atlas of Phanerozoic Paleogeographic Maps: The Seas Come In and the Seas Go Out. Annu. Rev. Earth Planet. Sci. 49, 679–728. https://doi.org/10.1146/annurev-earth-081320-064052

Scotese, C.R., Song, H., Mills, B.J.W., Van Der Meer, D.G., 2021. Phanerozoic paleotemperatures: The earth’s changing climate during the last 540 million years. Earth-Science Reviews 215, 103503. https://doi.org/10.1016/j.earscirev.2021.103503

Scotese, C.R., Vérard, C., Burgener, L., Elling, R.P., Kocsis, A.T., 2024. The Cretaceous World: Plate Tectonics, Paleogeography, and Paleoclimate. Geological Society, London, Special Publications 544, SP544-2024–28. https://doi.org/10.1144/SP544-2024-28

Seebacher, F., 2003. Dinosaur body temperatures: the occurrence of endothermy and ectothermy. Paleobiology 29, 105–122. https://doi.org/10.1666/0094-8373(2003)029<0105:DBTTOO>2.0.CO;2

Seebacher, F., Grigg, G.C., Beard, L.A., 1999. Crocodiles as dinosaurs: behavioural thermoregulation in very large ectotherms leads to high and stable body temperatures. Journal of Experimental Biology 202, 77–86. https://doi.org/10.1242/jeb.202.1.77

Sereno, P.C., Myhrvold, N., Henderson, D.M., Fish, F.E., Vidal, D., Baumgart, S.L., Keillor, T.M., Formoso, K.K., Conroy, L.L., 2022. *Spinosaurus* is not an aquatic dinosaur. eLife 11, e80092. https://doi.org/10.7554/eLife.80092

Seymour, R.S., 2016. Cardiovascular Physiology of Dinosaurs. Physiology 31, 430–441. https://doi.org/10.1152/physiol.00016.2016

Seymour, R.S., 2013. Maximal Aerobic and Anaerobic Power Generation in Large Crocodiles versus Mammals: Implications for Dinosaur Gigantothermy. PLoS ONE 8, e69361. https://doi.org/10.1371/journal.pone.0069361

Seymour, R.S., Caldwell, H.R., Woodward, H.N., Hu, Q., 2023. Growth rate affects blood flow rate to the tibia of the dinosaur *Maiasaura*. Paleobiology 1–7. https://doi.org/10.1017/pab.2023.24

Seymour, R.S., Smith, S.L., White, C.R., Henderson, D.M., Schwarz-Wings, D., 2012. Blood flow to long bones indicates activity metabolism in mammals, reptiles and dinosaurs. Proc. R. Soc. B. 279, 451–456. https://doi.org/10.1098/rspb.2011.0968

Spotila, J.R., Lommen, P.W., Bakken, G.S., Gates, D.M., 1973. A Mathematical Model for Body Temperatures of Large Reptiles: Implications for Dinosaur Ecology. The American Naturalist 107, 391–404. https://doi.org/10.1086/282842

Vázquez López, B.J., Sellés, A., Prieto-Márquez, A., Vila, B., 2025. Habitat preference of the dinosaurs from the Ibero-Armorican domain (Upper Cretaceous, south-western Europe). Swiss J Palaeontol 144, 4. https://doi.org/10.1186/s13358-024-00346-1

Vila, B., Oms, O., Fondevilla, V., Gaete, R., Galobart, À., Riera, V., Canudo, J.I., 2013. The Latest Succession of Dinosaur Tracksites in Europe: Hadrosaur Ichnology, Track Production and Palaeoenvironments. PLoS ONE 8, e72579. https://doi.org/10.1371/journal.pone.0072579

Ward, D., 2016. The biology of deserts, Second edition. ed, The biology of habitats series. Oxford University Press, Oxford.

Weaver, J.C., 1983. The improbable endotherm: the energetics of the sauropod dinosaur *Brachiosaurus*. Paleobiology 9, 173–182. https://doi.org/10.1017/S0094837300007557

Webb, P.W., 1988. Simple Physical Principles and Vertebrate Aquatic Locomotion. Am Zool 28, 709–725. https://doi.org/10.1093/icb/28.2.709

Weishampel, D.B., Horner, J.R., 1990. Hadrosauridae, in: Weishampel, D.B., Dodson, P., Osmólska, H. (Eds.), The Dinosauria. University of California Press, pp. 534–561.

West, G.B., Brown, J.H., 2005. The origin of allometric scaling laws in biology from genomes to ecosystems: towards a quantitative unifying theory of biological structure and organization. Journal of Experimental Biology 208, 1575–1592. https://doi.org/10.1242/jeb.01589

West, G.B., Brown, J.H., Enquist, B.J., 1997. A General Model for the Origin of Allometric Scaling Laws in Biology. Science 276, 122–126. https://doi.org/10.1126/science.276.5309.122
White, C.R., Cassey, P., Blackburn, T.M., 2007. Allometric exponents do not support a universal metabolic allometry. Ecology 88, 315–323. https://doi.org/10.1890/05-1883
White, C.R., Marshall, D.J., 2023. How and Why Does Metabolism Scale with Body Mass? Physiology 38, 266–274. https://doi.org/10.1152/physiol.00015.2023
White, C.R., Seymour, R.S., 2005. Allometric scaling of mammalian metabolism. Journal of Experimental Biology 208, 1611–1619. https://doi.org/10.1242/jeb.01501
Wiemann, J., Menéndez, I., Crawford, J.M., Fabbri, M., Gauthier, J.A., Hull, P.M., Norell, M.A., Briggs, D.E.G., 2022. Fossil biomolecules reveal an avian metabolism in the ancestral dinosaur. Nature 606, 522–526. https://doi.org/10.1038/s41586-022-04770-6
Wilson, G., 1974. How kangaroos swim. The Australian journal of science 5, 11–12.
Withers, P.C., 1983. Energy, Water, and Solute Balance of the Ostrich *Struthio camelus*. Physiological Zoology 56, 568–579. https://doi.org/10.1086/physzool.56.4.30155880
