# Supplementary material for: ENHYDROSS: A New Mechanistic Model Supports the Trans‐Oceanic Dispersal Capability of Terrestrial Vertebrates
Source: Ecol Evol. 2026 Mar 30;16(4):e73280. doi: 10.1002/ece3.73280 (PMC13107292; doi:10.1002/ece3.73280)
Supplement: Supplementary file 8 — Data S8: ece373280‐sup‐0008‐SupplefileS8.pdf. [file ECE3-16-e73280-s004.pdf]

S8. Ratites

S8.1 Wettability of ratite feathers

To appreciate how long (and possibly forbidding) kilometer-scale swimming distances can be for large flightless birds we must take a look at how well they can cope in terms of heat loss when wet. While ostrich and other ratite feathers might offer a good insulation against cold temperatures on land (where air is the heat convection medium), the same is not necessarily true for wet conditions. No study that we are aware of has measured the wettability or the waterproofness of the plumage of ostriches and other ratites quantitatively. However, it can be safely assumed that their feathers are not waterproof nor particularly water repellent. This can be deduced from the following lines of evidence. First, Struthioniformes lack the preen-oil producing uropygial (coccygeal) gland possessed by other birds, and best developed in waterfowl, that has been hypothesized by some to increase the water-repellent properties of avian feathers (Chernova and Fadeeva, 2009; Moreno-Rueda, 2017; Muzio and Rubega, 2024). This may occur directly through the preen-oil's water-repellent properties or indirectly by preventing feather degradation (Moreno-Rueda, 2017 and references therein). Second, ratite feathers do not have the same structural properties that enable water fowl plumage to be waterproof or strongly hydrophobic. Such properties are related to barb diameter (2r) and interspacing distance (2d), where the relation (r+d)/r is higher for feathers that have good water repellent (hydrophobic) properties (up to a limit) and lower for plumage that has strong resistance to water penetration (waterproof) (Rijke, 1970) (but see discussion in Muzio and Rubega, 2024)). Given that that barbs lack interlocking barbules in the ostrich (Bezuidenhout, 1999) and in the emu and cassowary (Chernova and Fadeeva, 2009) that otherwise keep the barbs tightly close together to prevent water penetration, it is reasonable to assume that water can penetrate through the barbs and contact the skin. Third, the emu's feathers have low hydrophobicity while additionally, they are covered by a specialized spongy structure which among other things serves as a water retention unit after the animal exits a body of water (Chernova and Fadeeva, 2009). This may help to prevent dehydration via cutaneous evaporation in its hot and dry natural habitat (Chernova and Fadeeva, 2009). Thus, if anything, this has the opposite effect of being waterproof. This feature has not been observed in other ratites, with the exception of the cassowary (Chernova and Fadeeva, 2009). Beyond lacking waterproof plumage, in the case of ostriches in particular, at least half of their body's wetted surface area when floating (see Figure 2) is occupied by its nearly featherless legs and often relatively lightly feathered belly; other ratites however have more plumage covered area on their legs' surface (Cho et al., 1984). Furthermore, the ostrich (and likely other ratites) has very little fat (mean total fat from 14 individuals is 5.18% of body mass (Morris et al., 1995)) that cannot be used as an effective insulator when in contact with water. Hence, much like the rheas in the aforementioned case, the ostrich and its flightless relatives are expected to pay a heavy toll in terms of energy cost for thermogenesis (M_T) in the hypothetical scenario of a crossing of a narrow sea, let alone an ocean. Given the above, we suggest that our model's assumption about M_T being 0 will not likely hold in this case. As such, we speculate that such an animal is likely to die from hypothermia well before it falls victim to energy exhaustion, at least in relatively low water temperatures (<25 °C), thereby making such animals poor long-distance swimmers and thus oceanic dispersers.

S8.2. Video links for swimming ratites

Taxon	Online video links	Last accessed
Ostrich	https://www.youtube.com/watch?v=wFQDu47MNSU	28/5/2025
	https://www.youtube.com/watch?v=IAs3KCJKfoM	28/5/2025
	https://www.youtube.com/watch?v=k9UqxMx4eIo	28/5/2025
Emu	https://www.youtube.com/watch?v=K2gwC6nU9fw	28/5/2025
	https://www.youtube.com/watch?v=x-diavKXAn0&ab_channel=GeraldO%27Leary	28/5/2025
Greater rhea	https://www.youtube.com/watch?v=CWXM0YnoLdQ	28/5/2025
Lesser rhea	https://www.youtube.com/watch?v=iRmiHmCCiDE&ab_channel=AnnickMorgenthaler	28/5/2025
Cassowary	https://www.youtube.com/watch?v=pYr3zYwImfM&ab_channel=GuardianAustralia	28/5/2025

Table S8.1. Links to online videos showing swimming ratites.

References

Bezuidenhout, A.J., 1999. Anatomy, in: Deeming, D.C. (Ed.), The Ostrich: Biology, Production, and Health. CABI Pub, Wallingford, Oxon, UK ; New York, NY, USA, pp. 13–50.

Chernova, O.F., Fadeeva, E.O., 2009. The peculiar architectonics of contour feathers of the emu (*Dromaius novaehollandiae*, Struthioniformes). Dokl Biol Sci 425, 175–179. https://doi.org/10.1134/S0012496609020264

Cho, P., Brown, R., Anderson, M., 1984. Comparative gross anatomy of ratites. Zoo Biology 3, 133–144. https://doi.org/10.1002/zoo.1430030205

Moreno-Rueda, G., 2017. Preen oil and bird fitness: a critical review of the evidence. Biological Reviews 92, 2131–2143. https://doi.org/10.1111/brv.12324

Morris, C.A., Harris, S.D., May, S.G., Jackson, T.C., Hale, D.S., Miller, R.K., Keeton, J.T., Acuff, G.R., Lucia, L.M., Savell, J.W., 1995. Ostrich Slaughter and Fabrication: 1. Slaughter Yields of Carcasses and Effects of Electrical Stimulation on Post-Mortem pH. Poultry Science 74, 1683–1687. https://doi.org/10.3382/ps.0741683

Muzio, F.M.S., Rubega, M.A., 2024. What do we really know about the water repellency of feathers? Journal of Avian Biology e03259. https://doi.org/10.1111/jav.03259

Rijke, A.M., 1970. Wettability and Phylogenetic Development of Feather Structure in Water Birds. Journal of Experimental Biology 52, 469–479. https://doi.org/10.1242/jeb.52.2.469
